# GFRA2 Identifies Cardiac Progenitors and Mediates Cardiomyocyte Differentiation in a RET-Independent Signaling Pathway

**DOI:** 10.1016/j.celrep.2016.06.050

**Published:** 2016-07-07

**Authors:** Hidekazu Ishida, Rie Saba, Ioannis Kokkinopoulos, Masakazu Hashimoto, Osamu Yamaguchi, Sonja Nowotschin, Manabu Shiraishi, Prashant Ruchaya, Duncan Miller, Stephen Harmer, Ariel Poliandri, Shigetoyo Kogaki, Yasushi Sakata, Leo Dunkel, Andrew Tinker, Anna-Katerina Hadjantonakis, Yoshiki Sawa, Hiroshi Sasaki, Keiichi Ozono, Ken Suzuki, Kenta Yashiro

**Affiliations:** 1 Centre for Endocrinology, William Harvey Research Institute, Barts and The London School of Medicine and Dentistry, Queen Mary University of London, London EC1M 6BQ, UK; 2 Translational Medicine and Therapeutics, William Harvey Research Institute, Barts and The London School of Medicine and Dentistry, Queen Mary University of London, London EC1M 6BQ, UK; 3 Cardiac Electrophysiology, William Harvey Research Institute, Barts and The London School of Medicine and Dentistry, Queen Mary University of London, London EC1M 6BQ, UK; 4 Department of Paediatrics, Osaka University Graduate School of Medicine, Osaka 565-0871, Japan; 5 Department of Cardiovascular Medicine, Osaka University Graduate School of Medicine, Osaka 565-0871, Japan; 6 Department of Cardiovascular Surgery, Osaka University Graduate School of Medicine, Osaka 565-0871, Japan; 7 Laboratory for Embryogenesis, Osaka University Graduate School of Frontier Biosciences, Osaka 565-0871, Japan; 8 Developmental Biology Program, Memorial Sloan Kettering Cancer Center, New York, NY 10065, USA; 9 Centre of Human and Aerospace Physiological Sciences, School of Biomedical Sciences, King’s College, London, SE1 1UL, UK

## Abstract

A surface marker that distinctly identifies cardiac progenitors (CPs) is essential for the robust isolation of these cells, circumventing the necessity of genetic modification. Here, we demonstrate that a Glycosylphosphatidylinositol-anchor containing neurotrophic factor receptor, Glial cell line-derived neurotrophic factor receptor alpha 2 (*Gfra2*), specifically marks CPs. GFRA2 expression facilitates the isolation of CPs by fluorescence activated cell sorting from differentiating mouse and human pluripotent stem cells. *Gfra2* mutants reveal an important role for GFRA2 in cardiomyocyte differentiation and development both in vitro and in vivo. Mechanistically, the cardiac GFRA2 signaling pathway is distinct from the canonical pathway dependent on the RET tyrosine kinase and its established ligands. Collectively, our findings establish a platform for investigating the biology of CPs as a foundation for future development of CP transplantation for treating heart failure.

## Highlights

• *Gfra2* is specific for cardiac progenitors (CPs) among mesodermal cells
• GFRA2 enables isolation of multipotent or unipotent CPs in mouse and human
• *Gfra2* and the related molecule *Gfra1* play a vital role in heart development
• The GFRA1/2 signal in heart development is independent of established ligands

## Graphical Abstract

**Figure:**
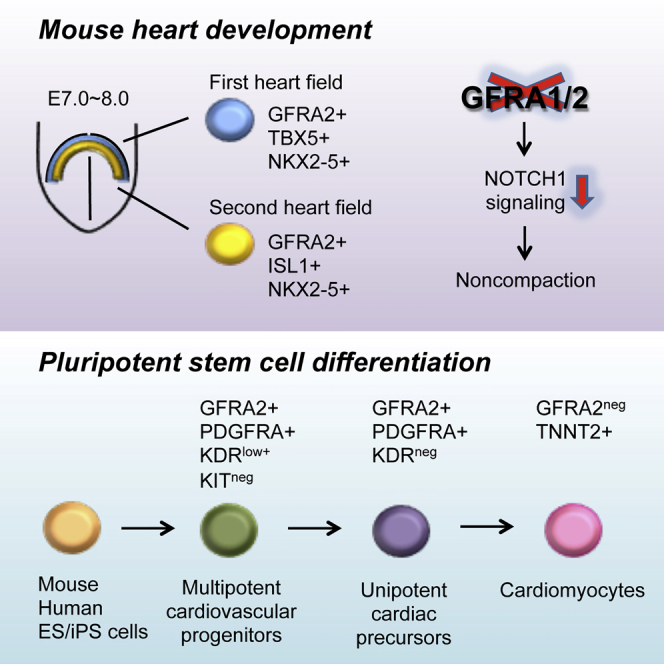

## Teaser

Ishida et al. show that GPI-anchored neurotrophic factor receptor *Gfra2* specifically marks cardiac progenitor cells (CPs) in mouse and human, providing a method for isolating CPs. Unexpectedly, *Gfra2* plays a significant role in heart development via a non-canonical signaling pathway that is independent of known ligands and the co-receptor RET tyrosine kinase.

## Introduction

The heart is the first morphologically distinct developing organ in vertebrates. The primordial heart is derived from the anterior part of the lateral plate mesoderm as cardiac progenitors (CPs) being one of the earliest populations emerging from the primitive streak at gastrulation (Kinder et al., 1999, Rana et al., 2013). Lineage tracing experiments have led to the identification of CPs in the first (FHF) and second heart field (SHF) according to their anatomical origin and destiny (Rana et al., 2013). Recently, studies have delineated the complex molecular mechanisms underlying cardiomyocyte differentiation (Kathiriya et al., 2015, Paige et al., 2015); however, our knowledge of the precise spatiotemporal mechanisms that regulate the segregation, identity, and fate of CPs remains incomplete. A major hurdle is the paucity of reliable and specific markers to identify CPs, especially for the robust isolation of living CPs using cell sorting, circumventing the requirement of genetic modification for tagging CPs. Previous reports have demonstrated that Kinase insert domain receptor (KDR, also known as Flk-1), platelet-derived growth factor receptor alpha (PDGFRA), KIT, C-X-C chemokine receptor type 4 (CXCR4), and/or Prion protein (PrnP) can be used in defined combinations to identify and harvest CPs (Bondue et al., 2011, Hidaka et al., 2010, Kattman et al., 2006, Kattman et al., 2011, Nelson et al., 2008, Yang et al., 2008). In vitro clonal-tracing studies have revealed that both KDR^+^/PDGFRA^+^ and KDR^low+^/KIT^neg^ cell populations contain highly enriched multipotent progenitors producing not only cardiomyocytes but also endothelial and smooth muscle cells in mouse and human, respectively (Kattman et al., 2006, Kattman et al., 2011, Yang et al., 2008). Moreover, since the expression pattern of each of these factors in the embryo is dynamic and not specific for the cardiac lineage (Hidaka et al., 2010, Kataoka et al., 1997, McGrath et al., 1999, Yang et al., 2008), concerns have been raised about the purity of CPs harvested using these markers. More recently, a cell-surface protein, hyperpolarization-activated cyclic nucleotide-gated potassium channel 4 (HCN4), has been reported to be transiently specific for FHF CPs during the earliest phase of cardiomyogenesis (Später et al., 2013). However, because there is no commercially available antibody against the extracellular domain of this molecule, its use in cell-sorting experiments is limited. Thus, identification of a CP-specific surface antigen for which an antibody is readily available is essential for furthering our understanding of the critical early events in heart development.

In this study, we found that Glial cell line-derived neurotrophic factor receptor alpha 2 (*Gfra2*) specifically marks CPs of the FHF and SHF in mouse and human (Airaksinen and Saarma, 2002, Paratcha and Ledda, 2008). The specificity and expression pattern of *Gfra2* provides a reliable means to isolate stage-specific CPs with high purity. Strikingly, *Gfra2* is essential for heart development, whereas *Gfra1*, another member of the *Gfra* receptor family, is functionally redundant. Finally, we demonstrate that the pathway by which GFRA1/2 modulates heart development is independent of the classical *Gfra* receptor family signaling pathway via the RET proto-oncogene.

## Results

### *Gfra2* Specifically Marks Both FHF and SHF CPs

**Figure 1:**
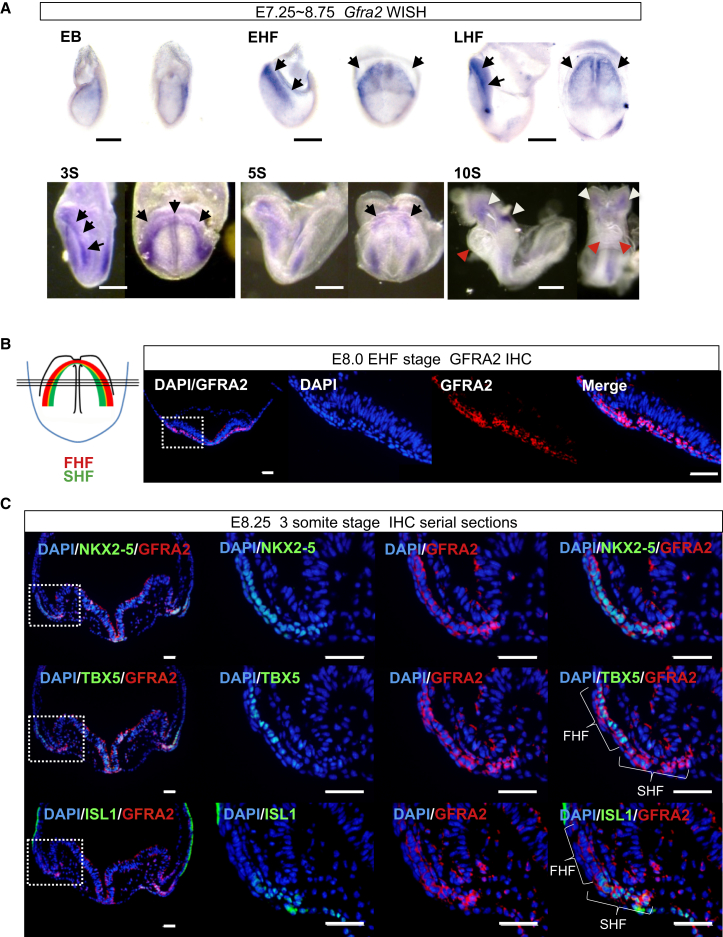
GFRA2 Is Expressed in the First and Second Heart Field Cardiac Progenitor Cells (A) Whole-mount in situ hybridization (WISH) of *Gfra2* in E7.25–8.75 early allantoic bud (EB) stage to the ten-somite (S) stage embryos. *Gfra2* was expressed in the cardiac crescent (arrows). Once the heart tube is formed and the looping initiated, *Gfra2* is downregulated (red arrowheads). *Gfra2* was also expressed in migrating neural crest cells and the rhombomere 4 (white arrowheads). n = 3. Scale bar, 250 μm. EHF, early headfold; LHF, late headfold. (B and C) Immunohistochemical (IHC) images of GFRA2 in an EHF and three-somite stage embryo. GFRA2 was expressed in the mesodermal regions corresponding to the heart field. n = 4. Scale bar, 50 μm. FHF, first heart field; SHF, second heart field. See also Figure S1.

According to previous single-cell expression profiling of mouse embryonic CPs between days 7.5 and 8.0 post-conception (E7.5–E8.0), we observed that *Gfra2*, a specific receptor for a neurotrophic factor Neurturin (NRTN), was expressed in CPs but not in embryonic stem cells (ESCs) (42.31 ± 22.53 SEM of CPs versus 0.00 of ESCs in Reads Per Millions, respectively) (Brouilette et al., 2012, Kokkinopoulos et al., 2015). This was consistent with the data demonstrating that *Gfra2* was co-expressed within the cardiac mesoderm expressing *Mesp1* (Bondue et al., 2011). To confirm the expression pattern of *Gfra2* in mouse embryos, we conducted whole-mount in situ hybridization (WISH) analyses in serial stages of early mouse embryos and found that *Gfra2* was detected from the Early Allantoic Bud stage simultaneously with one of the earliest markers of CPs, *Isl1*, and was clearly expressed in the cardiac crescent from E7.5 to E8.5 (Figures 1A and S1A) (Downs and Davies, 1993, Kokkinopoulos et al., 2015). Single-cell expression profiling suggests that *Isl1* precedes *Gfra2*, because *Isl1*-expressing CPs had a higher incidence of expression of *Gfra2* after they had started to express a common marker for the FHF and SHF, *Nkx2-5*, as they further differentiated (Figure S1B) (Kokkinopoulos et al., 2015). Thereafter, *Gfra2* was downregulated in the heart field by the ten-somite stage (E8.75) upon formation of the heart tube (Figure 1A). Immunofluorescence micrographs indicated that GFRA2 protein was prominently detected in the early headfold (EHF) stage CPs (Figure 1B) (Downs and Davies, 1993). Serial sections of the three-somite stage embryos revealed that GFRA2 co-localized with NKX2-5, TBX5, HCN4 (the FHF), and ISL1 (the SHF) (Figures 1C and S1C) (Cai et al., 2003, Devine et al., 2014, Kokkinopoulos et al., 2015, Liang et al., 2013, Später et al., 2013, Stanley et al., 2002). Therefore, GFRA2 can be considered a marker of CPs within both the FHF and SHF in mouse embryos.

### GFRA2 Predominantly Identifies CPs Derived from Pluripotent Stem Cells

**Figure 2:**
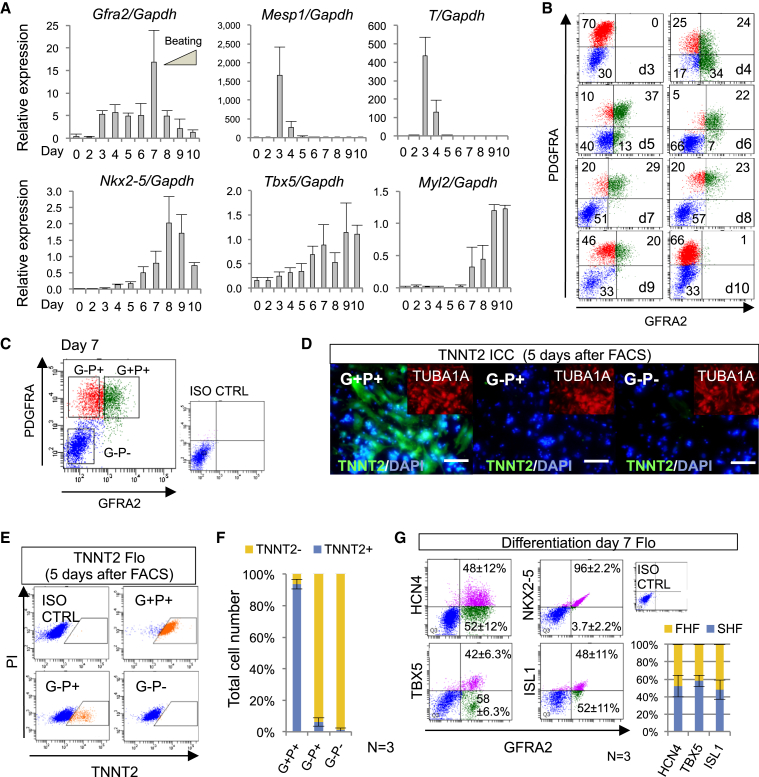
GFRA2^+^/PDGFRA^+^ Cells Derived from Mouse ESCs Are Unipotent Cardiac Precursors (A) qPCR analyses of *Gfra2*, *Mesp1*, *T* (*Brachyury*), *Nkx2-5*, *Tbx5*, and *Myl2*. The peak of *Gfra2* expression was observed just before the initiation of spontaneous beating of cardiomyocytes. Note that mesodermal induction represented by *Mesp1* and *T* peaked at day 3 and sarcomeric protein synthesis of cardiomyocyte was apparent from day 8. Data are representative of biological triplicates with technical duplicates as mean ± SEM. (B) Flow cytometrical (Flo) analyses show transient expression of GFRA2 during cardiomyocyte differentiation. GFRA2 was detected in the PDGFRA^+^ mesodermal population from day 4 to day 9. (C) FACS isolation at day 7 of differentiation. GFRA2^+^/PDGFRA^+^ (G^+^P^+^), GFRA2^−^/PDGFRA^+^ (G^−^P^+^), GFRA2^−^/PDGFRA^−^ (G^−^P^−^) populations were separately isolated. (D) Immunocytochemistry (ICC) of TNNT2 in FACS-isolated cells after an additional 5 days culture in differentiation media. The condensed high DAPI signals represent dead cells. We counterstained with TUBA1A (α-tubulin) to delineate the live cells and found that most of G^+^P^+^ cells differentiated into TNNT2^+^ cardiomyocytes. Scale bar, 100 μm. (E and F) Flow cytometrical (Flo) analyses for TNNT2 revealed that most of the G^+^P^+^ cells (93.6% ± 3.1%) differentiated into cardiomyocytes. n = 3. (G) Quantitative analyses by Flo for HCN4, NKX2-5, TBX5, and ISL1 in GFRA2^+^ CPs at day 7. Bar graph represents of the proportion of FHF and SHF as mean ± SEM. n = 3. See also Figure S2 and Movie S1.

Next, to clarify whether GFRA2 marks CPs derived from pluripotent stem cells, we investigated the expression of *Gfra2* during cardiac differentiation of mouse ESCs. Quantitative real-time reverse transcription PCR (qPCR) demonstrated that *Gfra2* was transiently expressed with its peak at differentiation day 7, before the initiation of spontaneous beating of differentiated cardiomyocytes (Figure 2A). Flow cytometry using an antibody raised against the extracellular domain of GFRA2 revealed that GFRA2 could be identified between differentiation days 4–9, among the PDGFRA positive mesodermal cells (Figures 2B and S2A). Upon culturing the separately isolated cell populations of GFRA2^+^/PDGFRA^+^, GFRA2^neg^/PDGFRA^+^, and GFRA2^neg^/PDGFRA^neg^ at differentiation day 7 by fluorescent activated cell sorting (FACS), the majority of GFRA2^+^/PDGFRA^+^ cells differentiated into TNNT2^+^ and ACTN1 (α-ACTININ)^+^ cardiomyocytes, without a propensity for differentiation to endothelial cells and smooth muscle cells (Figures 2C–2F, S2B, and S2C; Movie S1). By contrast, the other cell populations rarely contained such cardiac cells. These results suggest that GFRA2^+^/PDGFRA^+^ cells at day 7 are already committed to a cardiomyocyte fate but remain as precursor cells without terminal differentiation. The expression of NKX2-5 (common), TBX5 (FHF), HCN4 (FHF), and ISL1 (SHF) in GFRA2^+^/PDGFRA^+^ cells demonstrated that GFRA2^+^/PDGFRA^+^ CPs reside in both the FHF and SHF (Figures 2G and S2D) (Cai et al., 2003, Devine et al., 2014, Kokkinopoulos et al., 2015, Liang et al., 2013, Später et al., 2013, Stanley et al., 2002). These data were consistent with our histological results in mouse embryos (Figures 1C and S1C).

**Figure 3:**
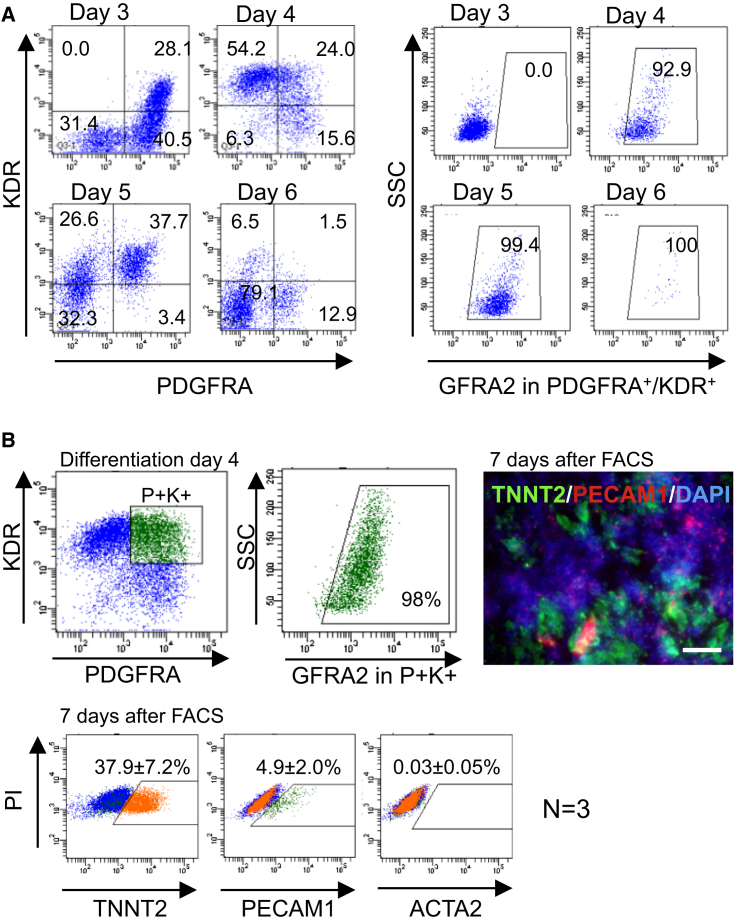
GFRA2^+^/KDR^+^/PDGFRA^+^ Represents Multipotent CPs (A) Flo analyses for KDR, PDGFRA, and GFRA2. KDR^+^/PDGFRA^+^ cells were observed at day 3, but these were GFRA2 negative. After day 4, most of KDR^+^/PDGFRA^+^ CPs were GFRA2 positive (Kattman et al., 2011). KDR expression was significantly downregulated at day 6. (B) FACS isolation of GFRA2^+^/KDR^+^/PDGFRA^+^ population at differentiation day 4. Immunocytochemical and flow cytometrical analyses for TNNT2, PECAM1, and ACTA2 (α- smooth muscle cell actin) reveal that this population differentiated into both cardiomyocytes and endothelial cells. This is consistent with the previous study (Kattman et al., 2011), which shows this population is multipotent cardiovascular progenitors. Scale bar, 100 μm. See also Figure S2.

To further characterize GFRA2^+^ cells during cardiac differentiation, we investigated the relationship between GFRA2-expressing CPs and the well-validated earliest CPs of the KDR^+^/PDGFRA^+^ population in differentiating mouse ESCs (Kattman et al., 2006, Kattman et al., 2011). KDR and PDGFRA were already expressed by day 3 of differentiation as previously described (Kattman et al., 2011), and KDR expression was downregulated in the PDGFRA^+^ population at day 6 (Figure 3A). Of note, from day 4 to 5, almost the entire KDR^+^/PDGFRA^+^ population expressed GFRA2. Thus, GFRA2 could also mark the earliest mouse CPs in cardiac differentiating ESCs. When we isolated a GFRA2^+^/KDR^+^/PDGFRA^+^ triple-positive population on day 4 and cultured the cells for a further 7 days, they gave rise not only to cardiomyocytes, but also to endothelial cells (Figure 3B). Given that GFRA2 marked KDR^+^/PDGFRA^+^ CPs on differentiation day 4, the earliest GFRA2^+^ CPs would be expected to be multipotent based on previous reports (Bondue et al., 2011, Kattman et al., 2011). Thereafter, KDR expression would be limited to the endothelial lineage, with cardiomyogenic cells having lost KDR expression after day 6 of differentiation (Figures 3A, S2E, and S2F). Taken together, our findings clearly demonstrate that GFRA2 facilitates the robust isolation of CPs from differentiating mouse ESCs.

### GFRA2 Marks Human CPs from Differentiating Human Pluripotent Stem Cells

**Figure 4:**
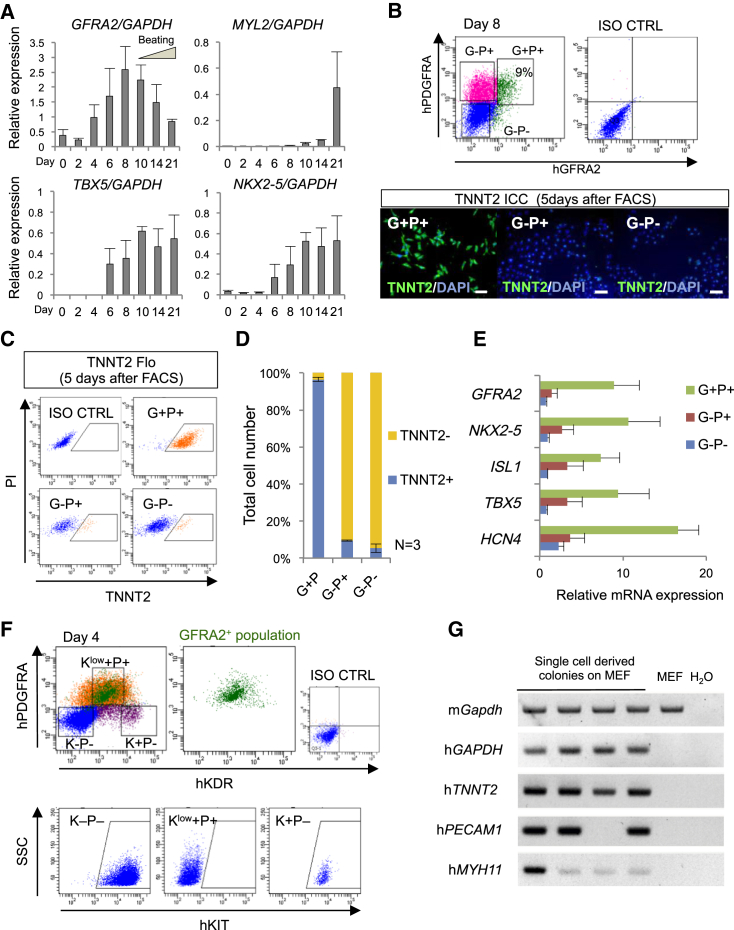
Human GFRA2 Marks CPs from Human ESC Cultures (A) qPCR analyses for human *GFRA2*, *NKX2-5*, *TBX5*, and *MYL2*. Note the peak of human *GFRA2* expression is just before human embryoid bodies start to beat. Bar graph represents biological triplicates with technical duplicates as mean ± SEM. (B) FACS isolation of human hGFRA2^+^/hPDGFRA^+^ (G^+^P^+^) cells. ICC analyses for TNNT2 demonstrate that isolated G^+^P^+^ population is highly cardiomyogenic. Scale bar, 100 μm. (C and D) Quantitative analyses by Flo for TNNT2 show most of G^+^P^+^ cells (96.5% ± 1.1%) differentiated into cardiomyocytes. Bar graph represents mean ± SEM. n = 3. (E) qPCR analyses for *GFRA2*, *NKX2-5*, *ISL1*, *TBX5*, and *HCN4* in FACS-isolated populations. All cardiac marker gene expressions were significantly higher in the G^+^P^+^ population when compared to the G^−^P^−^ population (^∗^p < 0.05, Student’s t test). Data are representative of biological triplicates with technical duplicates as mean ± SEM. (F) Flow cytometry of human ESCs at differentiation day 4. hGFRA2^+^ cells (shown as green dots) were mostly included by hPDGFRA^+^/hKDR^low+^/hKIT^neg^ population, which is reported as multipotent CPs (Yang et al., 2008). 15%–20% of hPDGFRA^+^/hKDR^low+^/hKIT^neg^ cells were hGFRA2^+^. (G) RT-PCR analyses of single-cell-derived colonies for each lineage marker gene. A single hGFRA2^+^/hKDR^low+^/hPDGFRA^+^ cell at day 4 was clonally sorted and cultured on mouse embryonic fibroblasts (MEFs) for 2 weeks. RT-PCR of *hTNNT2*, *hPECAM1*, and *hMYH11* represent the existence of cardiomyocytes, endothelial cells, and smooth muscle cells among the cells derived from a single cell, respectively. Note the existence of multiple lineages, which indicates the multipotency. See also Figure S3 and Movie S2.

To challenge whether human GFRA2 can be used for CP isolation from human pluripotent stem cells, we investigated the expression of human *GFRA2* during the cardiac differentiation of human ESCs or induced pluripotent stem cells (iPSCs) (Burridge et al., 2011). Consistent with our results for the mouse, qPCR analyses showed human *GFRA2* was induced with a peak just before the appearance of spontaneously beating cardiomyocytes at day 8 of differentiation, both in ESCs and iPSCs (Figures 4A and S3A). We also identified the hGFRA2^+^/hPDGFRA^+^ cell population at day 8 by flow cytometry, with FACS-isolated hGFRA2^+^/hPDGFRA^+^ cells efficiently differentiating into TNNT2^+^ cardiomyocytes when cultured for an additional 5 days (Figures 4B–4D and S3B–S3D). These TNNT2^+^ cardiomyocytes demonstrated spontaneous beating (Movie S2). The expression profiles of FACS-purified hGFRA2^+^/hPDGFRA^+^ cells suggest that this population contains both the FHF and SHF CPs, similarly as in the case of mouse ESCs (Figures 2G, 4E, and S2D). Thus, labeling with antibodies raised to human GFRA2 also enables the isolation of a CP population from human pluripotent stem cells, without the need of lineage tagging by genetic modification.

To elucidate additional details concerning the earliest phase of hGFRA2^+^ CPs in human cardiac differentiation, we performed flow cytometry using hGFRA2, hPDGFRA, hKDR, and hKIT antibodies at day 4 of differentiation. We found that the proportion of hGFRA2-expressing cells among the multipotent CP-enriched population of hKDR^low+^/hPDGFRA^+^ cells were less than in the case of mouse (Figures 3A and 4F) (Kattman et al., 2011). As expected, the hKDR^low+^/hPDGFRA^+^ population was hKIT negative, whereas hKDR^+^/hPDGFRA^neg^ population was hKIT positive (Figure 4F) (Yang et al., 2008). By separating isolated hGFRA2^+^/hKDR^low+^/hPDGFRA^+^/hKIT^neg^ and hGFRA2^neg^/hKDR^low+^/hPDGFRA^+^/hKIT^neg^ populations, we found that the GFRA2 negative population almost lacked cardiomyogenic ability (Figure S3E). To confirm multipotency of human GFRA2 positive cells, we performed clonal lineage-tracing experiments. A single hGFRA2^+^/hKDR^low+^/hPDGFRA^+^/hKIT^neg^ cell at day 4 was cloned by FACS and cultured for 2 weeks. RT-PCR using cardiomyocyte (*hTNNT2*), endothelial cell (*hPECAM*), and smooth muscle cell (*hMYH11*) markers clearly indicated the existence of the multiple cell lineages derived from a single cell, which strongly supports the multipotency of hGFRA2^+^/hKDR^low+^/hPDGFRA^+^/hKIT^neg^ cells (Figure 4G). This is consistent with the data from mouse ESCs and previous work (Kattman et al., 2006, Kattman et al., 2011, Yang et al., 2008). After differentiation day 4, as cardiomyocyte differentiation progressed, GFRA2^+^/PDGFRA^+^ CPs lost KDR expression by day 8 as observed in mouse ESCs differentiation (Figure S3F). Taken together, as in the mouse, hGFRA2^+^/hKDR^low+^/hPDGFRA^+^/hKIT^neg^ CPs at day 4 are multipotent CPs, and hGFRA2^+^/hKDR^neg^/hPDGFRA^+^/hKIT^neg^ at day 8 are unipotent cardiac precursors.

### A Non-canonical Signaling Cascade via GFRA1/2 Is Indispensable for Cardiomyocyte Differentiation of Pluripotent Stem Cells

**Figure 5:**
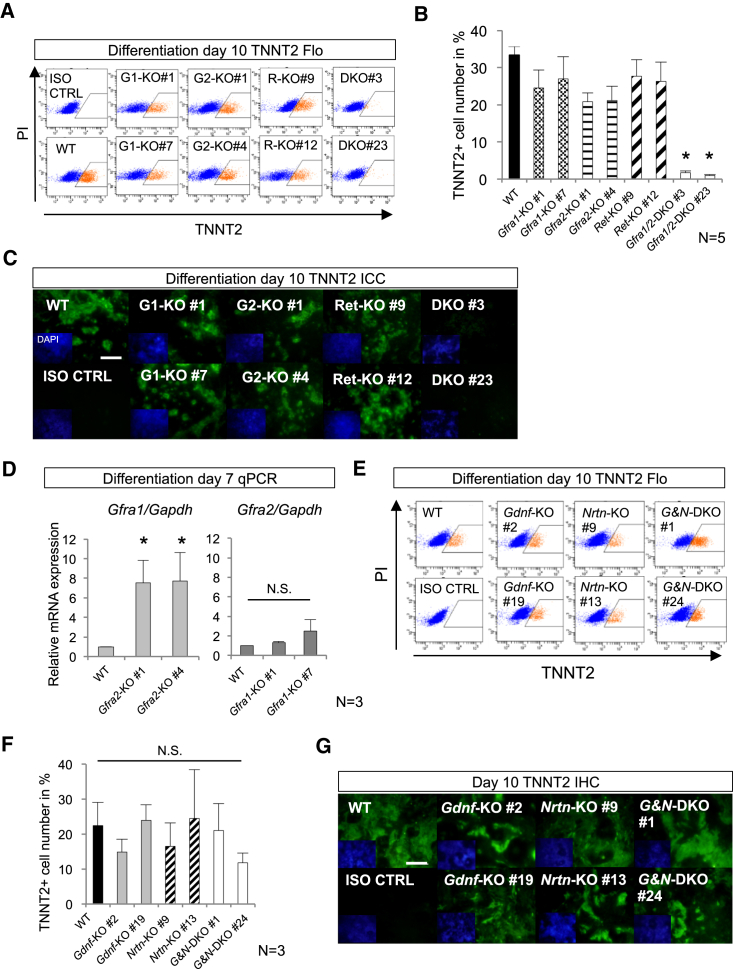
*Gfra1* and *Gfra2* Are Essential for In Vitro Cardiomyocyte Differentiation from Mouse ESCs (A) Flo analyses of TNNT2 at day 10 of differentiation. WT, wild-type; G1-KO, *Gfra1*-KO; G2-KO, *Gfra2*-KO; DKO, *Gfra1/2*-DKO; R-KO, *Ret*-KO; ISO CTRL, isotype control. (B) Quantitative analyses of flow cytometry show the severe impairment of cardiomyocyte differentiation in *Gfra1/2* DKO ESC lines. ^∗^p < 0.05 versus WT in Student’s t test. n = 5. (C) Immunocytochemical analyses of each KO ESC line 10 days after induction of cardiomyocyte differentiation. *Gfra1/2* double-KO (DKO) ESCs exhibited severe defects in TNNT2^+^ cardiomyocyte differentiation. Scale bar, 100 μm. (D) qPCR analyses for *Gfra1* and *Gfra2* in *Gfra2* KO ESC lines and in *Gfra1* KO ESC line at day 7, respectively. Note the elevated expression of *Gfra1* in *Gfra2* KO ESCs. ^∗^p < 0.05 versus WT in Student’s t test. N.S., not significant. Bar graph represents mean ± SEM. n = 3. (E and F) Flow cytometrical analyses of TNNT2 in differentiation day 10 ESC lines. *G&N*, *Gdnf*, and *Nrtn*. *Gdnf-* and *Nrtn*-null ESC lines did not show any statistically significant difference in cardiomyocyte differentiation efficiency compared to WT. N.S., not significant versus WT. Bar graph represents mean ± SEM. n = 3. (F) Immunofluorescent images of TNNT2 in ESC lines at differentiation day 10. Scale bar, 100 μm. (G) See also Figures S4–S6.

To elucidate a physiological function of GFRA2 in cardiac differentiation, we generated *Gfra2* knockout (KO) mouse ESC lines using the CRISPR/Cas9 genome editing system (Figures 5 and S4) (Cong et al., 2013, Wang et al., 2013). After 10 days of cardiac differentiation, two independent lines of *Gfra2-*KO ESCs did not show significant defects in cardiomyocyte differentiation, although a minor decrease in the number of differentiated cardiomyocytes was observed without statistical significance (Figures 5A–5C). This is consistent with the phenotype of KO mice that showed no cardiac defects (Airaksinen and Saarma, 2002, Hiltunen et al., 2000, Paratcha and Ledda, 2008, Rossi et al., 1999, Rossi et al., 2003). Since GFRA1, another member of GFRA-family receptor whose specific ligand is glial cell line-derived neurotrophic factor (GDNF), might be functionally redundant, we generated compound mutant of *Gfra1/2* double-KO (DKO) ESCs (Airaksinen and Saarma, 2002, Baloh et al., 2000). Whereas *Gfra1* KO lines exhibited no significant defect in cardiomyocyte differentiation (Enomoto et al., 1998), the simultaneous ablation of *Gfra1* in addition to *Gfra2* significantly suppressed cardiomyocyte differentiation (Figures 5A–5C and S4A–S4E). It is unlikely that an off-target mutation is responsible for this phenotype, because two independent single guidance (sg) RNA targeting different portions of the gene resulted in an indistinguishable phenotype (Figures S4C and S4D) (Enomoto et al., 1998, Fu et al., 2013, Hiltunen et al., 2000, Rossi et al., 1999, Rossi et al., 2003). Thus, *Gfra1/2* are required for cardiomyocyte differentiation in vitro, and *Gfra1* is functionally redundant for *Gfra2*. Interestingly, we found that *Gfra1* expression was significantly increased in *Gfra2* KO ESCs (Figure 5D). By contrast, *Gfra2* expression was unchanged in *Gfra1* KO ESCs. This result suggests that the loss of *Gfra2* can be compensated for by upregulated *Gfra1*, whereas *Gfra1* appears to be dispensable for cardiac differentiation (Baloh et al., 2000, Paratcha and Ledda, 2008, Scott and Ibanez, 2001). This suggestion is supported by the fact that *Gfra1* is not expressed in the heart field in vivo (Figure S5A).

The canonical signaling cascade acting via GFRA2 depends on a single-pass transmembrane protein, RET tyrosine kinase (Airaksinen and Saarma, 2002). When the specific ligand, neurturin (NRTN) binds GFRA2, the RET tyrosine kinase is activated by GFRA2/NRTN, to elicit a biological response. To confirm whether the GFRA2 signaling pathway affecting cardiomyocyte differentiation depends on RET, we generated *Ret* KO ESCs lines. As expected from the phenotypes of KO mice, targeting of *Ret* did not resulted in significantly impaired cardiomyocyte differentiation (Figures 5A–5C, S4A, and S4B) (Airaksinen and Saarma, 2002, Baloh et al., 2000, Paratcha and Ledda, 2008, Schuchardt et al., 1994). This observation is also supported by the fact that RET was not expressed in the heart fields (Figure S5A). In addition, the KO lines of *Nrtn*, *Gdnf*, and *Nrtn*/*Gdnf* also did not show a significant defect (Figures 5E–5G, S5B, and S5C) (Golden et al., 1999, Heuckeroth et al., 1999, Sánchez et al., 1996). Collectively, these results indicate that cardiac differentiation signaling via GFRA2 is independent of the co-receptor RET tyrosine kinase.

Previous studies have reported that the direct interaction between Neural Cell Adhesion Molecule (NCAM1) and GFRA1 mediates an alternative GFRA1 signaling pathway via FAK/FYN operating in the absence of any secreted ligands and RET (Paratcha and Ledda, 2008, Paratcha et al., 2003, Sjöstrand et al., 2007). Although NCAM1 was not expressed in the heart field (Figure S5A), we cannot exclude the possibility that another cell adhesion molecule mediates a similar signal pathway. To confirm whether a similar pathway is responsible for the cardiac signaling of GFRA2, we investigated the phosphorylation of FAK, FYN, and its downstream ERK1/2 in *Gfra1/2* DKO ESCs during cardiomyocyte differentiation (Figure S6). Western blot analyses demonstrated that FAK phosphorylation was slightly but significantly elevated in *Gfra1/*2 DKO ESCs, whereas FYN and ERK1/2 phosphorylation were unaffected (Figure S6A). Since the NCAM1/GFRA1 signal pathway first activates FYN and phosphorylated-FYN activates FAK (Paratcha et al., 2003), it is unlikely that a signal similar to the NCAM1/GFRA1 signal mediated by a cell adhesion molecule is operating. Thus, it suggests that FAK phosphorylation in *Gfra1/*2 DKO ESCs becomes elevated by an unknown mechanism. We further tested whether the attenuation of elevated FAK could rescue the phenotype of *Gfra1/*2 DKO ESCs, since it has been previously reported that activated FAK signaling impaired cardiomyocyte differentiation (Hakuno et al., 2005). We administered the FAK inhibitor PF-573228 to *Gfra1/*2 DKO ESCs during their differentiation. However, the efficiency of cardiomyocyte differentiation showed no improvement even though FAK phosphorylation was kept within physiological levels (Figures S6B and S6C). Therefore, the upregulation of FAK signaling was not primarily responsible for the defect of *Gfra1/2* DKO ESCs. This signaling pathway operating via GFRA2 during cardiomyogenesis must activate effectors of an alternative and critical circuit for cardiomyocyte differentiation.

### *Gfra1/2* Are Required for Ventricular Compaction In Vivo

**Figure 6:**
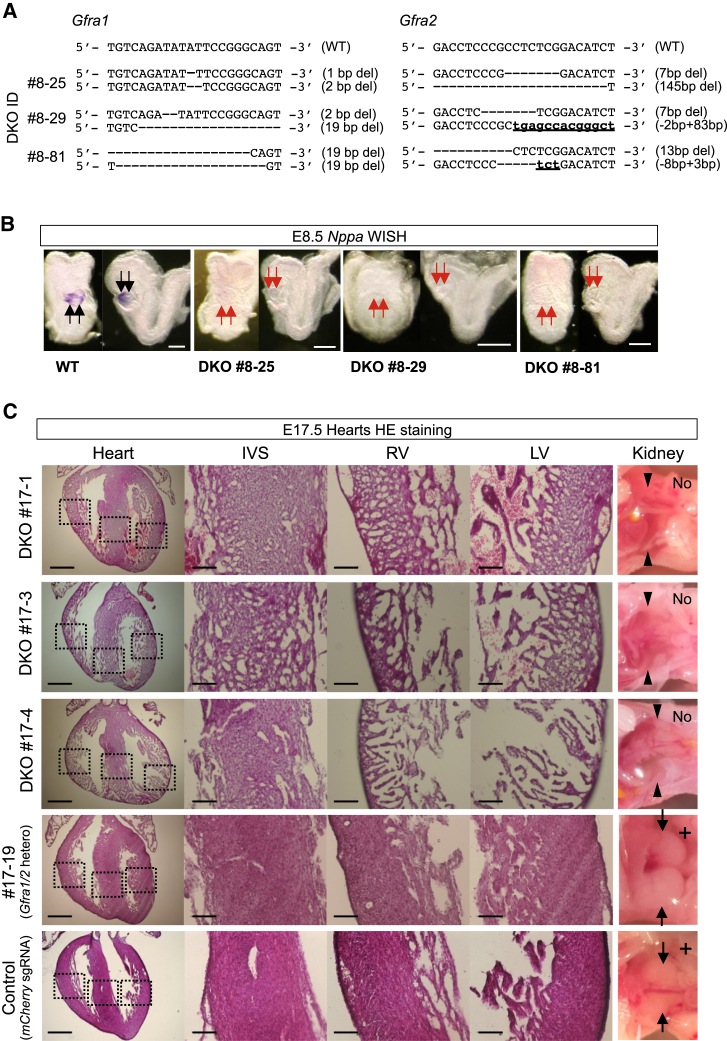
*Gfra1/2* Play Important Roles for the Heart Development In Vivo (A) Genotype of the *Gfra1/2* DKO mouse embryos generated by the direct injection of *Gfra1*-targeted sgRNA, *Gfra2*-targeted sgRNA, and *Cas9* mRNA into zygotes. (B) WISH analyses of differentiated cardiomyocyte marker *Nppa* in *Gfra1/2* DKO and WT littermate embryos at E8.5. *Nppa* expressions disappeared in DKO embryos (red arrows). Scale bar, 250 μm. (C) H&E staining for the hearts of *Gfra1/2* DKO E17.5 embryos. The compaction layers of myocytes were thin, and the alignments of cardiomyocytes were impaired in *Gfra1/2* DKO embryos as compared to the control hearts (*mCherry* sgRNA and Cas9 mRNA transduced embryos) and *Gfra1/2* compound heterozygote mutant. *Gfra1* null resulted in kidney agenesis as previously described (black arrowheads, No), whereas the well-developed kidney was observed in the heterozygotes and WT (black arrows, +) (Enomoto et al., 1998). The mutation of each embryo induced by CRISPR/Cas9 is shown in Figure S7B. IVS, intraventricular septum; RV, right ventricle; LV, left ventricle. Scale bar, 500 μm in whole-heart images and 100 μm in higher magnification. See also Figure S7.

**Figure 7:**
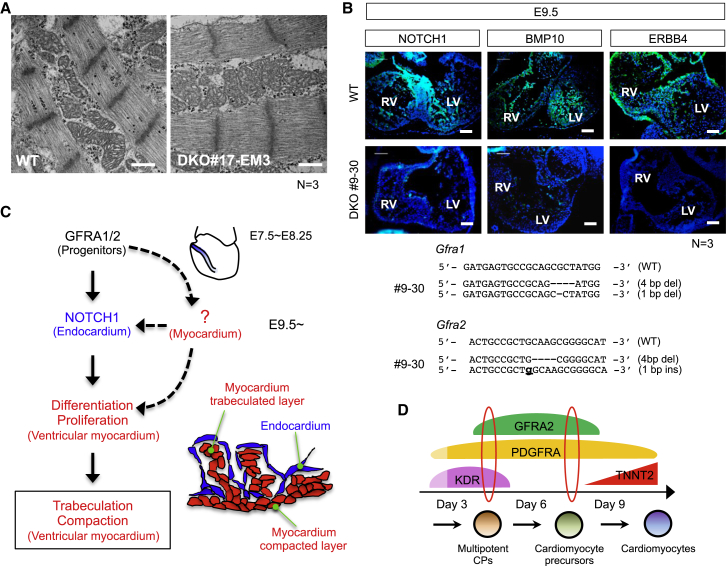
Impaired NOTCH Signaling in *Gfra1/2* DKO Embryos Is Responsible for Noncompaction Cardiomyopathy (A) The unaltered structure of sarcomeres and mitochondria in the hearts of *Gfra1/2* DKO embryos. The data represent biological triplicates. Scale bar, 500 nm. (B) Downregulation of NOTCH1, BMP10, and ERBB4 in E9.5 DKO hearts (green). The data represent biological triplicates. Blue, DAPI; LV, left ventricle; RV, right ventricle. (C) Schematic model of the in vivo function of non-canonical *Gfra1/2* signal pathway. (D) A model of the expression pattern of GFRA2 and other surface markers during differentiation of mouse and human pluripotent stem cells. At an earlier stage of differentiation, GFRA2^+^/KDR^low+^/PDGFRA^+^ marks multipotent cardiovascular progenitors, whereas GFRA2^+^/KDR^−^/PDGFRA^+^ marks cardiac precursors that are committed to cardiomyocytes fate at the later phase. See also Figure S7.

To exclude the possibility that the phenotype observed in *Gfra1/*2 DKO ESCs is an in vitro phenomenon, we generated *Gfra1/2* DKO mouse embryos by the direct transduction of (sgRNAs) for *Gfra1/2* and Cas9 mRNA into zygotes (Figures 6A–6C and S7A–S7D) (Wang et al., 2013). At first, we assayed the embryos at E8.5 just after heart tube formation, since embryonic defects or lethality would preclude the analysis of later stage embryos. Embryos containing multiples of three base insertions/deletions inside the exon were discarded from the analysis because the presence of functional protein production could not be refuted (Figure 6A). We examined cardiomyocyte differentiation of *Gfra1/2* DKO embryos by WISH using a marker of differentiated cardiomyocytes, *Nppa* (also known as *ANF*) (Bruneau et al., 2001, Christoffels et al., 2000). As expected from our ESC experiments, *Gfra1/2* DKO embryos exhibited a significant reduction of *Nppa* (Figure 6B), suggesting the cardiomyocyte differentiation process was significantly affected. As expected from the data of *Gfra2* KO ESCs (Figure 5D), *Gfra2* single-KO embryos showed significant but transiently elevated *Gfra1* expression by E8.5 in the heart field (Figure S7A). However, despite the fact that cardiomyocyte differentiation was impaired, the macro- and micro-anatomical morphology of the formed heart tube appeared unaffected in any DKO embryos (Figures 6B and S7B). This suggests that the reduction of *Nppa* in *Gfra1/2* DKO embryos simply reflected a delay of cardiomyocyte differentiation. To clarify this, we analyzed the *Gfra1/2* DKO embryos at E17.5 (Figures 6C, S7C, and S7D). Surprisingly, DKO embryos had the capacity to develop up to this stage without edema, and developed hearts were also observed (Figure 6C). This indicates that sufficient cardiomyocyte differentiation occurred in DKO embryos to support the fetal circulation. Further histological examination unexpectedly revealed that *Gfra1/2* DKO hearts at E17.5 suffered from noncompaction cardiomyopathy (Figures 6C and S7D). Excessively prominent trabeculations and deep intra-trabecular recesses, which are characteristic features of noncompaction cardiomyopathy, were apparent, but no other congenital heart disease in DKO embryos was observed. However, the structure of sarcomeres and mitochondria were not altered (Figures 7A and S7C), suggesting that noncompaction was not caused by the abnormality of sarcomeres and mitochondria (Towbin et al., 2015). The absence of *Gfra1/2* and *Nrtn* at the sites of trabeculation and compaction suggests that RET-dependent and -independent GFRA1/2 signal pathways do not directly or locally regulate trabeculation and compaction (Figures S7A, S7E, and S7F). Importantly, we found that NOTCH1 in total protein and its downstream molecules BMP10 and ERBB4 were missing in DKO hearts at E9.5 (Figure 7B). NOTCH signaling is essential for proliferation and differentiation of ventricular cardiomyocytes through which proper trabeculated and compacted myocardial layers are formed, and mutants with NOTCH signaling defects exhibit a noncompaction phenotype (de la Pompa and Epstein, 2012, Grego-Bessa et al., 2007, Luxán et al., 2013, Zhang et al., 2013). Thus, the ventricular noncompaction observed in DKO embryos likely resulted from altered NOTCH signaling (Figures 7B and 7C).

Taken together, these results suggest that GFRA1/2 plays an important function in normal mammalian heart development, especially for ventricular wall compaction, but an unknown mechanism could compensate for cardiomyocyte differentiation due to the lack of GFRA1/2 in vivo. Thus, taken together, our data reveal a non-canonical signal cascade via GFRA1/2 is indispensable for heart development in vivo.

## Discussion

Here, we report a surface marker, GFRA2, that is specific for CPs in mouse and human. We show that the expression of *Gfra2* is initiated among both FHF and SHF CPs in vivo and in vitro just before the initiation of spontaneous beating of cardiomyocytes. *Gfra2* is downregulated after CPs terminally differentiate to cardiomyocytes. The use of an antibody specific for GFRA2 protein made it possible for us to harvest human and mouse CPs derived from pluripotent stem cells. Physiologically, *Gfra2* plays an important role in heart development in vitro as well as in vivo, but, in the absence of *Gfra2*, ectopic activation of *Gfra1* can functionally compensate for its loss. Of note, our data suggest that an alternative non-canonical signaling cascade transmits GFRA1/2 activation to CP function, and that this is distinct from the canonical signaling pathway dependent on RET.

In differentiating ESCs, it is known that KDR^low+^/PDGFRA^+^ or KDR^low+^/KIT^neg^ cells constitute multipotent CPs which give rise to cardiomyocytes, smooth muscle cells, and endothelial cells, based on previously reported clonal tracing experiments (Bondue et al., 2011, Kattman et al., 2006, Kattman et al., 2011, Yang et al., 2008). However, it remains an open question as to whether all of these cells or only a proportion of these cells are CPs. In this study, we found that almost all mouse KDR^low+^/PDGFRA^+^ express GFRA2 (Figure 3). However, the situation is likely somewhat different in human. The proportion of hGFRA2-expressing cells among hKDR^low+^/hPDGFRA^+^ population is much lower (Figure 4F). As expected, hKDR^low+^/hPDGFRA^+^ cells are negative for KIT (Yang et al., 2008). Of note, most GFRA2-negative hKDR^low+^/hPDGFRA^+^ cells failed to differentiate to cardiac cells (Figure S3E), which strongly supports the specificity of hGFRA2 for human CPs. Thus, in humans, the additional usage of hGFRA2 labeling is superior to the previously proposed protocols to isolate multipotent CPs with high purity. Furthermore, the use of GFRA2 labeling in addition to KDR and PDGFRA will enrich for more mature unipotent CPs which cannot be isolated with previous protocols dependent on KDR expression. It is interesting that these unipotent late-stage GFRA2^+^ CPs represent not only the FHF, represented by HCN4/TBX5 and already known as unipotent, but also the SHF identified through expression of ISL1, because the SHF CPs are generally thought as multipotent (Figures 2G, 4E, S2D, and S2E) (Devine et al., 2014, Evans et al., 2010, Kelly and Evans, 2010, Kokkinopoulos et al., 2015, Lescroart et al., 2014, Später et al., 2013). This evidence indicates that the late-stage expression of GFRA2 excludes the multipotent SHF but includes the already committed but not fully differentiated SHF lineage. Thus, here we propose a strategy to isolate stage-specific human and mouse CPs with GFRA2, PDGFRA, and KDR (Figure 7D). GFRA2^+^/KDR^low+^/PDGFRA^+^ triple-positive CPs would be multipotent cardiovascular progenitors. As CPs begin to commit but not yet terminally differentiate to cardiomyocytes, they lose KDR expression so that a GFRA2^+^/KDR^neg^/PDGFRA^+^ double-positive population represents a cardiomyocyte precursor at a later stage.

We found that *Gfra1/2* DKO mouse ESCs showed a severe impairment of cardiomyocyte differentiation. Our data reveal that GFRA2 plays a pivotal role in cardiomyocyte differentiation in vitro that can be compensated by upregulation of GFRA1. Examples of an ectopic upregulation of an evolutionally duplicated paralogous gene to compensate for loss of a gene have been described (Barbaric et al., 2007). An evolutionally close relationship between *Gfra1* and *Gfra2* suggests that a preserved common enhancer drives *Gfra1* if *Gfra2* is not expressed in CPs, whereas a high level of *Gfra2* primarily suppresses *Gfra1* (Barbaric et al., 2007, Hätinen et al., 2007). However, the phenotype of KO of the known ligands for GFRA1 and 2, *Gdnf* and *Nrtn*, or KO of its co-receptor *Ret* did not show any defect of cardiomyocyte differentiation (Figures 5, S4, and S5). Interestingly, we observed a tendency of a slightly reduced yield of cardiomyocytes in single KO of *Gfra1*, *Gfra2*, and *Ret*, although not to a statistically significant degree (Figure 5B). Given the evidence that previously reported KO mice of each gene also did not show any heart defect (Enomoto et al., 1998, Heuckeroth et al., 1999, Rossi et al., 1999, Sánchez et al., 1996), the role of classic RET-dependent GFRA1/2 signals are unlikely to be vital for heart development. In addition, whereas an alternative GFRA1 signal pathway via NCAM1/FYN/FAK is known (Paratcha et al., 2003, Sjöstrand et al., 2007), our data showed that NCAM1, FYN, and FAK were not involved in the cardiac differentiation defect in the *Gfra1/2* DKO (Figure S6). Thus, the pathway by which GFRA2 modulates heart development is likely to be distinct from previously established pathways. To uncover the nature of this alternative non-canonical signaling pathway acting via GFRA1/2, further investigation is required, to identify molecules interacting directly with GFRA2 in the context of cardiac differentiation.

In contrast to the in vitro phenotype, the loss-of-function of *Gfra1/2* showed a different phenotype in vivo. In E8.5 DKO embryos, *Nppa* expression disappeared, which is likely consistent with the impaired in vitro cardiomyocyte differentiation of DKO ESCs. However, viable E17.5 DKO embryos possessing a developed heart were observed, indicating that cardiomyocyte differentiation itself occurred to form a functional fetal heart in vivo. Indeed, although missing *Nppa*, the heart tube of E8.5 DKO embryos seemed morphologically normal (Figures 6B and S7B). This evidence is inconsistent with the in vitro phenotype of ablated cardiomyocyte differentiation in DKO ESCs. Thus, the disappearance of *Nppa* in E8.5 DKO embryos might simply represent the delay of cardiomyocyte differentiation or anomalous sequential events inside the cardiomyocytes in DKO embryos. We propose that an unknown compensatory mechanism functions in vivo to circumvent the lack of GFRA1/2 to promote cardiomyocyte differentiation. Although the differentiation protocol for ESCs used in this study provides a strong drive toward cardiomyocytes with a defined set of growth factors (Kattman et al., 2011, Wamstad et al., 2012), these conditions are probably insufficient to perfectly mimic the in vivo environment for cardiomyocyte differentiation.

E17.5 DKO embryos showed noncompaction cardiomyopathy without other congenital heart diseases (Towbin et al., 2015, Zhang et al., 2013). We speculate that these DKO mice would not survive after birth because of the extremely thin compact layer of the ventricular wall. However, absence of edema in E17.5 DKO embryos suggests that the contractile apparatus in DKO cardiomyocytes at least developed sufficiently to support the fetal circulation. Unfortunately, germline deletion of *Gfra1* does not allow neonates to survive due to kidney agenesis (Figure 6C) (Enomoto et al., 1998, Sánchez et al., 1996). Thus, to confirm the perinatal prognosis, a conditional knockout will be required.

Importantly, we found that NOTCH1 and its downstream targets, BMP10 and ERBB4, were significantly downregulated in DKO embryos, and that this might be responsible for the noncompaction defect (Figures 7B and 7C) (Grego-Bessa et al., 2007, Luxán et al., 2013, Zhang et al., 2013). Given the lack of *Nppa* expression in NOTCH signal mutants (Luxán et al., 2013), it is concluded that the altered NOTCH signal in the endocardium promotes the downregulation of *Nppa* in DKO embryos. Surprisingly, *Gfra2* is expressed by CPs, but not at the sites of trabeculation or compaction (Figures 1, S1, S7E, and S7F). Thus, the defect within the endocardium is cell autonomously induced by the loss of *Gfra1/2* in multipotent CPs and is likely primarily required for ventricular noncompaction, although we cannot exclude a possibility that additional defects within the myocardium of DKO embryos may also contribute to this phenotype (Figure 7C).

Taken together, an alternative signaling pathway via GFRA1/2 is indispensable for proper heart development, although the mechanism underlying the signaling pathway of cardiac differentiation mediated by GFRA2 has yet to be elucidated. Future work, involving the clarification of the mechanistic details of this signaling pathway should provide deeper insight into cardiomyocyte differentiation, the biology of CPs, normal trabeculation, and compaction of ventricular myocytes.

## Experimental Procedures

The details are given in the Supplemental Experimental Procedures.

### Animals

All animal procedures in this project were carried out under the project licenses 70/7254 and 80/2452 and 27-028-001 approved by the Home Office according to the Animals (Scientific Procedures) Act 1986 in the UK and Osaka University Animal Ethical Committee in Japan, respectively.

### Cell Culture and Differentiation

Cardiomyocyte induction for mouse E14tg2a ESCs (Magin et al., 1992, Smith and Hooper, 1987) was performed according to standard protocols as previously described (Kattman et al., 2011). Cardiomyocyte induction from human HUES7 ESCs or iPSCs was undertaken as previously described (Burridge et al., 2011). Human ESCs were used under the license of the UK Steering Committee (reference number; SCSC13-25). Human iPSCs (iPS-HS1M) were originally established by D.M. using human dermal fibroblasts (HDFs) from a healthy donor under informed consent (Health Research Authority approval 13/LO/0224), for a study to be described elsewhere (D.M., T. McKay, L.D., and A.T., unpublished data).

### CRISPR/Cas9-Mediated Genome Editing

The CRISPR/Cas9-mediated genome editing for mouse ESCs and embryos was performed as previously described (Hashimoto and Takemoto, 2015, Wang et al., 2013).

### Flow Cytometry/FACS

Cells were sorted as previously described using FACS ARIA II or analyzed by LSR Fortessa II or FACSConto II (BD Biosciences) with FACSDiva 7.0 software (Kokkinopoulos et al., 2015).

## Author Contributions

K.Y. planned this project, and H.I. and K.Y. performed the majority of the experiments. K.Y. and H.I. wrote the manuscript. R.S., I.K., S.N., A-K.H., M.S., P.R., and K.O. contributed to mouse ESC experiments. A.P., D.M., S.H., L.D., A.T., and K.O. contributed to human ESCs and human iPSCs experiments. M.H. and H.S. contributed to CRISPR/Cas9-mediated direct genome editing. O.Y. and Y. Sakata contributed to electron microscopy observations. S.K., A-K.H., Y. Sawa, K.O., and K.S. contributed to the discussion of the results.

## Supplemental Information

Document S1. Supplemental Experimental Procedures and Figures S1–S7

Movie S1. Spontaneously Beating Cardiomyocytes Differentiated from GFRA2^+^/PDGFR-α^+^ Mouse Cardiac Progenitors after FACS Isolation, Related to Figure 2 After an additional 5 days culture in differentiation media, GFRA2^+^/PDGFR-α^+^ cells showed synchronized spontaneous contraction, suggesting that they can effectively differentiate into functional cardiomyocytes.

Movie S2. Spontaneously Beating Cardiomyocytes Derived from FACS-Isolated hGFRA2^+^/hPDGFR-α^+^ Human Cardiac Progenitors, Related to Figure 4 FACS-isolated hGFRA2^+^/hPDGFR-α^+^ cells from human ESCs differentiated into spontaneously beating cardiomyocytes.

Document S2. Article plus Supplemental Information
