# GFRA2 Identifies Cardiac Progenitors and Mediates Cardiomyocyte Differentiation in a RET-Independent Signaling Pathway

**DOI:** 10.1016/j.celrep.2023.113383

**Published:** 2023-10-25

**Authors:** Hidekazu Ishida, Rie Saba, Ioannis Kokkinopoulos, Masakazu Hashimoto, Osamu Yamaguchi, Sonja Nowotschin, Manabu Shiraishi, Prashant Ruchaya, Duncan Miller, Stephen Harmer, Ariel Poliandri, Shigetoyo Kogaki, Yasushi Sakata, Leo Dunkel, Andrew Tinker, Anna-Katerina Hadjantonakis, Yoshiki Sawa, Hiroshi Sasaki, Keiichi Ozono, Ken Suzuki, Kenta Yashiro

(Cell Reports *16*, 1026–1038; July 26, 2016)

In the originally published version of this article, an inadvertent error appeared in Figure 5G wherein images of the same specimen were mistakenly presented as Gdnf-KO #2 and Nrtn-KO #13. After careful investigation by the authors, it has been determined that the specimens shown are identical, albeit with a slight difference in the region of interest. The authors sincerely regret this oversight and would like to emphasize that it was a genuine error and in no way intentional. The authors have carefully reviewed their stored data and experimental notebooks, and in the process, it was found that the image of Gdnf-KO #2 had been used incorrectly. Due to the age of the article, the original figure cannot be replaced, but the revised figure is shown below for readers. This revised Figure 5 replaces the original, incorrect image of Gdnf-KO #2 with the correct one. The authors would like to assure readers that this error does not affect the conclusions of the study. They deeply regret any confusion this may have caused and appreciate the understanding of the scientific community.

**Figure 5:**
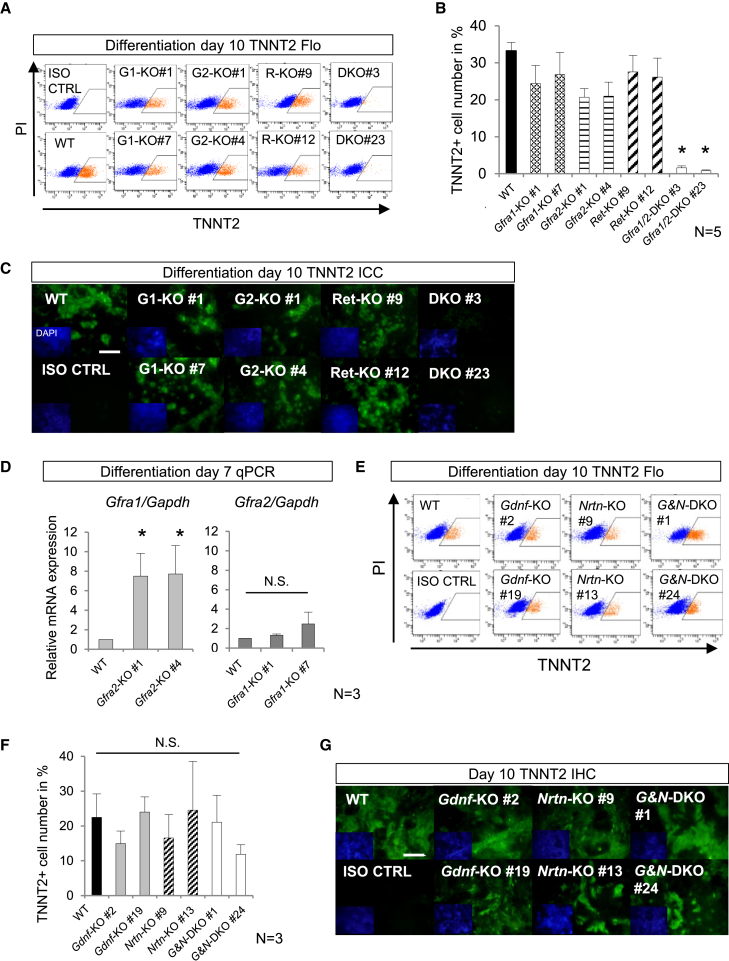
*Gfra1* and *Gfra2* Are Essential for In Vitro Cardiomyocyte Differentiation from Mouse ESCs (corrected)

