# Supplementary material for: GFRA2 Identifies Cardiac Progenitors and Mediates Cardiomyocyte Differentiation in a RET-Independent Signaling Pathway
Source: Cell Rep. 2016 Jul 7;16(4):1026–38. doi: 10.1016/j.celrep.2016.06.050 (PMC4967477; doi:10.1016/j.celrep.2016.06.050)
Supplement: Document S1. Supplemental Experimental Procedures and Figures S1–S7 [file mmc1.pdf]

Cell Reports, Volume *16*

Supplemental Information

GFRA2 Identifies Cardiac Progenitors and Mediates Cardiomyocyte Differentiation in a RET-Independent Signaling Pathway

Hidekazu Ishida, Rie Saba, Ioannis Kokkinopoulos, Masakazu Hashimoto, Osamu Yamaguchi, Sonja Nowotschin, Manabu Shiraishi, Prashant Ruchaya, Duncan Miller, Stephen Harmer, Ariel Poliandri, Shigetoyo Kogaki, Yasushi Sakata, Leo Dunkel, Andrew Tinker, Anna-Katerina Hadjantonakis, Yoshiki Sawa, Hiroshi Sasaki, Keiichi Ozono, Ken Suzuki, and Kenta Yashiro

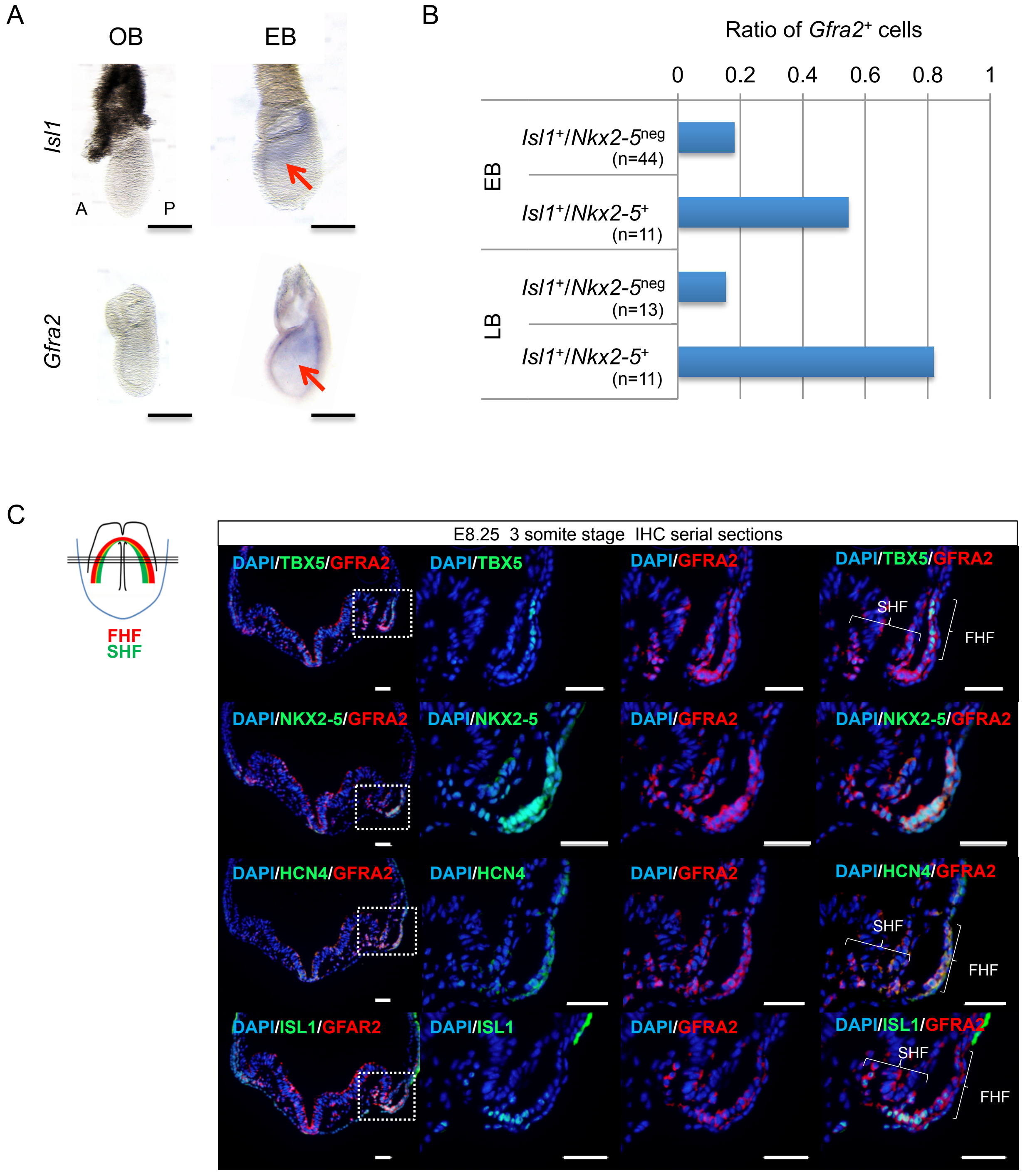

Figure S1. Related to Figure 1. **The distribution of GFRA2 expression in the mouse embryo.**
(A) Whole mount in situ hybridization (WISH) for *Isl1* and *Gfra2* on No Bud (OB) stage and Early allantoic Bud (EB) stage embryos. The embryos are indicated as the left lateral view. The data are representative of biological duplicates and triplicates for OB stage and EB stage, respectively. For *Gfra2* at the EB stage, the indicated data is the same as in Fig. 1A. Note both genes were clearly visible at the EB stage but not at the OB stage. A; anterior, P; posterior, Scale bar; 250 µm. (B) The ratio of *Gfra2*$^{+}$ single cell cDNAs among those of *Isl1*$^{+}$/*Nkx2-5*^{neg} and of *Isl1*$^{+}$/*Nkx2-5*$^{+}$. LB; Late allantoic Bud stage. (C) Immunohistochemistry of GFRA2, TBX5, NKX2-5, ISL1, and HCN4 on serial sections of the three-somite stage embryo. GFRA2^{+} cells are NKX2-5^{+} CPs and the sum of TBX5^{+}/HCN4^{+} FHF and ISL1^{+} SHF CPs. FHF, first heart field; SHF, second heart field. Scale bar; 50µm. Micrograph represents biological triplicates.

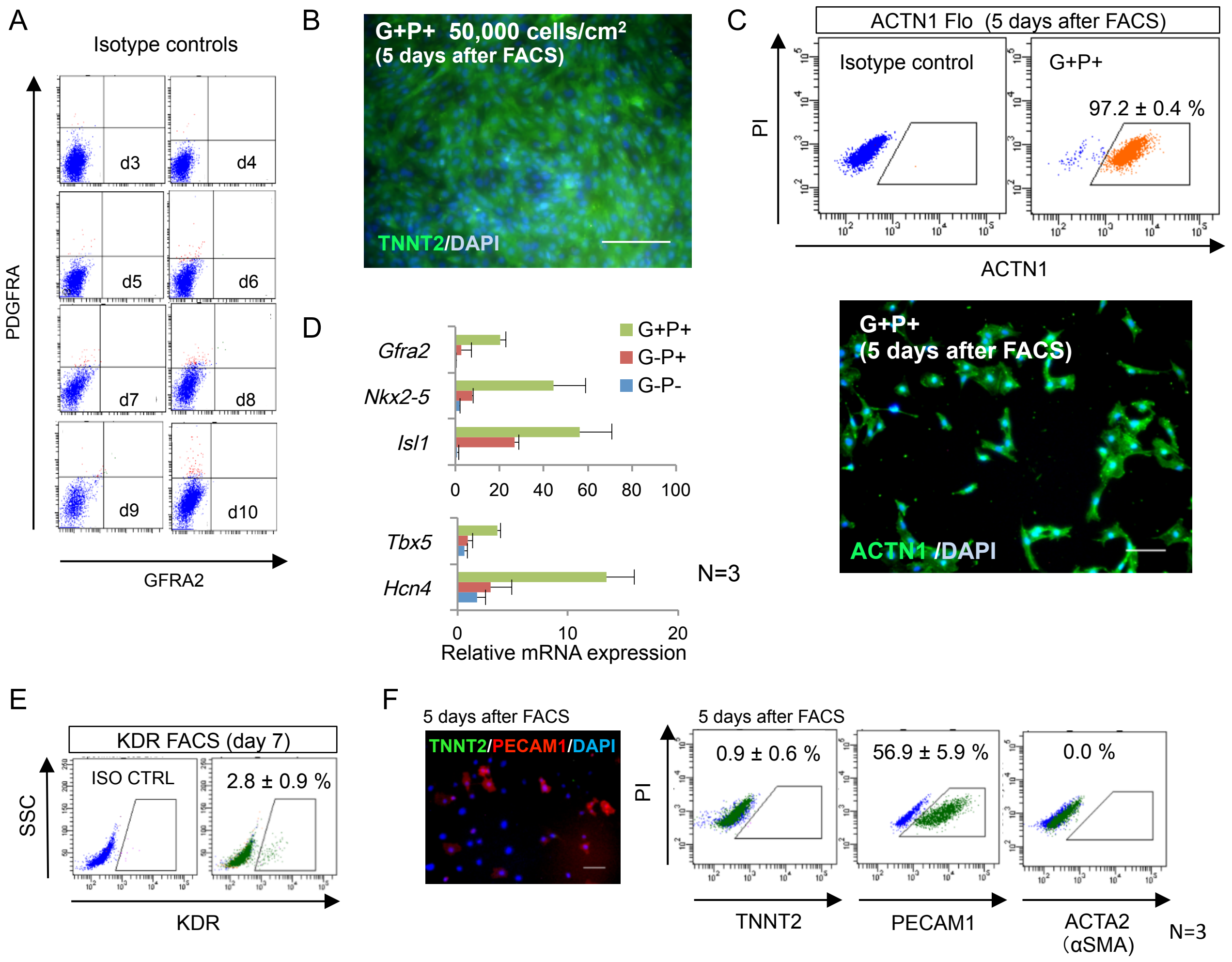

Figure S2. Related to Figure 2 and 3. **Biological features of GFRA2$^+$ CPs from mouse ESCs.**
(A) Isotype controls of serial differentiation stages of mouse ESCs for Figure 2A. (B) Immunofluorescence of TNNT2 in FACS-isolated GFRA2$^+$/PDGFRA$^+$ (G+P+) cells 5 days after FACS. Cells were seeded at higher density (50,000 cells/cm^2). Note almost all cells show TNNT2 positive and spontaneous synchronized beating (See Movie S1). Cells could expand more efficiently when seeded at higher density. Scale bar; 100 µm. (C) Immunocytochemistry and flow cytometry (Flo) for another cardiomyocyte marker ACTN1 (also known as ACTININ α1). GFRA2$^+$/PDGFRA$^+$ cells were isolated by FACS at day 7 and differentiated for a further 5 days. Most of the cells (97.2 ± 0.4%) were ACTN1 positive cardiomyocytes. N=3. (D) qPCR analyses of *Gfra2*, *Nkx2-5*, *Isl1*, *Tbx5*, and *Hcn4* in FACS-isolated G+P+, G–P+, G–P– cells at day 7. Expression levels of all cardiac genes were significantly higher in the G+P+ population when compared to G–P+ and G–P– population (*$P < 0.05$, Student t-test). Data are representative of biological triplicates with technical duplicates as mean ± s.e.m.. (E) Flow cytometrical analysis of KDR in day 7 differentiating ESCs. (F) Differentiation fate of FACS-purified KDR$^+$ cells. KDR$^+$ cells were isolated at day 7 and kept in culture for 5 days. Immunocytochemical and flow cytometrical analyses show KDR$^+$ cells at day 7 efficiently differentiated into endothelial cells. Scale bar; 100 µm.

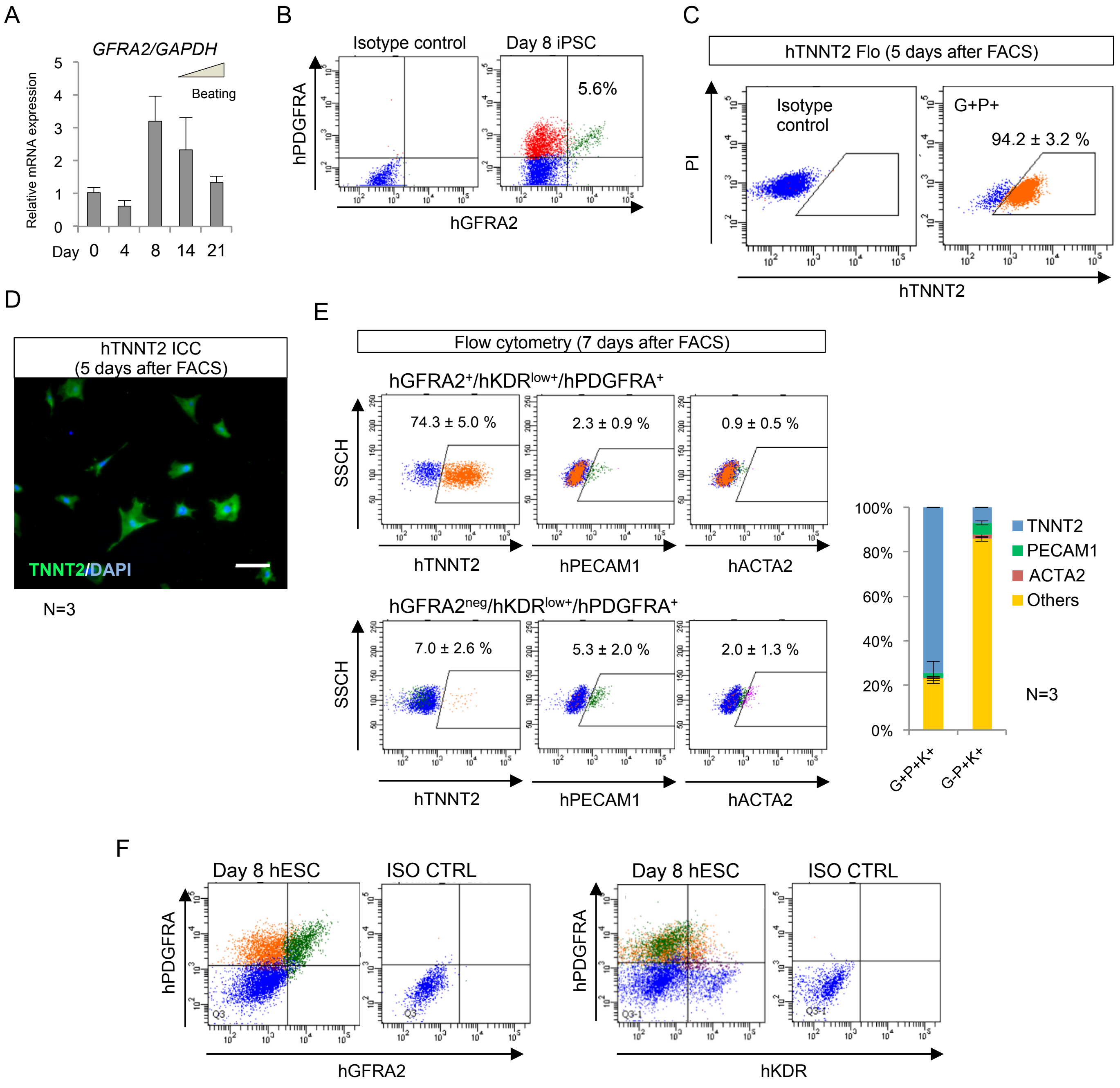

Figure S3. Related to Figure 4. **hGFRA2$^+$ CPs derived from human iPSCs.**
(A) qPCR analysis for human *GFRA2* during cardiac differentiation of human iPSCs. A transient expression peak of human *GFRA2* was observed at day 8, similar to mouse and human ESCs. Bar graph represents biological triplicates with technical duplicates as mean ± s.e.m.. (B) Flow cytometrical analysis of hGFRA2 and hPDGFRA on differentiation day 8 human iPSCs. Similar to mouse and human ESCs, hGFRA2$^+$/hPDGFRA$^+$ CP population could be clearly seen in cardiac differentiating iPSCs. (C, D) FACS-isolated hGFRA2$^+$/hPDGFRA$^+$ CPs from human iPSCs efficiently differentiated into TNNT2$^+$ cardiomyocytes. Scale bar; 100 µm. (E) Flow cytometrical analyses for hTNNT2 (cardiomyocytes), hPECAM1 (endothelial cells), and hACTA2 (smooth muscle cells; also known as αSMA) 7 days after the FACS-isolation of hGFRA2$^+$/hKDR^{low+}/hPDGFRA$^+$/hKIT^{neg} and hGFRA2^{neg}/hKDR^{low+}/hPDGFRA$^+$/hKIT^{neg} population. Note hGFRA2 negative population almost lost cardiac differentiation ability. Bar graph represents mean ± s.e.m. N=3. (F) Flow cytometry for hGFRA2, hPDGFRA, and hKDR at day 8 of human ESCs. hGFRA2$^+$/hPDGFRA$^+$ CPs (shown as green dots) lost hKDR expression (right column) as compared with day 4 (Fig. 4f).

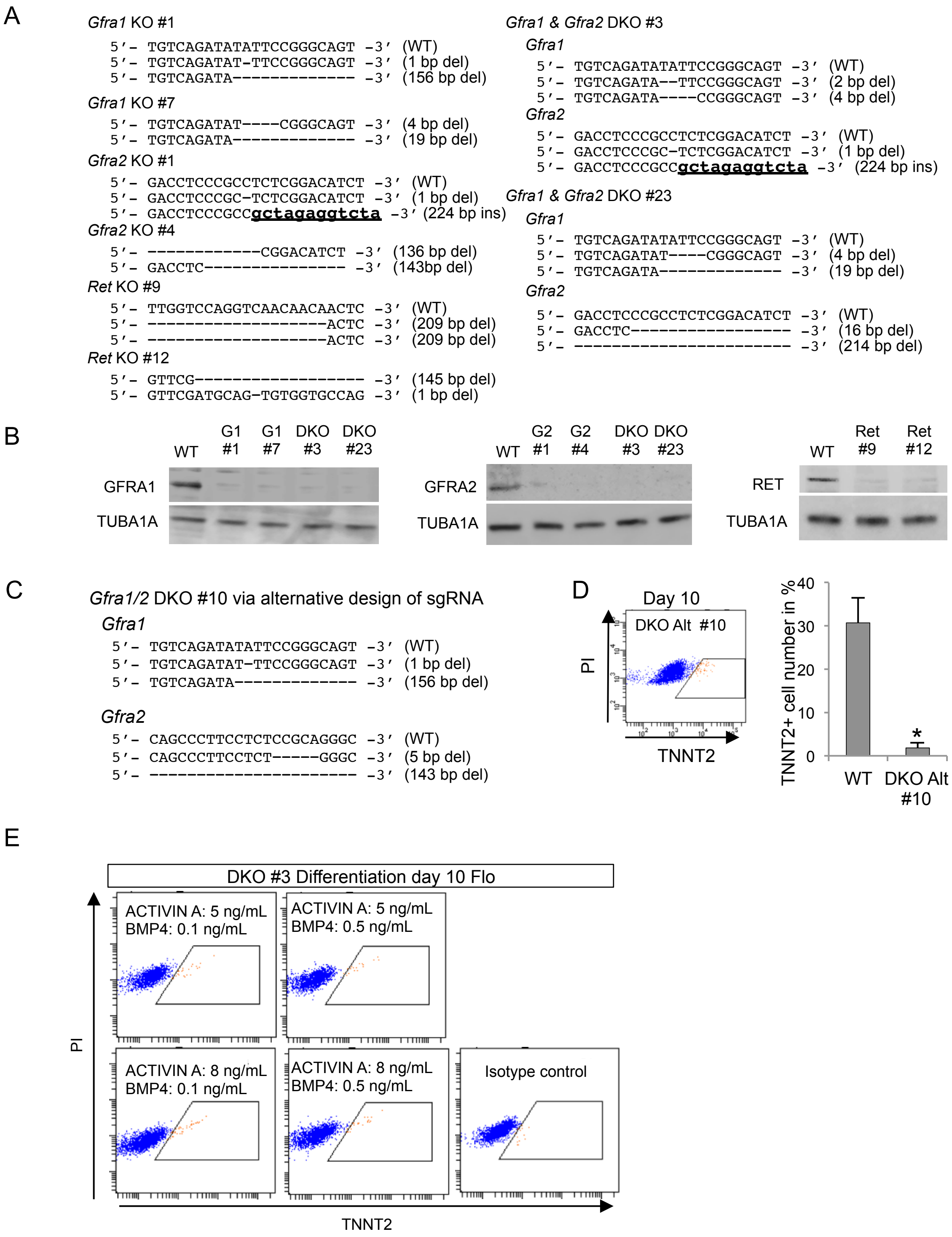

Figure S4. Related to Figure 5. ***Gfra1, Gfra2, Ret*, and *Gfra1/2* knockout mouse ES cell clones.**
(A) Genotyping results of each knockout (KO) ES cell line. In principle, all mutations cause premature termination of translation of proteins. (B) Immunoblotting images of each KO ES cell line revealing them to be protein nulls. (C) The genotype of DKO clone ID_#10 via another different design of sgRNA for *Gfra2*. Targeting was performed on *Gfra1* KO clone ID_#1. (D) An alternative DKO clone ID_#10 also exhibited a severe impairment in cardiomyocyte differentiation, suggesting that this cardiac defect is not caused by off-target mutations. (E) Flow cyometrical analyses for TNNT2 in differentiation day 10 indicates that the cardiac differentiation defect of DKO #3 ESCs was not improved by varied concentration of ACTIVIN A and BMP4.

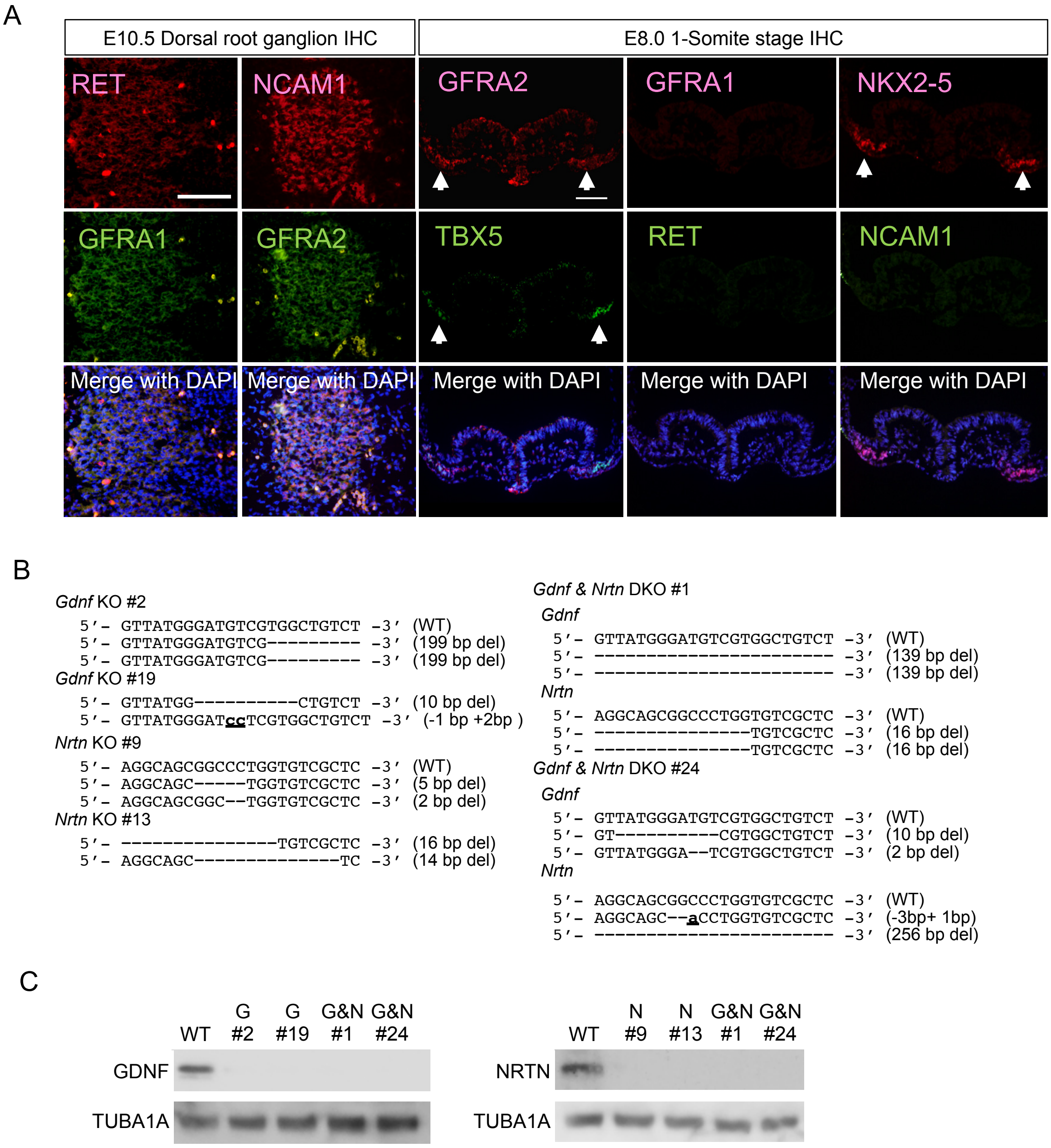

Figure S5. Related Figure 5. **Mouse ES cell clones of *Gdnf* KO, *Nrtn* KO, and *Gdnf/Nrtn* DKO.**
(A) Micrograph represents the reproduced immunofluorescence staining of GFRA1, GFRA2, RET, NKX2-5, TBX5, and NCAM1 from the 3-somite stage embryos (N=3). Note GFRA1, RET, and NCAM1 were not detected. White arrows indicate the heart field. The validity of used antibodies against GFRA1, RET, and NCAM1 is shown in the dorsal root ganglion of E10.5 mouse embryos. Scale bar; 100 µm. (B) Genotyping results for each *Gdnf*, *Nrtn,* and *Gdnf/Nrtn* KO ES cell line. All mutations caused premature termination of translation of proteins. (C) Immunoblotting images of each KO ES cell line confirmed them as protein nulls. G, *Gdnf*-KO. N, *Nrtn*-KO. G&N, *Gdnf/Nrtn*-DKO.

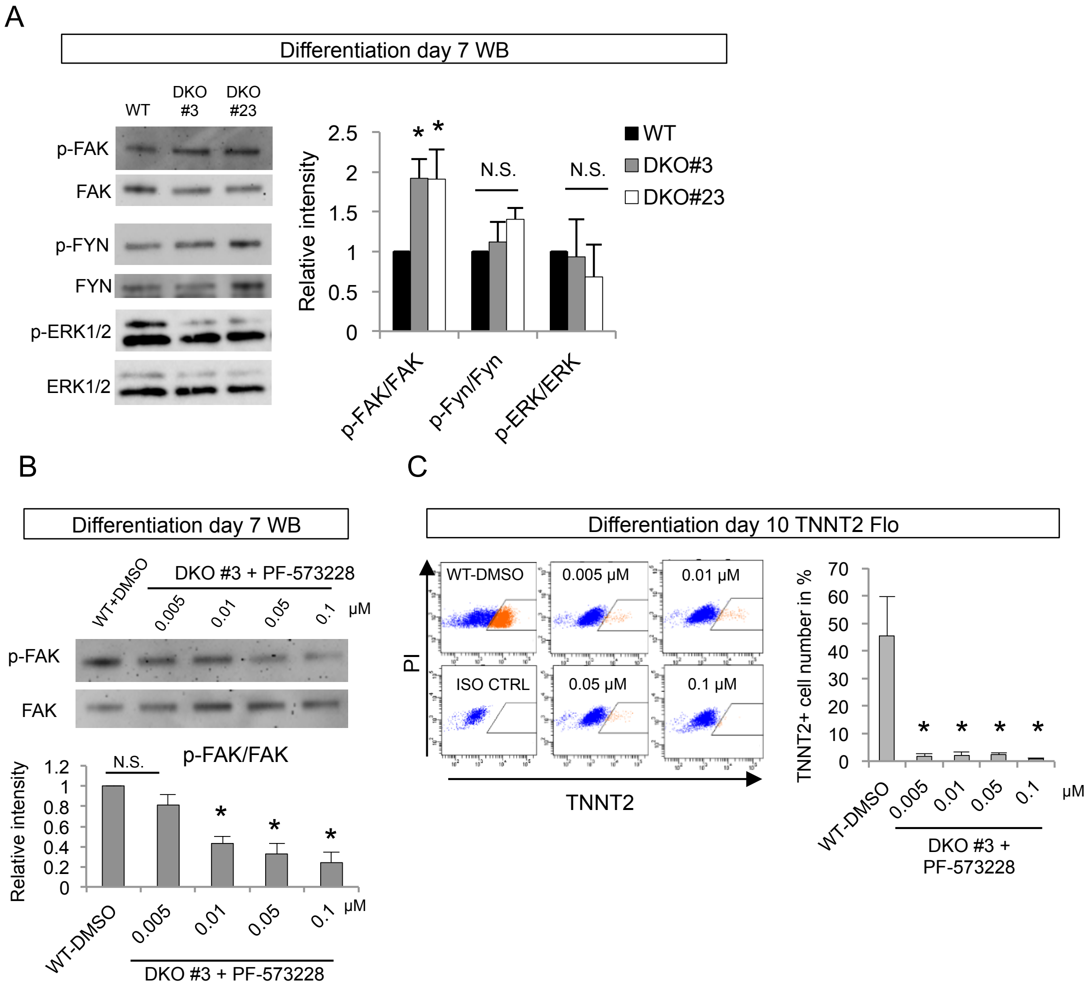

Figure S6. Related to Figure 5. **Signal pathway analyses in *Gfra1/2* DKO ESCs.**
(A) Western blotting (WB) analyses of phosphorylated FAK (p-FAK), FYN (p-FYN), and ERK1/2 (p-ERK1/2) at day 7 of differentiation in WT and DKO ESCs. p-FAK was significantly elevated in *Gfra1/2* DKO ES cell lines, whereas p-FYN and p-ERK1/2 were not significantly affected. *$P < 0.05$ vs WT in *Student's* t-test. N.S., not significant. Bar graph represents mean ± s.e.m.. N=3. (B) Serial concentrations of FAK inhibitor PF-573228 were administered to differentiation media from day 5. Note that p-FAK in DKO ESCs was not significantly different from WT when 0.005 μM. *$P < 0.05$ vs WT in *Student's* t-test. Bar graph represents mean ± s.e.m.. N=3. (C) Suppression of FAK phosphorylation failed to rescue cardiomyocyte differentiation defects in *Gfra1/2* DKO ESCs. *$P < 0.05$ vs WT in *Student's* t-test. Bar graph represents mean ± s.e.m.. N=3.

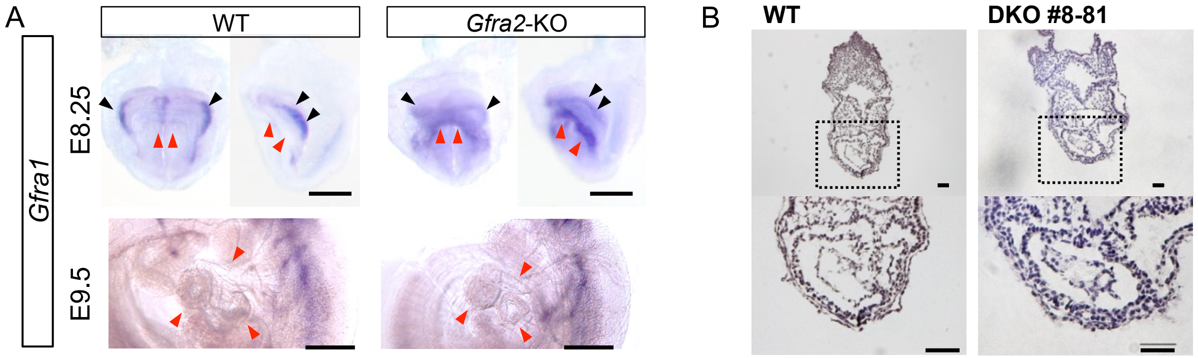

C

DKO ID	Gfra1	Gfra2
	5′- GATGAGTGCCGCAGCGCTATGG -3′ (WT)	5′- ACTGCCGCTGCAAGCGGGGCAT -3′ (WT)
#17-1	5′- -----------------TATGG -3′ (20 bp del)<br>5′- GATGAGTGCCGC-----TATGG -3′ (5 bp del)	5′- -----------------gtCAT -3′ (220 -> 2bp)<br>5′- -----------------gtCAT -3′ (220 -> 2bp)
#17-3	5′- GATGAGTGCCGC-----TATGG -3′ (5 bp del)<br>5′- GATGAGTGCCGC-----TATGG -3′ (5 bp del)	5′- ACTGCCGC-------GGGGCAT -3′ (7bp del)<br>5′- ACTGCCGCTGCcAAGCGGGGCAT -3′ (1 bp ins)
#17-4	5′- GATGAGTGCC-------TATGG -3′ (7 bp del)<br>5′- GATGAGTGCC-------TATGG -3′ (7 bp del)	5′- TCCTCTCCGCAGGGCTCTGAGC -3′ (WT)<br>5′- TCCTCTC----------TGAGC -3′ (10 bp del)<br>5′- TCCTCT-----------TGAGC -3′ (11 bp del)
#17-19 (Hetero)	5′- GATGAGTGCCGCAGCGCTATGG -3′ (WT)<br>5′- ---------------------- -3′ (176 bp del)	5′- ACTGCCGCTGCAAGCGGGGCAT -3′ (WT)<br>5′- ACTGCCGC-------GGGGCAT -3′ (7 bp del)
#17-EM3	5′- GATGAGTGCCGC-----TATGG -3′ (5 bp del)<br>5′- GATGAGTGCCGC-----TATGG -3′ (5 bp del)	5′- ACTGCCGC-------GGGGCAT -3′ (7bp del)<br>5′- ACTGCCGCTG----CGGGGCAT -3′ (4 bp del)

D

Genotype	Heart phenotype	Number
Gfra1 -/-	Normal	2*
	Noncompaction	0
Gfra2 -/-	Normal	2
	Noncompaction	0
Gfra1 +/-, *Gfra2* +/-	Normal	2
	Noncompaction	0
Gfra1 -/-, *Gfra2* +/-	Normal	4*
	Noncompaction	0
Gfra1 -/-, *Gfra2* -/-	Normal	0
	Noncompaction	3*

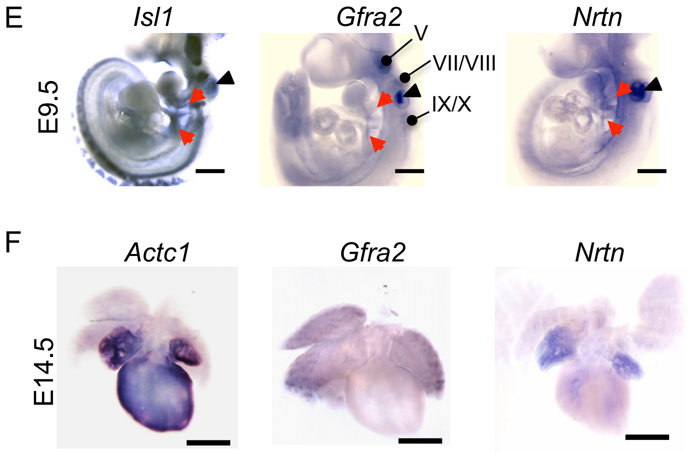

Figure S7. Related to Figure 6 and 7. **The phenotype of *Gfra1/2* DKO mouse embryos generated via CRISPR/Cas9 direct genome editing.**
(A) Transient ectopic *Gfra1* expression in *Gfra2* knockout embryos. *Gfra1* was expressed in the neural crest lineage (black arrowheads), but not in cardiac crescent (red arrowheads in the upper row) of WT embryo. By contrast, *Gfra1* was up-regulated in *Gfra2*-null embryos in the cardiac crescent at E8.25 (red arrowheads in the upper row of the right column) but not at the the heart region of E9.5 embryo (red arrowheads in the lower row). The heart of E9.5 is indicated as the left lateral view. Scale bar, 250 µm. (B) HE staining of the sections of E8.5 *Gfra1/2* DKO #81 mouse embryo and WT littermate. The morphological alteration of the heart tube was not noticed in DKO embryos, although *Nppa* disappeared (Fig. 6B). Scale bar; 50 µm. (C) Genotype of the E17.5 *Gfra1/2* DKO mouse embryos generated by CRISPR/Cas9 mediated direct genome editing. (D) Summary of the E17.5 mutants obtained via direct genome editing with CRISPR/Cas9. All the cases of *Gfra1* null showed kidney agenesis (asterisks). Note that only DKO embryos showed noncompaction. (E) WISH for *Isl1*, *Gfra2*, and *Nrtn* on WT mouse embryos at E9.5 when trabeculation of myocardium occurs. Note *Gfra2* and *Nrtn* were not expressed inside the heart, although we could not exclude that they were expressed in a part of SHF at the dorsal mesentery (red arrowheads) represented by *Isl1*. *Gfra2* and *Nrtn* was detected weakly at the pharyngeal pouch. The signal at otic vesicle is likely artifact (black arrowheads). The embryos are indicated as left lateral view. N=3. V; trigeminal nucleus, VII/VIII; facial/acoustic nucleus, IX/X; glossopharyngeal/vagus nucleus, Scale bar; 250 µm. (F) WISH for *Actc1* (a cardiomyocyte marker), *Gfra2*, and *Nrtn* on WT mouse embryonic hearts at E14.5 when myocardial compaction occurs. *Gfra2* was not expressed in the heart at E14.5 but was detected at alveoli in the lung. *Nrtn* was observed in the atrium and at the ventricular endocardium, but not in the ventricular myocardium layer. N=3. Scale bar; 750 µm.

Figure legends for Supplemental Movies

Movie S1. Related to Figure 2. **Spontaneously beating cardiomyocytes differentiated from $GFRA2^+/PDGFRA^+$ mouse cardiac progenitors after FACS isolation.**

After an additional 5 days culture in differentiation media, $GFRA2^+/PDGFRA^+$ cells showed synchronized spontaneous contraction, suggesting that they can effectively differentiate into functional cardiomyocytes.

Movie S2. Related to Figure 4. **Spontaneously beating cardiomyocytes derived from FACS-isolated $hGFRA2^+/hPDGFRA^+$ human cardiac progenitors.**

FACS-isolated $hGFRA2^+/hPDGFRA^+$ cells from human ESCs differentiated into spontaneously beating cardiomyocytes.

SUPPLEMENTAL EXPERIMENTAL PROCEDURES

Animals

All animal procedures in this project were carried out under the project licenses (70/7254 and 80/2452) and (27-028-001) approved by the Home Office according to the Animals (Scientific Procedures) Act 1986 in the UK and Osaka University Animal Ethical Committee in Japan, respectively.

Staging of mouse embryos

Developmental stages of mouse embryos were classified according to morphology as previously described (Downs and Davies, 1993). The morning of the day of vaginal plug detection was set as E0.5.

Single cell RNA-seq of embryonic cardiac progenitors

Deep sequencing and bioinformatics analyses on single-cell cDNAs were performed as previously described (Brouilette et al., 2012; Kokkinopoulos et al., 2015; Kurimoto et al., 2006). Sequences used to identify *Gfra2* have been submitted to NCBI Gene Expression Omnibus (GEO, http://www.ncbi.nlm.nih.gov/geo) under the accession numbers GSE38198 and GSE63796.

Whole mount in situ hybridization

All whole mount in situ hybridization procedures were performed as previously described (Uehara et al., 2009). A probe for *Gfra2* was kindly given by J. Cobb, D. Duboule and J. Nishino, for *Gfra1* by H. Hamada and J. Nishino, *Actc1* for R. Kelly and for *Nppa* by P. Riley. For *Nrtn*, cDNA clone of MGC:68236/IMAGE:5345262 was used for probe synthesis. Images were acquired with a Leica M205FA stereomicroscope using a DFC310 FX digital camera, or with Olympus SZX12 stereomicroscope using a DP70 digital camera.

Histology

Immunofluorescence on tissue sections were performed as previously described (Kokkinopoulos et al., 2015). Embryos were dissected and fixed with 4% paraformaldehyde (PFA) at 4 °C overnight. Then they were embedded in OCT compound and cryosections were prepared at 5 μm thickness. For immunohistochemistry, non-specific antibody binding sites were pre-blocked with 5% skimmed milk/PBS or 3% bovine serum albumin (BSA)/PBS for 30 min at room temperature. Primary antibodies used were; anti-ACTN1 (Sigma A7811, 1:200), anti-BMP10 (R&D MAB6038, 1:100), anti-ERBB4 (Santa Cruz, sc-284, 1:100), anti-GFRA2 (R&D AF429, 5 μg/mL), anti-HCN4 (Millipore AB5808, 1:100), anti-ISL1 (DSHB 39.4D5, 1:100), anti-NCAM1 (Abcam ab133345, 1:100), anti-NKX2-5 (Abcam ab35842, 1:100), anti-NOTCH1 (Cell Signaling Technology 4380, 1:100), and anti-TBX5 (SIGMA HPA8786, 1:100). Antigen retrieval was performed for the detection of NOTCH1 and BMP10 before primary antibody reaction, by incubation in 10 mM Citrate (pH 6.0) at a sub-boiling temperature for 10 min. To dilute a primary antibody, Can Get Signal Immunostain solution B (TOYOBO NKB-601) was used only for ERBB4. For secondary antibodies (all from Molecular Probes), donkey anti-goat Alexa 488 or 594 (1:400) and donkey anti-rabbit Alexa 488 (1:400) or donkey anti-mouse Alexa 488 (1:400) diluted with 5% skimmed milk/PBS or 3% BSA/PBS were applied for 90 min at room temperature. These secondary antibodies were also used for the immunocytochemistry. Nuclei were stained with 4',6-diamino-2-pheylindole (DAPI). The hearts of double knockout embryos were fixed with 4% PFA overnight and immersed in 30% Sucrose/PBS overnight. The sections were prepared at 10 μm thickness and stained with haematoxylin and eosin. Images were acquired with KEYENCE BZ8000 fluorescence or Zeiss LSM510 laser confocal microscopes.

Cell culture and differentiation

E14tg2a mouse ESCs (Magin et al., 1992; Smith and Hooper, 1987) were maintained under feeder-free conditions in ESGRO complete plus medium (Millipore) supplemented with mouse leukemia inhibitory factor (ESGRO, Millipore). Cardiomyocyte induction for mouse ESCs was performed according to standard protocols as previously described with some modifications (Kattman et al., 2011). Briefly, cells were plated onto non-coated petri dishes in 7,500 cells/mL in SFD medium (IMDM:Ham's F12, 3:1; N2 supplement; B27 supplement; 0.05% BSA; Pen/Strep). Two days later (day 2), floating embryoid bodies (EBs) were collected, trypsinised, and plated onto petri dishes in 7,500 cells/mL in SFD medium supplemented with human recombinant VEGF (R&D, 5 ng/mL), Activin A (R&D, 5 ng/mL), and BMP4 (R&D, 0.25 ng/mL). At day 4, floating EBs were collected, trypsinised, and plated on 0.1% gelatin-coated 96 well dishes in 150,000 cells/well in StemPro34

medium supplemented with human recombinant VEGF (R&D, 5 ng/mL), bFGF (R&D, 10 ng /mL), and FGF10 (R&D, 5 ng/mL). Spontaneously beating cardiomyocytes were typically seen at day 8. Human ES cell line HUES7 was maintained on Matrigel-coated tissue culture dishes with mTeSR1 medium (STEMCELL Technologies,) according to the manufacturer's instructions. Cardiomyocyte induction from human ESCs or iPSCs was undertaken using a modified version of a previously reported protocol (Burridge et al., 2011). Briefly, cells were dissociated with Accutase (Invitrogen) and plated on Matrigel-coated 25cm^2 flasks at 2,000,000 cells/flask with mTesR1 medium containing 5 μM ROCK inhibitor Y-27632 (day -1). The following day (day 0), cells were dissociated with Accutase and suspended with RIP medium (RPMI contained with 4mg/mL polyvinyl alcohol, chemically defined lipid, Insulin-Transferrin-Selenium (ITS), 400 μM 1-Thioglycerol (MTG), 20 ng/mL BMP4, 6 ng/mL bFGF, 1 μM Y-27632 ROCK inhibitor) and plated into V-bottom 96-well dishes in 8,000 cell/well, then centrifuged at 950g for 5 min. Two days later (day 2), media were carefully aspirated and replaced with RD medium (RPMI with 20% FBS). Following two days (day 4), EBs were carefully transferred into U-bottom 96 well dishes with RI medium (RPMI containing chemically defined lipid, ITS, and MTG). Beating EBs were typically seen at day 9 to 10. Differentiated cardiomyocytes were assayed at differentiation day 10, because usually spontaneous beating areas of differentiated cardiomyocytes plateaued at day 10. If CPs were isolated via cell sorting, the cardiomyocytes were assayed on day 12, because spontaneous beating areas of differentiated cardiomyocytes plateaued at day 11 or 12 in many cases. This delay of differentiation might be caused by the stress on CPs associated with cell dissociation, FACS, and/or the re-plating procedure. Human ESCs were used under the license of the UK Steering Committee (reference number; SCSC13-25). Human iPSCs (iPS-HS1M) were originally established by D. Miller using human dermal fibroblasts (HDFs) from a healthy donor under informed consent (Health Research Authority approval 13/LO/0224), for a study to be described elsewhere (Miller et al., manuscript in preparation). HDFs were reprogrammed using the single polycistronic lentivector hSTEMCCA (a kind gift from T. McKay), with reprogrammed colonies isolated, clonally expanded, and one resulting iPS cell line iPS-HS1M characterised as described elsewhere (Miller et al., manuscript in preparation) (Somers et al., 2010).

Flow cytometry/FACS

Cells were treated with 3mM EDTA for 3 min at 37 °C to dissociate into single cells. Then they were washed once with FACS buffer (HBSS with 3% FBS and 0.03mM EDTA) and centrifuged for 3 min at 1,000 rpm. Cells were suspended with the primary antibody solution containing anti-human/mouse GFRA2 (R&D, 15 μg/mL), anti-mouse PDGFRA-PE (Abcam, 1:100) or anti-human PDGFRA-PE (Abcam, 1:10), anti-mouse KDR-Alexa647 (BioLegend, 1:100) or anti-human KDR-Alexa647 (BioLegend, 1:30), or anti-human KIT antibody (BD Bioscience, 1:10) for 45 min at room temperature. After washing with FACS buffer, cells were suspended with the secondary antibody solutions containing donkey anti-goat IgG Alexa 488 conjugated (Invitrogen, 1:500) and incubated at room temperature for 30 min. Dead cells were excluded by the gating of DAPI staining. Cells were sorted using FACS ARIA II or analysed by LSR Fortessa II or FACS Canto II (BD Biosciences) with FACSDiva 7.0 software. Sorted cells were plated on 0.1% gelatin-coated culture dishes with differentiation media based on StemPro34 (mouse ESCs), RPMI (human ESCs/iPSCs) with the growth factors shown above, or DMEM/10% FBS in the cases of mouse endothelial differentiation. For staining with anti-TNNT2 antibody (Hytest, 1:200), anti-mouse PECAM1 (BD Biosciences, 1:30), anti-human PECAM1 (Affymetrix, 1:30), anti-ACTA2 (Abcam, 1:100), cells were fixed and permeabilized by using DAKO Intra stain kit (DAKO) according to the manufacturer's instructions. For staining the mouse differentiating ES cells with anti-HCN4 (Millipore, 1:100), anti-TBX5 (SIGMA, 1:30), anti-ISL1 (DSHB 39.4D5, 1:100), anti-NKX2-5 (Santa Cruz, 1:50), firstly the cells were harvested by 3mM EDTA and treated with anti-GFRA2 antibody as described above. Then the cells were fixed and permeabilized by using DAKO Intra stain kit. The cells were incubated with those first antibodies at 4 °C overnight, then incubated with Alexa Fluor secondary antibodies. FACS Canto II was used for the analyses.

CRISPR/Cas9 mediated genome editing

The single guide RNA (sgRNA) sequences were designed with CRISPR Design Tool and sgRNA Designer as previously described (http://www.genome-engineering.org/crispr/) (Hsu et al., 2013), (http://www.broadinstitute.org/rnai/public/analysis-tools/sgrna-design) (Doench et al., 2014). To minimize off-target mutations, we selected the 20 bp genome target where at least 5 bases of the 5' end and 3 bases of the 3' end were distinct from any other sequences in the mouse genome based on BLAST searches (Fu et al., 2013). The synthesised oligonucleotides were cloned into the pX330 vector (Addgene) and were then co-transfected with pIRES-PURO (Clontech) into ESCs using FuGENE HD

(Roche) (Cong et al., 2013; Wang et al., 2013). Puromycin resistant clones were usually isolated 7 days after the initiation of puromycin selection and mutations in each clone were confirmed by Sanger sequencing of PCR products for the genomic region around the target sequences. Among the homozygously mutated clones, deletions/insertions of multiples of 3 bases were discarded because the possibility of in-frame translation of functional proteins could not be excluded. We confirmed the early termination of the translation into proteins by frame-shift mutations and selected at least 2 different clones for each gene. To exclude the possibility that any off-target effects might be responsible for the observed phenotypes, we designed two independent sgRNAs for each target gene. For direct genome editing in mouse embryos, we microinjected or electroporated sgRNAs and *Cas9* mRNAs into the fertilized eggs as previously described (Hashimoto and Takemoto, 2015; Wang et al., 2013). In brief, we harvested the fertilized eggs of F1 (C57BL/6 xCBA/ca) mice, and *in vitro* synthesized 100 ng/µL of sgRNA and 50 ng/µL of Cas9 mRNA were injected into one-cell stage embryos simultaneously (Wang et al., 2013). Alternatively, to introduce sgRNA and Cas9 mRNA, electroporation was performed as previously described (Hashimoto and Takemoto, 2015). One-cell stage F1 (C57BL/6 xC3H) embryos were placed in the 1 mm gap of the electrode (LF510-PT1-5, BEX) filled with 2.5µl of 1200 ng/µl *Cas9* mRNA and 200 ng/µl sgRNAs in Opti-MEM I medium (Gibco). The electric condition applied was two sets of three pulses (Voltage, 25 V; duration, 3 msec; interval, 50 msec; polarity change between the sets) generated by CUY EDIT II electroporator (BEX). Embryos were harvested at E8.5 and E17.5 after oviduct transfer of injected embryos. Yolk sac or tail tissues were subjected for the genome preparation to determine the genotype. PCR primers, sequencing primers and sgRNA design are listed in the table below. WT littermates determined by Sanger sequencing were used as controls in E8.5, and *mCherry* sgRNA plus *Cas9* mRNA transduced embryos were used as controls in E17.5.

Oligonucleotides for CRISPR/Cas9 mediated gene targeting

*Gfra1*_ES_1	F<br>R	CACCGCAGGCTGTCAGATATATTC<br>AAACGAATATATCTGACAGCCTGC
*Gfra2*_ES_1	F<br>R	CACCGCCTGTGACCTCCCGCCTCT<br>AAACAGAGGCGGGAGGTCACAGGC
Ret	F<br>R	CACCGCAGGTGTTCGATGCAGATG<br>AAACCATCTGCATCGAACACCTGC
GDNF	F<br>R	CACCGATGAAGTTATGGGATGTCG<br>AAACCGACATCCCATAACTTCATC
Neurturin	F<br>R	CACCGCGCTGGAAGGCAGCGGCCC<br>AAACGGGCCGCTGCCTTCCAGCGC
*Gfra2*_ES_2	F<br>R	CACCGCTCCAGGTACCGCACCCTT<br>AAACAAGGGTGCGGTACCTGGAGC
Cas9 mRNA synthesis	F<br>R	TTAATACGACTCACTATAGGGAGAATGGACTATAAGGACCACGAC<br>GCGAGCTCTAGGAATTCTTAC
Gfra1 sgRNA_1	F<br>R	TTAATACGACTCACTATAGGCAGGCTGTCAGATATATTC<br>AAAAGCACCGACTCGGTGCC
Gfra2 sgRNA_1	F<br>R	TTAATACGACTCACTATAGGCCTGTGACCTCCCGCCTCT<br>AAAAGCACCGACTCGGTGCC
Gfra1 sgRNA_2	F<br>R	TTAATACGACTCACTATAGTCAAGGCCTCCATAGCGCTG<br>AAAAGCACCGACTCGGTGCC
Gfra2 sgRNA_2	F<br>R	TTAATACGACTCACTATAGTCTTCATGCCCCGCTTGCAG<br>AAAAGCACCGACTCGGTGCC
Gfra2 sgRNA_3	F<br>R	TTAATACGACTCACTATAGGCCAGCCCTTCCTCTCCGCA<br>AAAAGCACCGACTCGGTGCC

PCR/Sequencing primers for genotyping

Gfra1<br>PCR_1	F<br>R	CTAACAGAACTAGGGCCGCT<br>GCCACCTACAGACAGCATTG
Gfra1<br>seq_1	F<br>R	TTCCGGATGGGCAGTGAAAC<br>ACCTCCCTCCAGGCACTTCC
Gfra2<br>PCR_1	F<br>R	AGTTTAGTCCTGGGTCTGCC<br>AACCGAGGATCCAGAGAAGC
Gfra2<br>seq_1	F<br>R	TGCCACTAGGCACACCTGCT<br>CCCCAGGGGTGAGAGGAAAC
Ret<br>PCR	F<br>R	CCCATGCTGCTTTGACCAG<br>GTTTACTTACTGGTGGGCGG
Ret<br>seq	F<br>R	CCTGCGCTGACTGGCACTCT<br>GGGCCTGGAATGGGTGTGCT
Gdnf<br>PCR	F<br>R	AAGTTGGAGGCTGGAGGAC<br>GGGTTTTAGCAAGGCACAGT
Gdnf<br>seq	F<br>R	TCTGAGCCTAACTTGCCTGG<br>GGGAGGGAACGGTTCTTACA
Neurturin<br>PCR	F<br>R	GGGTAAGATAGGAGGTGGCC<br>GTTCAGGAGGCAGGAGGATT
Neurturin<br>seq	F<br>R	CACTGTCCTCCCTCCATACC<br>CCCCAGGCACCTTTTCCA
Gfra2<br>PCR_2	F<br>R	AGGCCTACATTGGGGTCAGAGAC<br>TGGCCAGATCCATCCACCCC
Gfra2<br>seq_2	F<br>R	AGGCCTACATTGGGGTCAGAGAC<br>GGCCAGATCCATCCACCCC
Gfra1<br>PCR_2	F<br>R	CTGGAATCAGGGTACCCACTAGTCTGC<br>GACTGAGGTTGCCTCTGGCCTTC
Gfra1<br>seq_2	F<br>R	ATGTCGGCCGAGGTGAGT<br>CAGCGGCAGTTGTAGAGAGA
Gfra2<br>PCR_3	F<br>R	AGGTTGGGTCTACGTTGACCTTGGTC<br>AAGTTCTTCCTTCCTTCTGGATCCCTG
Gfra2<br>seq_3	F<br>R	TAGGCCTACATTGGGGTCAG<br>CCCAGATGGATGCTCCAAT

Western blot analysis

Cells were harvested with RIPA buffer with protease inhibitors then provided for sonication. After centrifugation at 14,000 rpm for 15 min, the supernatants were mixed with sample buffer (BioRad) with β-mercaptoethanol and boiled at 95 °C for 5 min. Protein concentrations were determined by the Lowry method and 5 μg of the samples were used for blotting. NuPAGE Bis-Tris 4-12% gradient gel (Thermo Scientific) and PVDF membrane (GE Healthcare) were used for electrophoresis and wet transfer. After blocking with 5% skimmed milk/TBS for 60 min, the primary antibodies, anti-GFRA1, anti-GFRA2, anti-RET, anti-GDNF, anti-Neurturin (all were purchased from Abcam), anti-phospho-FAK, anti-FAK, anti-phospho-ERK1/2, anti-ERK1/2 (Cell Signaling Technologies), anti-phospho-Fyn, anti-Fyn (Abcam), anti-TUBA1A (αTubulin; Abcam) were suspended with Can Get Signal solution 1 (TOYOBO), and membranes were incubated with antibodies at 4 °C overnight. Next day, membranes were incubated for 2 hours at room temperature in 5% skimmed milk containing horseradish peroxidase conjugated secondary antibodies (Thermo Scientific). Blots were visualized using Super Signal West Dura (Thermo Scientific) and scanned with an Alpha Imager HP Imaging System (Alpha Innotech) or ChemiDoc Touch (BioRad).

Culture of clonally FACS-isolated cells

Single cell sorting of human CPs was performed by FACS ARIA II according to manufacturer's instructions. Sorted cells were plated on mitomycin C treated mouse embryonic fibroblasts with 20% FBS/RPMI in 96-well plates. 5 μM of Y-27632 ROCK inhibitor was added overnight after sorting, then cells were cultured for a further 2 weeks with 20% FBS/RPMI for expansion and differentiation.

Reverse transcription and quantitative real-time PCR analysis

The total RNAs were extracted using an RNeasy mini kit (Qiagen) from each stage of differentiating ESCs or FACS-purified cells. 500 ng of total RNA for each specimen was used for reverse

transcription using SuperScript® II (Life Technologies) and oligo dT primers. Quantitative PCR was conducted in total volumes of 10 μL (0.25 μL of cDNA solutions) using a SYBR® Green PCR kit (Qiagen) and Rotor Gene 6000. PCR cycles were 95 °C for 10 s, 60 °C for 10 s, and 72 °C for 10 s according to the manufacturer's protocol. For conventional RT-PCR analyses, 50 ng of total RNAs were used for reverse transcription with High-Capacity cDNA reverse transcription kit (Thermo), and 0.5 μL of 20 μL cDNA solutions were provided for 35 to 45 cycles PCR with HotStar Taq (Qiagen). Sequences of the primers are provided in the Supplemental Table. Relative expression levels were calculated using the comparative CT method. *GAPDH* was used as a constitutively expressed internal control.

Mouse qPCR and RT-PCR primers

Gapdh	F<br>R	aactttggcattgtggaagg<br>acacattgggggtaggaaca
Gfra2	F<br>R	tttaacatgatcttggcaaacg<br>agcggagggtttcgtctaa
Tbx5	F<br>R	gagaatggtgtctctggccc<br>ttataggggtgctccgtgct
Nkx2-5	F<br>R	cggtgaccctgacccagccaa<br>gtagacctgcgcctgcgagaa
Mesp1	F<br>R	cagtccctcatctccgctct<br>gttgcattgtcccctccact
T	F<br>R	cagcccacctactggctcta<br>gagcctggggtgatggta
Myl2	F<br>R	ggacacatttgctgcccta<br>atcgtgaggaacacggtga
Gfra1	F<br>R	ttcccacacacgttttacca<br>gcccgatacattggatttca
Hcn4	F<br>R	ccatcaatggcatggtga<br>ccttgaagagggcgtagga
Isl1	F<br>R	ccctctcagtcccttgcat<br>gtcttctcgggctgtttgtg

Human qPCR and RT-PCR primers

GAPDH	F<br>R	agccacatcgctcagacac<br>gcccaatacgaccaaatcc
GFRA2	F<br>R	gcttggggaccagtgtcat<br>ctttggagttgttggccttc
TBX5	F<br>R	cgattcgaaacccgagag<br>gaaacactttgattccctcca
NKX2-5	F<br>R	gccttctatccacgtgccta<br>cctctgtcttctccagctcc
MYL2	F<br>R	gcaggcggagaggttttc<br>agttgccagtcacgtcagg
ISL1	F<br>R	aaggacaagaagcgaagcat<br>ttcctgtcatcccctggata
HCN4	F<br>R	ggtgtccatcaacaacatgg<br>gccttgaagagcgcgtag
TNNT2	F<br>R	gtcggcagctgctgttct<br>tcctctctccagtcctcctct
PECAM1	F<br>R	gcaacacagtccagatagtcgt<br>gacctcaaactgggcatcat
MYH11	F<br>R	aactcgtgtccaacctggaa<br>ttctcctcggctaacaactga

Transmission Electron Micrographs

Dissected 1 mm^3 tissue blocks from the hearts of E17.5 embryo were fixed in half Karnovsky fixative (1% of glutaraldehyde/1% of PFA/0.1 M of sodium phosphate, pH 7.3) for 2 h at 4 °C and post-fixed

in 2% of osmium tetroxide//0.1 M of sodium phosphate (pH 7.3) for 2h at 4°C. After dehydration with ethanol, the specimens were incubated in propylene oxide, and embedded Quetol 812 (Nisshin EM). Ultra-thin sections were stained with uranyl acetate and lead citrate, and observed with transmission electron microscope (Hitachi, H-7650) at 80 kV.

Statistical Analysis
Quantitative data are presented as means ± SEM (unless indicated otherwise) and were compared by using a Student's t test or one-way ANOVA. *P* values of <0.05 were considered as statistically significant.

SUPPLEMENTAL REFERENCES

Kurimoto, K., Yabuta, Y., Ohinata, Y., Ono, Y., Uno, K.D., Yamada, R.G., Ueda, H.R., and Saitou, M. (2006). An improved single-cell cDNA amplification method for efficient high-density oligonucleotide microarray analysis. Nucleic Acids Res *34*, e42.

Somers, A., Jean, J.C., Sommer, C.A., Omari, A., Ford, C.C., Mills, J.A., Ying, L., Sommer, A.G., Jean, J.M., Smith, B.W., *et al.* (2010). Generation of transgene-free lung disease-specific human induced pluripotent stem cells using a single excisable lentiviral stem cell cassette. Stem Cells *28*, 1728-1740.

Uehara, M., Yashiro, K., Takaoka, K., Yamamoto, M., and Hamada, H. (2009). Removal of maternal retinoic acid by embryonic CYP26 is required for correct Nodal expression during early embryonic patterning. Genes Dev *23*, 1689-1698.
