# Supplementary figures and images for: GFRA2 Identifies Cardiac Progenitors and Mediates Cardiomyocyte Differentiation in a RET-Independent Signaling Pathway
Source: Cell Rep. 2016 Jul 7;16(4):1026–38. doi: 10.1016/j.celrep.2016.06.050 (PMC4967477; doi:10.1016/j.celrep.2016.06.050)

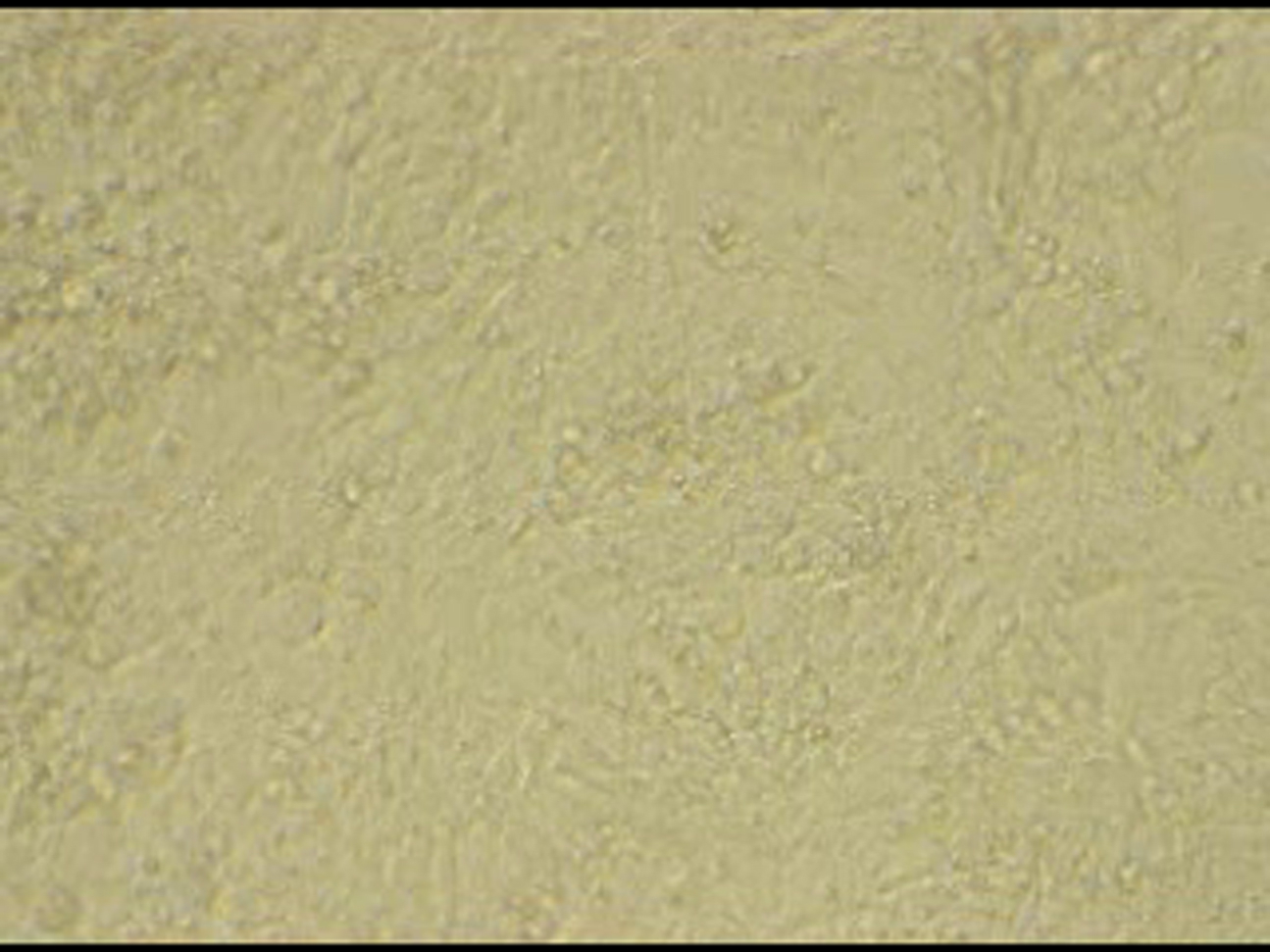

Supplement: Movie S1. Spontaneously Beating Cardiomyocytes Differentiated from GFRA2+/PDGFR-α+ Mouse Cardiac Progenitors after FACS Isolation, Related to Figure 2 — After an additional 5 days culture in differentiation media, GFRA2+/PDGFR-α+ cells showed synchronized spontaneous contraction, suggesting that they can effectively differentiate into functional cardiomyocytes. [file mmc2.jpg]

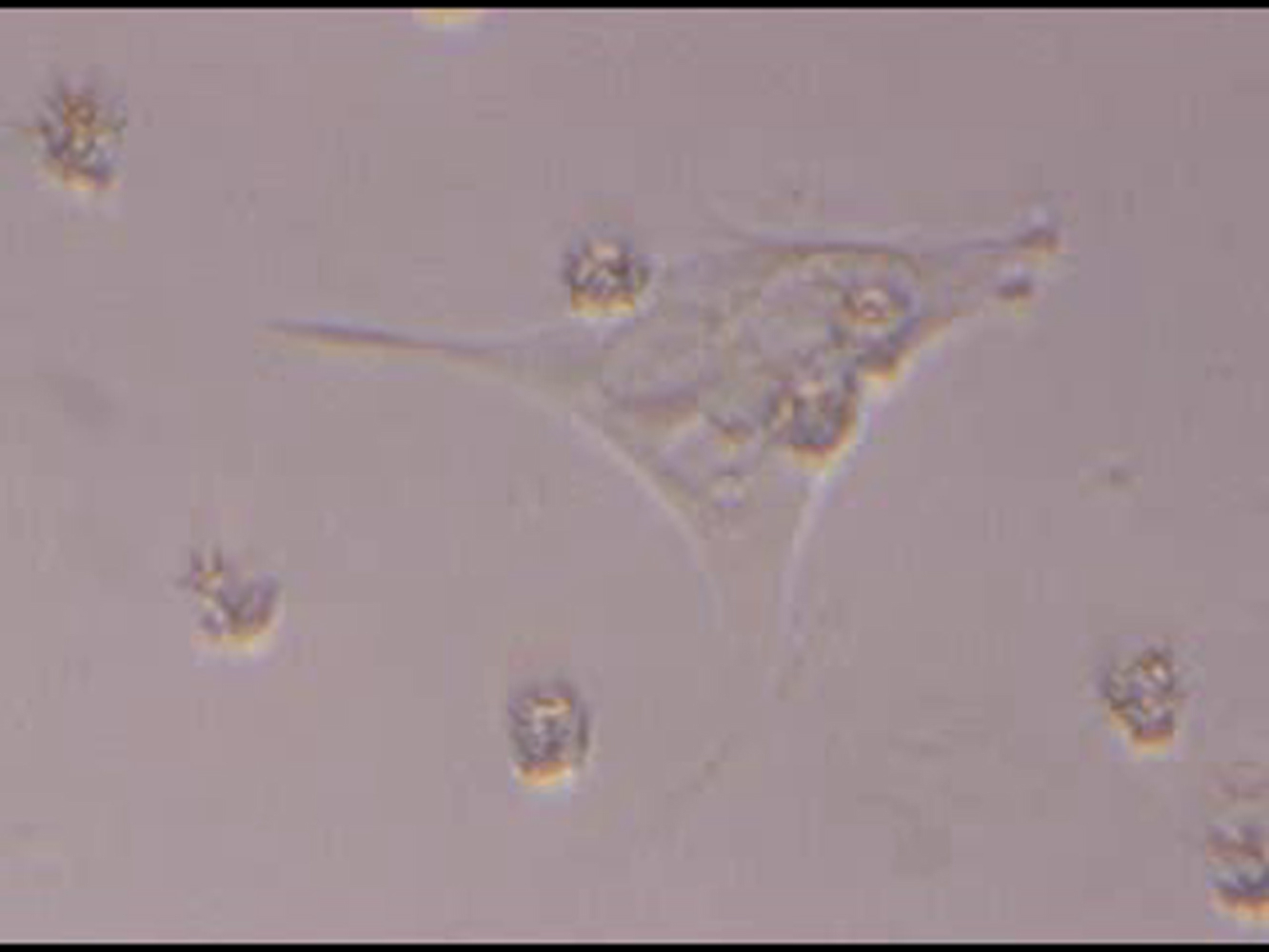

Supplement: Movie S2. Spontaneously Beating Cardiomyocytes Derived from FACS-Isolated hGFRA2+/hPDGFR-α+ Human Cardiac Progenitors, Related to Figure 4 — FACS-isolated hGFRA2+/hPDGFR-α+ cells from human ESCs differentiated into spontaneously beating cardiomyocytes. [file mmc3.jpg]
