# Supplementary material for: GFRA2 Identifies Cardiac Progenitors and Mediates Cardiomyocyte Differentiation in a RET-Independent Signaling Pathway
Source: Cell Rep. 2016 Jul 7;16(4):1026–38. doi: 10.1016/j.celrep.2016.06.050 (PMC4967477; doi:10.1016/j.celrep.2016.06.050)
Supplement: Document S2. Article plus Supplemental Information [file mmc4.pdf]

Article

Cell Reports

GFRA2 Identifies Cardiac Progenitors and Mediates Cardiomyocyte Differentiation in a RET-Independent Signaling Pathway

Graphical Abstract

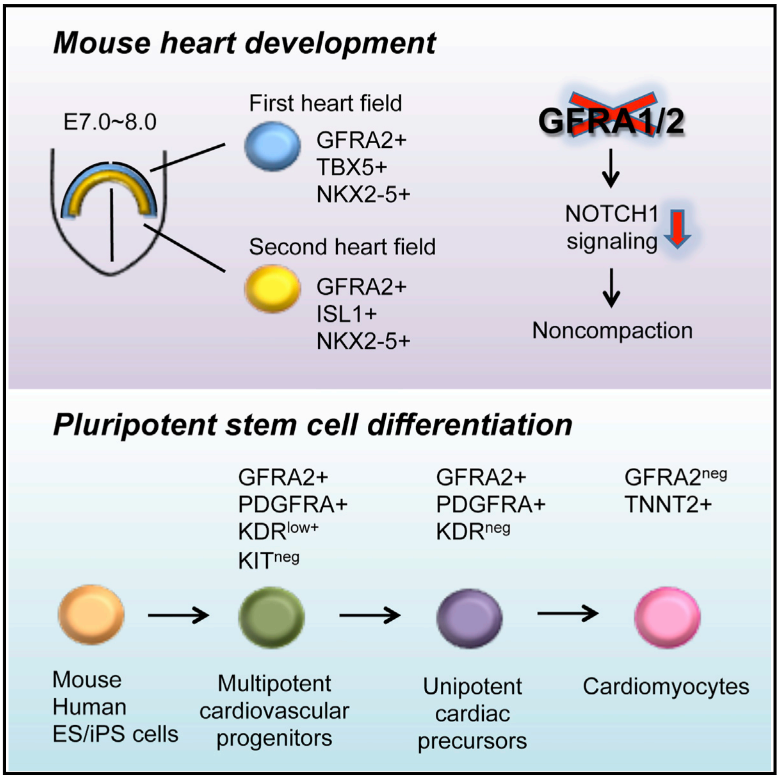

Authors

Hidekazu Ishida, Rie Saba, Ioannis Kokkinopoulos, ..., Keiichi Ozono, Ken Suzuki, Kenta Yashiro

Correspondence

drkyashiro@gmail.com

In Brief

Ishida et al. show that GPI-anchored neurotrophic factor receptor *Gfra2* specifically marks cardiac progenitor cells (CPs) in mouse and human, providing a method for isolating CPs. Unexpectedly, *Gfra2* plays a significant role in heart development via a non-canonical signaling pathway that is independent of known ligands and the co-receptor RET tyrosine kinase.

Highlights

- *Gfra2* is specific for cardiac progenitors (CPs) among mesodermal cells
- GFRA2 enables isolation of multipotent or unipotent CPs in mouse and human
- *Gfra2* and the related molecule *Gfra1* play a vital role in heart development
- The GFRA1/2 signal in heart development is independent of established ligands

Ishida et al., 2016, Cell Reports *16*, 1026–1038
July 26, 2016 © 2016 The Author(s).
http://dx.doi.org/10.1016/j.celrep.2016.06.050

OPEN ACCESS
CellPress

Cell Reports
Article

GFRA2 Identifies Cardiac Progenitors and Mediates Cardiomyocyte Differentiation in a RET-Independent Signaling Pathway

Hidekazu Ishida,[1,2,4] Rie Saba,[1,2] Ioannis Kokkinopoulos,[1,2] Masakazu Hashimoto,[7] Osamu Yamaguchi,[5] Sonja Nowotschin,[8] Manabu Shiraishi,[2] Prashant Ruchaya,[2,9] Duncan Miller,[3] Stephen Harmer,[3] Ariel Poliandri,[1] Shigetoyo Kogaki,[4] Yasushi Sakata,[5] Leo Dunkel,[1] Andrew Tinker,[3] Anna-Katerina Hadjantonakis,[8] Yoshiki Sawa,[6] Hiroshi Sasaki,[7] Keiichi Ozono,[4] Ken Suzuki,[2] and Kenta Yashiro[1,2,*]
[1]Centre for Endocrinology
[2]Translational Medicine and Therapeutics
[3]Cardiac Electrophysiology
William Harvey Research Institute, Barts and The London School of Medicine and Dentistry, Queen Mary University of London, London EC1M 6BQ, UK
[4]Department of Paediatrics
[5]Department of Cardiovascular Medicine
[6]Department of Cardiovascular Surgery
Osaka University Graduate School of Medicine, Osaka 565-0871, Japan
[7]Laboratory for Embryogenesis, Osaka University Graduate School of Frontier Biosciences, Osaka 565-0871, Japan
[8]Developmental Biology Program, Memorial Sloan Kettering Cancer Center, New York, NY 10065, USA
[9]Centre of Human and Aerospace Physiological Sciences, School of Biomedical Sciences, King's College, London, SE1 1UL, UK
*Correspondence: drkyashiro@gmail.com
http://dx.doi.org/10.1016/j.celrep.2016.06.050

SUMMARY

A surface marker that distinctly identifies cardiac progenitors (CPs) is essential for the robust isolation of these cells, circumventing the necessity of genetic modification. Here, we demonstrate that a Glycosylphosphatidylinositol-anchor containing neurotrophic factor receptor, Glial cell line-derived neurotrophic factor receptor alpha 2 (*Gfra2*), specifically marks CPs. GFRA2 expression facilitates the isolation of CPs by fluorescence activated cell sorting from differentiating mouse and human pluripotent stem cells. *Gfra2* mutants reveal an important role for GFRA2 in cardiomyocyte differentiation and development both in vitro and in vivo. Mechanistically, the cardiac GFRA2 signaling pathway is distinct from the canonical pathway dependent on the RET tyrosine kinase and its established ligands. Collectively, our findings establish a platform for investigating the biology of CPs as a foundation for future development of CP transplantation for treating heart failure.

INTRODUCTION

The heart is the first morphologically distinct developing organ in vertebrates. The primordial heart is derived from the anterior part of the lateral plate mesoderm as cardiac progenitors (CPs) being one of the earliest populations emerging from the primitive streak at gastrulation (Kinder et al., 1999; Rana et al., 2013). Lineage tracing experiments have led to the identification of CPs in the first (FHF) and second heart field (SHF) according to their anatomical origin and destiny (Rana et al., 2013). Recently, studies have delineated the complex molecular mechanisms underlying cardiomyocyte differentiation (Kathiriya et al., 2015; Paige et al., 2015); however, our knowledge of the precise spatiotemporal mechanisms that regulate the segregation, identity, and fate of CPs remains incomplete. A major hurdle is the paucity of reliable and specific markers to identify CPs, especially for the robust isolation of living CPs using cell sorting, circumventing the requirement of genetic modification for tagging CPs. Previous reports have demonstrated that Kinase insert domain receptor (KDR, also known as Flk-1), platelet-derived growth factor receptor alpha (PDGFRA), KIT, C-X-C chemokine receptor type 4 (CXCR4), and/or Prion protein (PrnP) can be used in defined combinations to identify and harvest CPs (Bondue et al., 2011; Hidaka et al., 2010; Kattman et al., 2006, 2011; Nelson et al., 2008; Yang et al., 2008). In vitro clonal-tracing studies have revealed that both KDR^{+}/PDGFRA^{+} and KDR^{low+}/KIT^{neg} cell populations contain highly enriched multipotent progenitors producing not only cardiomyocytes but also endothelial and smooth muscle cells in mouse and human, respectively (Kattman et al., 2006, 2011; Yang et al., 2008). Moreover, since the expression pattern of each of these factors in the embryo is dynamic and not specific for the cardiac lineage (Hidaka et al., 2010; Kataoka et al., 1997; McGrath et al., 1999; Yang et al., 2008), concerns have been raised about the purity of CPs harvested using these markers. More recently, a cell-surface protein, hyperpolarization-activated cyclic nucleotide-gated potassium channel 4 (HCN4), has been reported to be

 © 2016 The Author(s).
This is an open access article under the CC BY license (http://creativecommons.org/licenses/by/4.0/).
CrossMark

transiently specific for FHF CPs during the earliest phase of cardiomyogenesis (Später et al., 2013). However, because there is no commercially available antibody against the extracellular domain of this molecule, its use in cell-sorting experiments is limited. Thus, identification of a CP-specific surface antigen for which an antibody is readily available is essential for furthering our understanding of the critical early events in heart development.

In this study, we found that Glial cell line-derived neurotrophic factor receptor alpha 2 (*Gfra2*) specifically marks CPs of the FHF and SHF in mouse and human (Airaksinen and Saarma, 2002; Paratcha and Ledda, 2008). The specificity and expression pattern of *Gfra2* provides a reliable means to isolate stage-specific CPs with high purity. Strikingly, *Gfra2* is essential for heart development, whereas *Gfra1*, another member of the *Gfra* receptor family, is functionally redundant. Finally, we demonstrate that the pathway by which GFRA1/2 modulates heart development is independent of the classical *Gfra* receptor family signaling pathway via the RET proto-oncogene.

RESULTS

Gfra2 Specifically Marks Both FHF and SHF CPs

According to previous single-cell expression profiling of mouse embryonic CPs between days 7.5 and 8.0 post-conception (E7.5–E8.0), we observed that *Gfra2*, a specific receptor for a neurotrophic factor Neurturin (NRTN), was expressed in CPs but not in embryonic stem cells (ESCs) (42.31 ± 22.53 SEM of CPs versus 0.00 of ESCs in Reads Per Millions, respectively) (Brouilette et al., 2012; Kokkinopoulos et al., 2015). This was consistent with the data demonstrating that *Gfra2* was co-expressed within the cardiac mesoderm expressing *Mesp1* (Bondue et al., 2011). To confirm the expression pattern of *Gfra2* in mouse embryos, we conducted whole-mount in situ hybridization (WISH) analyses in serial stages of early mouse embryos and found that *Gfra2* was detected from the Early Allantoic Bud stage simultaneously with one of the earliest markers of CPs, *Isl1*, and was clearly expressed in the cardiac crescent from E7.5 to E8.5 (Figures 1A and S1A) (Downs and Davies, 1993; Kokkinopoulos et al., 2015). Single-cell expression profiling suggests that *Isl1* precedes *Gfra2*, because *Isl1*-expressing CPs had a higher incidence of expression of *Gfra2* after they had started to express a common marker for the FHF and SHF, *Nkx2-5*, as they further differentiated (Figure S1B) (Kokkinopoulos et al., 2015). Thereafter, *Gfra2* was downregulated in the heart field by the ten-somite stage (E8.75) upon formation of the heart tube (Figure 1A). Immunofluorescence micrographs indicated that GFRA2 protein was prominently detected in the early headfold (EHF) stage CPs (Figure 1B) (Downs and Davies, 1993). Serial sections of the three-somite stage embryos revealed that GFRA2 co-localized with NKX2-5, TBX5, HCN4 (the FHF), and ISL1 (the SHF) (Figures 1C and S1C) (Cai et al., 2003; Devine et al., 2014; Kokkinopoulos et al., 2015; Liang et al., 2013; Später et al., 2013; Stanley et al., 2002). Therefore, GFRA2 can be considered a marker of CPs within both the FHF and SHF in mouse embryos.

GFRA2 Predominantly Identifies CPs Derived from Pluripotent Stem Cells

Next, to clarify whether GFRA2 marks CPs derived from pluripotent stem cells, we investigated the expression of *Gfra2* during cardiac differentiation of mouse ESCs. Quantitative real-time reverse transcription PCR (qPCR) demonstrated that *Gfra2* was transiently expressed with its peak at differentiation day 7, before the initiation of spontaneous beating of differentiated cardiomyocytes (Figure 2A). Flow cytometry using an antibody raised against the extracellular domain of GFRA2 revealed that GFRA2 could be identified between differentiation days 4–9, among the PDGFRA positive mesodermal cells (Figures 2B and S2A). Upon culturing the separately isolated cell populations of $GFRA2^{+}/PDGFRA^{+}$, $GFRA2^{neg}/PDGFRA^{+}$, and $GFRA2^{neg}/PDGFRA^{neg}$ at differentiation day 7 by fluorescent activated cell sorting (FACS), the majority of $GFRA2^{+}/PDGFRA^{+}$ cells differentiated into $TNNT2^{+}$ and ACTN1 (α-ACTININ)$^{+}$ cardiomyocytes, without a propensity for differentiation to endothelial cells and smooth muscle cells (Figures 2C–2F, S2B, and S2C; Movie S1). By contrast, the other cell populations rarely contained such cardiac cells. These results suggest that $GFRA2^{+}/PDGFRA^{+}$ cells at day 7 are already committed to a cardiomyocyte fate but remain as precursor cells without terminal differentiation. The expression of NKX2-5 (common), TBX5 (FHF), HCN4 (FHF), and ISL1 (SHF) in $GFRA2^{+}/PDGFRA^{+}$ cells demonstrated that $GFRA2^{+}/PDGFRA^{+}$ CPs reside in both the FHF and SHF (Figures 2G and S2D) (Cai et al., 2003; Devine et al., 2014; Kokkinopoulos et al., 2015; Liang et al., 2013; Später et al., 2013; Stanley et al., 2002). These data were consistent with our histological results in mouse embryos (Figures 1C and S1C).

To further characterize $GFRA2^{+}$ cells during cardiac differentiation, we investigated the relationship between GFRA2-expressing CPs and the well-validated earliest CPs of the $KDR^{+}/PDGFRA^{+}$ population in differentiating mouse ESCs (Kattman et al., 2006, 2011). KDR and PDGFRA were already expressed by day 3 of differentiation as previously described (Kattman et al., 2011), and KDR expression was downregulated in the $PDGFRA^{+}$ population at day 6 (Figure 3A). Of note, from day 4 to 5, almost the entire $KDR^{+}/PDGFRA^{+}$ population expressed GFRA2. Thus, GFRA2 could also mark the earliest mouse CPs in cardiac differentiating ESCs. When we isolated a $GFRA2^{+}/KDR^{+}/PDGFRA^{+}$ triple-positive population on day 4 and cultured the cells for a further 7 days, they gave rise not only to cardiomyocytes, but also to endothelial cells (Figure 3B). Given that GFRA2 marked $KDR^{+}/PDGFRA^{+}$ CPs on differentiation day 4, the earliest $GFRA2^{+}$ CPs would be expected to be multipotent based on previous reports (Bondue et al., 2011; Kattman et al., 2011). Thereafter, KDR expression would be limited to the endothelial lineage, with cardiomyogenic cells having lost KDR expression after day 6 of differentiation (Figures 3A, S2E, and S2F). Taken together, our findings clearly demonstrate that GFRA2 facilitates the robust isolation of CPs from differentiating mouse ESCs.

GFRA2 Marks Human CPs from Differentiating Human Pluripotent Stem Cells

To challenge whether human GFRA2 can be used for CP isolation from human pluripotent stem cells, we investigated the expression of human *GFRA2* during the cardiac differentiation of human

OPEN
ACCESS
CellPress

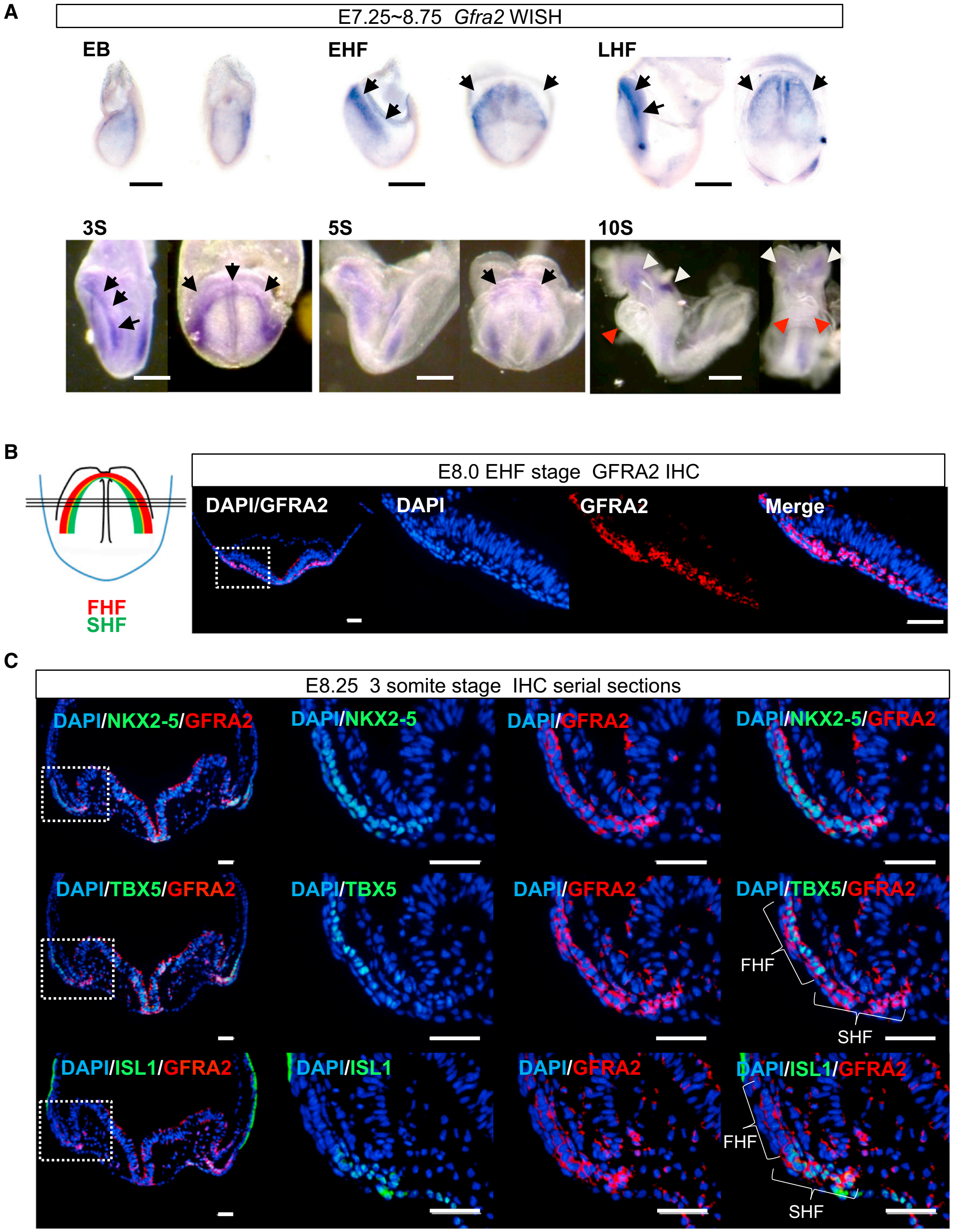

Figure 1. GFRA2 Is Expressed in the First and Second Heart Field Cardiac Progenitor Cells

(A) Whole-mount in situ hybridization (WISH) of *Gfra2* in E7.25–8.75 early allantoic bud (EB) stage to the ten-somite (S) stage embryos. *Gfra2* was expressed in the cardiac crescent (arrows). Once the heart tube is formed and the looping initiated, *Gfra2* is downregulated (red arrowheads). *Gfra2* was also expressed in migrating neural crest cells and the rhombomere 4 (white arrowheads). n = 3. Scale bar, 250 μm. EHF, early headfold; LHF, late headfold.

(B and C) Immunohistochemical (IHC) images of GFRA2 in an EHF and three-somite stage embryo. GFRA2 was expressed in the mesodermal regions corresponding to the heart field. n = 4. Scale bar, 50 μm. FHF, first heart field; SHF, second heart field.

See also Figure S1.

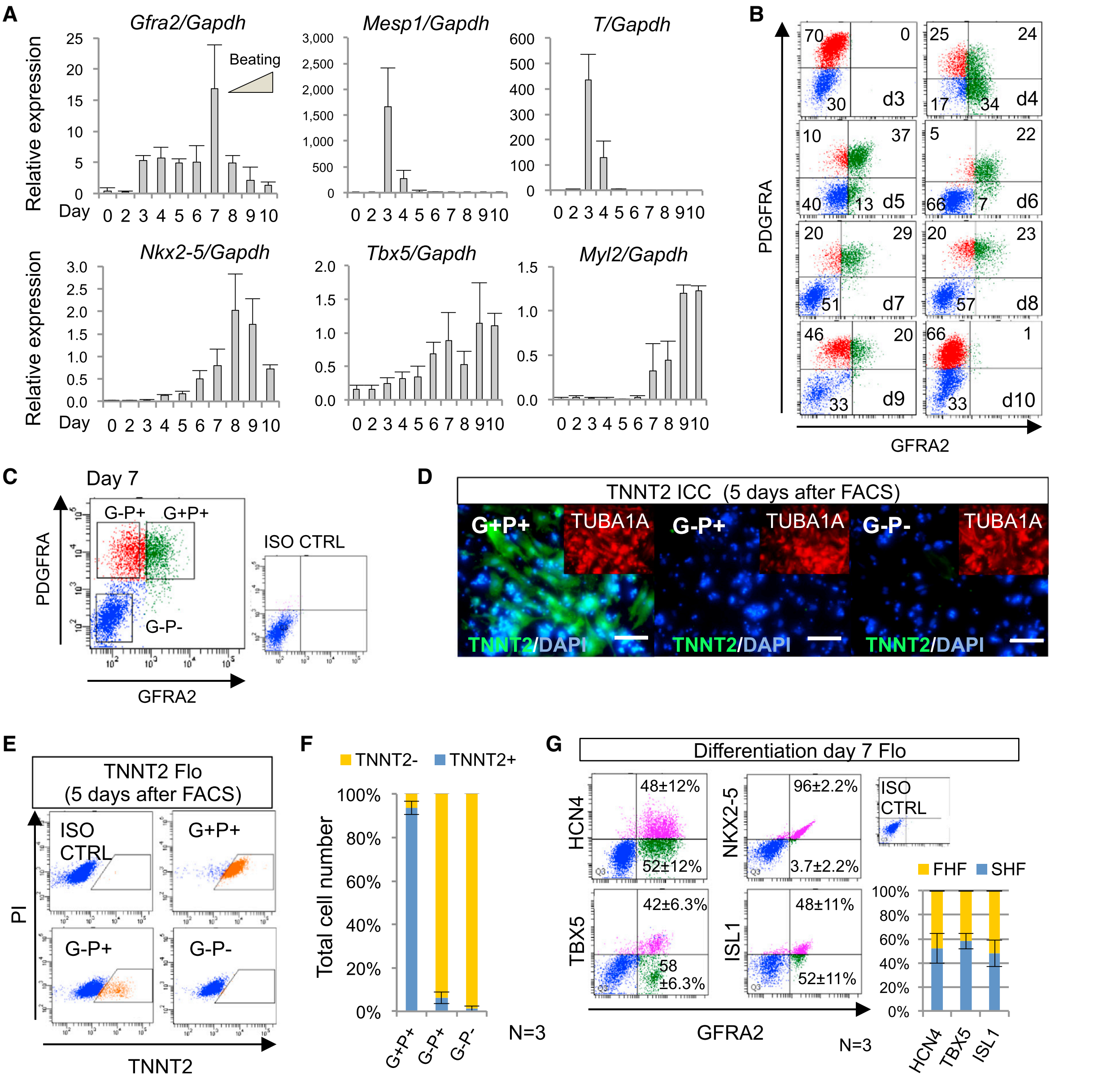

Figure 2. GFRA2⁺/PDGFRA⁺ Cells Derived from Mouse ESCs Are Unipotent Cardiac Precursors

(A) qPCR analyses of *Gfra2*, *Mesp1*, *T* (*Brachyury*), *Nkx2-5*, *Tbx5*, and *Myl2*. The peak of *Gfra2* expression was observed just before the initiation of spontaneous beating of cardiomyocytes. Note that mesodermal induction represented by *Mesp1* and *T* peaked at day 3 and sarcomeric protein synthesis of cardiomyocyte was apparent from day 8. Data are representative of biological triplicates with technical duplicates as mean ± SEM.

(B) Flow cytometrical (Flo) analyses show transient expression of GFRA2 during cardiomyocyte differentiation. GFRA2 was detected in the PDGFRA$^+$ mesodermal population from day 4 to day 9.

(C) FACS isolation at day 7 of differentiation. GFRA2$^+$/PDGFRA$^+$ (G$^+$P$^+$), GFRA2$^-$/PDGFRA$^+$ (G$^-$P$^+$), GFRA2$^-$/PDGFRA$^-$ (G$^-$P$^-$) populations were separately isolated.

(D) Immunocytochemistry (ICC) of TNNT2 in FACS-isolated cells after an additional 5 days culture in differentiation media. The condensed high DAPI signals represent dead cells. We counterstained with TUBA1A (α-tubulin) to delineate the live cells and found that most of G$^+$P$^+$ cells differentiated into TNNT2$^+$ cardiomyocytes. Scale bar, 100 μm.

(E and F) Flow cytometrical (Flo) analyses for TNNT2 revealed that most of the G$^+$P$^+$ cells (93.6% ± 3.1%) differentiated into cardiomyocytes. n = 3.

(G) Quantitative analyses by Flo for HCN4, NKX2-5, TBX5, and ISL1 in GFRA2$^+$ CPs at day 7. Bar graph represents of the proportion of FHF and SHF as mean ± SEM. n = 3.

See also Figure S2 and Movie S1.

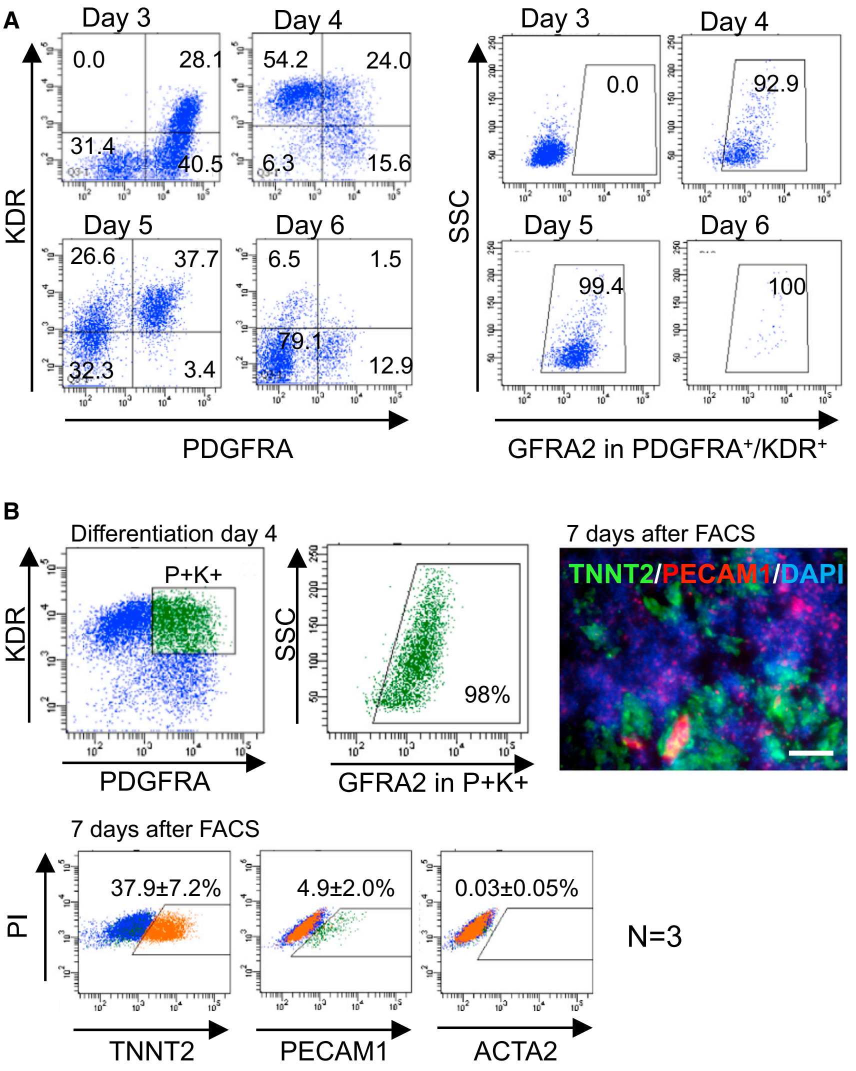

Figure 3. GFRA2$^+$/KDR$^+$/PDGFRA$^+$ Represents Multipotent CPs

(A) Flo analyses for KDR, PDGFRA, and GFRA2. KDR$^+$/PDGFRA$^+$ cells were observed at day 3, but these were GFRA2 negative. After day 4, most of KDR$^+$/PDGFRA$^+$ CPs were GFRA2 positive (Kattman et al., 2011). KDR expression was significantly downregulated at day 6.

(B) FACS isolation of GFRA2$^+$/KDR$^+$/PDGFRA$^+$ population at differentiation day 4. Immunocytochemical and flow cytometrical analyses for TNNT2, PECAM1, and ACTA2 (α- smooth muscle cell actin) reveal that this population differentiated into both cardiomyocytes and endothelial cells. This is consistent with the previous study (Kattman et al., 2011), which shows this population is multipotent cardiovascular progenitors. Scale bar, 100 μm.

See also Figure S2.

ESCs or induced pluripotent stem cells (iPSCs) (Burridge et al., 2011). Consistent with our results for the mouse, qPCR analyses showed human *GFRA2* was induced with a peak just before the appearance of spontaneously beating cardiomyocytes at day 8 of differentiation, both in ESCs and iPSCs (Figures 4A and S3A). We also identified the hGFRA2$^+$/hPDGFRA$^+$ cell population at day 8 by flow cytometry, with FACS-isolated hGFRA2$^+$/hPDGFRA$^+$ cells efficiently differentiating into TNNT2$^+$ cardiomyocytes when cultured for an additional 5 days (Figures 4B–4D and S3B–S3D). These TNNT2$^+$ cardiomyocytes demonstrated spontaneous beating (Movie S2). The expression profiles of FACS-purified hGFRA2$^+$/hPDGFRA$^+$ cells suggest that this population contains both the FHF and SHF CPs, similarly as in the case of mouse ESCs (Figures 2G, 4E, and S2D). Thus, labeling with antibodies raised to human GFRA2 also enables the isolation of a CP population from human pluripotent stem cells, without the need of lineage tagging by genetic modification.

To elucidate additional details concerning the earliest phase of hGFRA2$^+$ CPs in human cardiac differentiation, we performed flow cytometry using hGFRA2, hPDGFRA, hKDR, and hKIT antibodies at day 4 of differentiation. We found that the proportion of hGFRA2-expressing cells among the multipotent CP-enriched population of hKDR^{low+}/hPDGFRA$^+$ cells were less than in the case of mouse (Figures 3A and 4F) (Kattman et al., 2011). As expected, the hKDR^{low+}/hPDGFRA$^+$ population was hKIT negative, whereas hKDR$^+$/hPDGFRA^{neg} population was hKIT positive (Figure 4F) (Yang et al., 2008). By separating isolated hGFRA2$^+$/hKDR^{low+}/hPDGFRA$^+$/hKIT^{neg} and hGFRA2^{neg}/hKDR^{low+}/hPDGFRA$^+$/hKIT^{neg} populations, we found that the GFRA2 negative population almost lacked cardiomyogenic ability (Figure S3E). To confirm multipotency of human GFRA2 positive cells, we performed clonal lineage-tracing experiments. A single hGFRA2$^+$/hKDR^{low+}/hPDGFRA$^+$/hKIT^{neg} cell at day 4 was cloned by FACS and cultured for 2 weeks. RT-PCR using cardiomyocyte (*hTNNT2*), endothelial cell (*hPECAM*), and smooth muscle cell (*hMYH11*) markers clearly indicated the existence of the multiple cell lineages derived from a single cell, which strongly supports the multipotency of hGFRA2$^+$/hKDR^{low+}/hPDGFRA$^+$/hKIT^{neg} cells (Figure 4G). This is consistent with the data from mouse ESCs and previous work (Kattman et al., 2006, 2011; Yang et al., 2008). After differentiation day 4, as cardiomyocyte differentiation progressed, GFRA2$^+$/PDGFRA$^+$ CPs lost KDR expression by day 8 as observed in mouse ESCs differentiation (Figure S3F). Taken together, as in the mouse, hGFRA2$^+$/hKDR^{low+}/hPDGFRA$^+$/hKIT^{neg} CPs at day 4 are multipotent CPs, and hGFRA2$^+$/hKDR^{neg}/hPDGFRA$^+$/hKIT^{neg} at day 8 are unipotent cardiac precursors.

A Non-canonical Signaling Cascade via GFRA1/2 Is Indispensable for Cardiomyocyte Differentiation of Pluripotent Stem Cells

To elucidate a physiological function of GFRA2 in cardiac differentiation, we generated *Gfra2* knockout (KO) mouse ESC lines

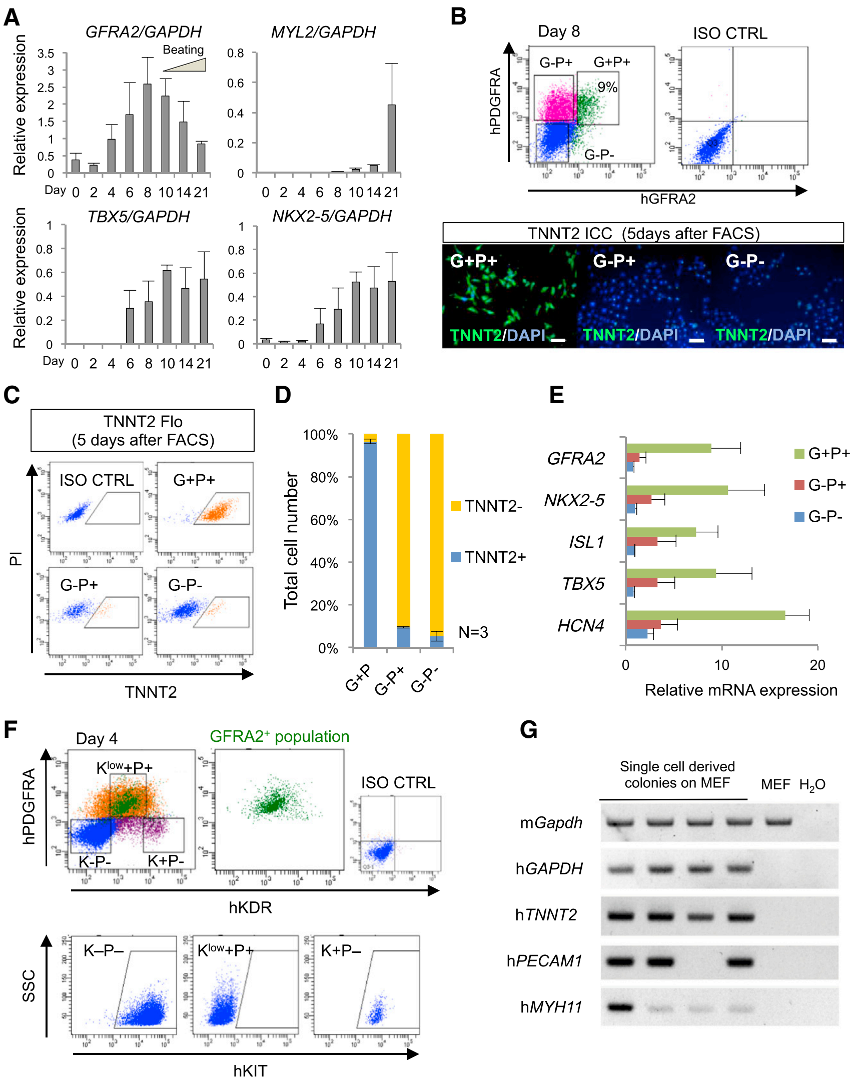

Figure 4. Human GFRA2 Marks CPs from Human ESC Cultures

(A) qPCR analyses for human *GFRA2*, *NKX2-5*, *TBX5*, and *MYL2*. Note the peak of human *GFRA2* expression is just before human embryoid bodies start to beat. Bar graph represents biological triplicates with technical duplicates as mean ± SEM.

(B) FACS isolation of human hGFRA2$^+$/hPDGFRA$^+$ (G^+P^+) cells. ICC analyses for TNNT2 demonstrate that isolated G^+P^+ population is highly cardiomyogenic. Scale bar, 100 μm.

(C and D) Quantitative analyses by Flo for TNNT2 show most of G^+P^+ cells (96.5% ± 1.1%) differentiated into cardiomyocytes. Bar graph represents mean ± SEM. n = 3.

(E) qPCR analyses for *GFRA2*, *NKX2-5*, *ISL1*, *TBX5*, and *HCN4* in FACS-isolated populations. All cardiac marker gene expressions were significantly higher in the G^+P^+ population when compared to the G^-P^- population (*p < 0.05, Student's t test). Data are representative of biological triplicates with technical duplicates as mean ± SEM.

(F) Flow cytometry of human ESCs at differentiation day 4. hGFRA2$^+$ cells (shown as green dots) were mostly included by hPDGFRA$^+$/hKDR^{low+}/hKIT^{neg} population, which is reported as multipotent CPs (Yang et al., 2008). 15%–20% of hPDGFRA$^+$/hKDR^{low+}/hKIT^{neg} cells were hGFRA2$^+$.

(G) RT-PCR analyses of single-cell-derived colonies for each lineage marker gene. A single hGFRA2$^+$/hKDR^{low+}/hPDGFRA$^+$ cell at day 4 was clonally sorted and cultured on mouse embryonic fibroblasts (MEFs) for 2 weeks. RT-PCR of *hTNNT2*, *hPECAM1*, and *hMYH11* represent the existence of cardiomyocytes, endothelial cells, and smooth muscle cells among the cells derived from a single cell, respectively. Note the existence of multiple lineages, which indicates the multipotency.

See also Figure S3 and Movie S2.

using the CRISPR/Cas9 genome editing system (Figures 5 and S4) (Cong et al., 2013; Wang et al., 2013). After 10 days of cardiac differentiation, two independent lines of *Gfra2*-KO ESCs did not show significant defects in cardiomyocyte differentiation, although a minor decrease in the number of differentiated cardiomyocytes was observed without statistical significance (Figures 5A–5C). This is consistent with the phenotype of KO mice that showed no cardiac defects (Airaksinen and Saarma, 2002; Hiltunen et al., 2000; Paratcha and Ledda, 2008; Rossi et al., 1999, 2003). Since GFRA1, another member of GFRA-family receptor whose specific ligand is glial cell line-derived neurotrophic factor (GDNF), might be functionally redundant, we generated compound mutant of *Gfra1/2* double-KO (DKO) ESCs (Airaksinen and Saarma, 2002; Baloh et al., 2000). Whereas *Gfra1* KO lines exhibited no significant defect in cardiomyocyte differentiation (Enomoto et al., 1998), the simultaneous ablation of *Gfra1* in addition to *Gfra2* significantly suppressed cardiomyocyte differentiation (Figures 5A–5C and S4A–S4E). It is unlikely that an off-target mutation is responsible for this phenotype, because two independent single guidance (sg) RNA targeting different portions of the gene resulted in an indistinguishable phenotype (Figures S4C and S4D) (Enomoto et al., 1998; Fu et al., 2013; Hiltunen et al., 2000; Rossi et al., 1999, 2003). Thus, *Gfra1/2* are required for cardiomyocyte differentiation in vitro, and *Gfra1* is functionally redundant for *Gfra2*. Interestingly, we found that *Gfra1* expression was significantly increased in *Gfra2* KO ESCs (Figure 5D). By contrast, *Gfra2* expression was unchanged in *Gfra1* KO ESCs. This result suggests that the loss of *Gfra2* can be compensated for by upregulated *Gfra1*, whereas *Gfra1* appears to be dispensable for cardiac differentiation (Baloh et al., 2000; Paratcha and Ledda, 2008; Scott and Ibanez, 2001). This suggestion is supported by the fact that *Gfra1* is not expressed in the heart field in vivo (Figure S5A).

The canonical signaling cascade acting via GFRA2 depends on a single-pass transmembrane protein, RET tyrosine kinase (Airaksinen and Saarma, 2002). When the specific ligand, neurturin (NRTN) binds GFRA2, the RET tyrosine kinase is activated by GFRA2/NRTN, to elicit a biological response. To confirm

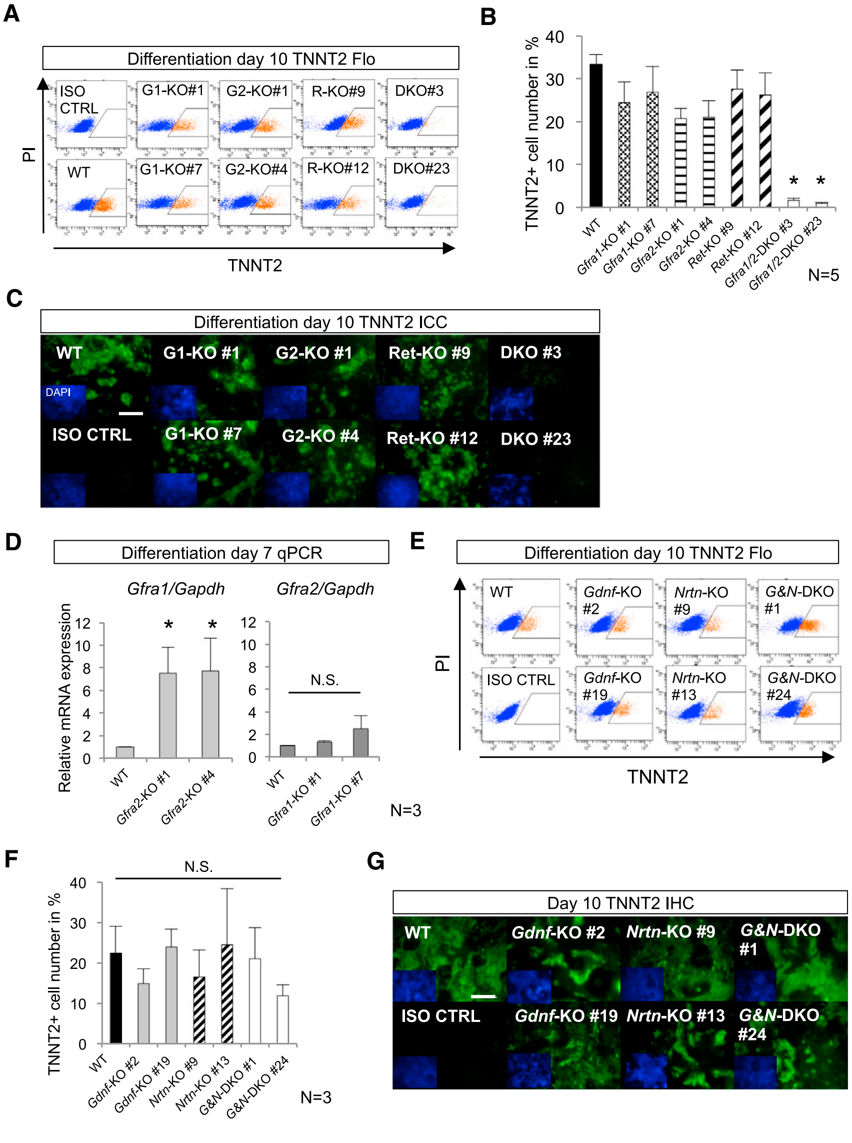

Figure 5. *Gfra1* and *Gfra2* Are Essential for In Vitro Cardiomyocyte Differentiation from Mouse ESCs

(A) Flo analyses of TNNT2 at day 10 of differentiation. WT, wild-type; G1-KO, *Gfra1*-KO; G2-KO, *Gfra2*-KO; DKO, *Gfra1/2*-DKO; R-KO, *Ret*-KO; ISO CTRL, isotype control.

(B) Quantitative analyses of flow cytometry show the severe impairment of cardiomyocyte differentiation in *Gfra1/2* DKO ESC lines. $^*p < 0.05$ versus WT in Student's t test. n = 5.

(C) Immunocytochemical analyses of each KO ESC line 10 days after induction of cardiomyocyte differentiation. *Gfra1/2* double-KO (DKO) ESCs exhibited severe defects in $TNNT2^+$ cardiomyocyte differentiation. Scale bar, 100 μm.

(D) qPCR analyses for *Gfra1* and *Gfra2* in *Gfra2* KO ESC lines and in *Gfra1* KO ESC line at day 7, respectively. Note the elevated expression of *Gfra1* in *Gfra2* KO ESCs. $^*p < 0.05$ versus WT in Student's t test. N.S., not significant. Bar graph represents mean ± SEM. n = 3.

(E and F) Flow cytometrical analyses of TNNT2 in differentiation day 10 ESC lines. *G&N*, *Gdnf*, and *Nrtn*. *Gdnf*- and *Nrtn*-null ESC lines did not show any statistically significant difference in cardiomyocyte differentiation efficiency compared to WT. N.S., not significant versus WT. Bar graph represents mean ± SEM. n = 3. (F) Immunofluorescent images of TNNT2 in ESC lines at differentiation day 10. Scale bar, 100 μm.

(G) See also Figures S4–S6.

whether the GFRA2 signaling pathway affecting cardiomyocyte differentiation depends on RET, we generated *Ret* KO ESCs lines. As expected from the phenotypes of KO mice, targeting of *Ret* did not resulted in significantly impaired cardiomyocyte differentiation (Figures 5A–5C, S4A, and S4B) (Airaksinen and Saarma, 2002; Baloh et al., 2000; Paratcha and Ledda, 2008; Schuchardt et al., 1994). This observation is also supported by the fact that RET was not expressed in the heart fields (Figure S5A). In addition, the KO lines of *Nrtn*, *Gdnf*, and *Nrtn/Gdnf* also did not show a significant defect (Figures 5E–5G, S5B, and S5C) (Golden et al., 1999; Heuckeroth et al., 1999; Sánchez et al., 1996). Collectively, these results indicate that cardiac differentiation signaling via GFRA2 is independent of the co-receptor RET tyrosine kinase.

Previous studies have reported that the direct interaction between Neural Cell Adhesion Molecule (NCAM1) and GFRA1 mediates an alternative GFRA1 signaling pathway via FAK/FYN operating in the absence of any secreted ligands and RET (Paratcha and Ledda, 2008; Paratcha et al., 2003; Sjöstrand et al., 2007). Although NCAM1 was not expressed in the heart field (Figure S5A), we cannot exclude the possibility that another cell adhesion molecule mediates a similar signal pathway. To confirm whether a similar pathway is responsible for the cardiac signaling of GFRA2, we investigated the phosphorylation of FAK, FYN, and its downstream ERK1/2 in *Gfra1/2* DKO ESCs during cardiomyocyte differentiation (Figure S6). Western blot analyses demonstrated that FAK phosphorylation was slightly but significantly elevated in *Gfra1/2* DKO ESCs, whereas FYN and ERK1/2 phosphorylation were unaffected (Figure S6A). Since the NCAM1/GFRA1 signal pathway first activates FYN and phosphorylated-FYN activates FAK (Paratcha et al., 2003), it is unlikely that a signal similar to the NCAM1/GFRA1 signal mediated by a cell adhesion molecule is operating. Thus, it suggests that FAK phosphorylation in *Gfra1/2* DKO ESCs becomes elevated by an unknown mechanism. We further tested whether the attenuation of elevated FAK could rescue the phenotype of *Gfra1/2* DKO ESCs, since it has been previously reported that activated FAK signaling impaired cardiomyocyte differentiation (Hakuno et al., 2005). We administered the FAK inhibitor PF-573228 to *Gfra1/2* DKO ESCs during their differentiation. However, the

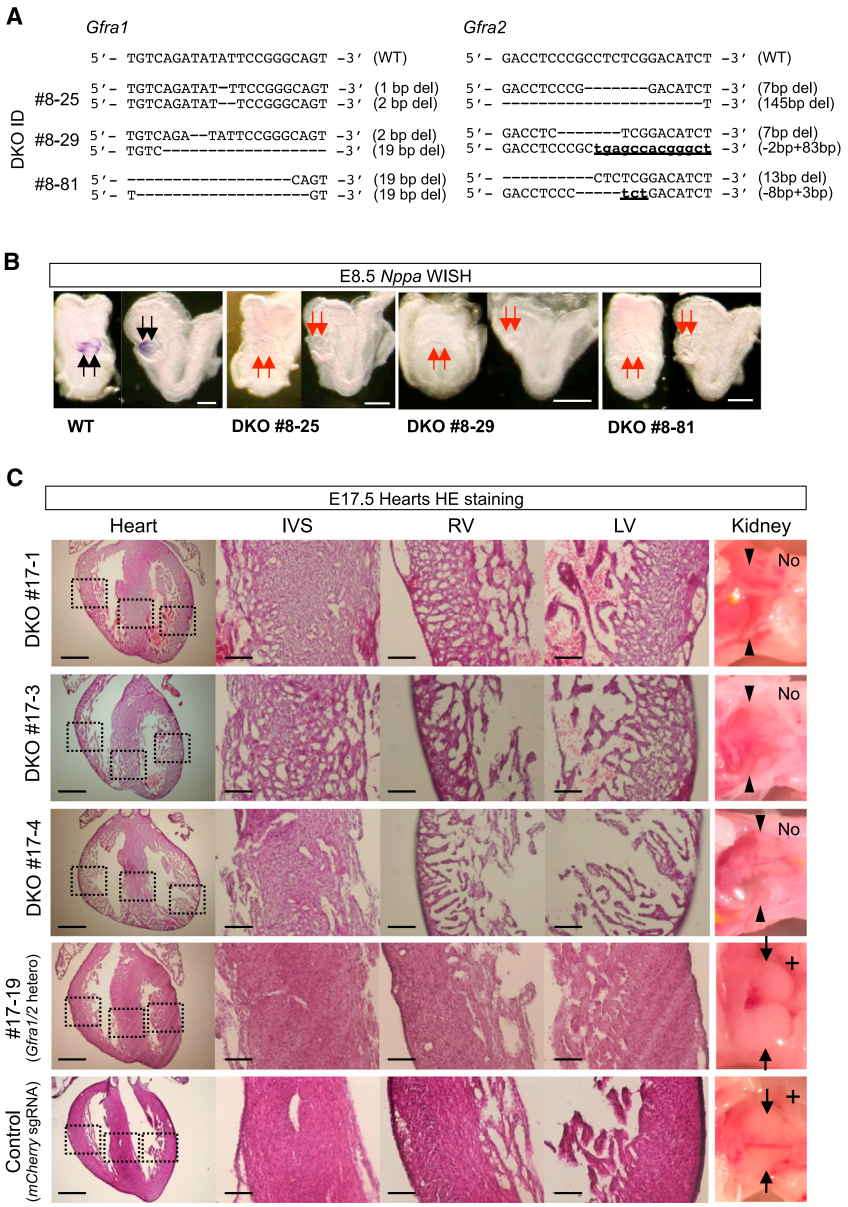

Figure 6. *Gfra1/2* Play Important Roles for the Heart Development In Vivo

(A) Genotype of the *Gfra1/2* DKO mouse embryos generated by the direct injection of *Gfra1*-targeted sgRNA, *Gfra2*-targeted sgRNA, and *Cas9* mRNA into zygotes.

(B) WISH analyses of differentiated cardiomyocyte marker *Nppa* in *Gfra1/2* DKO and WT littermate embryos at E8.5. *Nppa* expressions disappeared in DKO embryos (red arrows). Scale bar, 250 μm.

(C) H&E staining for the hearts of *Gfra1/2* DKO E17.5 embryos. The compaction layers of myocytes were thin, and the alignments of cardiomyocytes were impaired in *Gfra1/2* DKO embryos as compared to the control hearts (*mCherry* sgRNA and Cas9 mRNA transduced embryos) and *Gfra1/2* compound heterozygote mutant. *Gfra1* null resulted in kidney agenesis as previously described (black arrowheads, No), whereas the well-developed kidney was observed in the heterozygotes and WT (black arrows, +) (Enomoto et al., 1998). The mutation of each embryo induced by CRISPR/Cas9 is shown in Figure S7B.

IVS, intraventricular septum; RV, right ventricle; LV, left ventricle. Scale bar, 500 μm in whole-heart images and 100 μm in higher magnification. See also Figure S7.

efficiency of cardiomyocyte differentiation showed no improvement even though FAK phosphorylation was kept within physiological levels (Figures S6B and S6C). Therefore, the upregulation of FAK signaling was not primarily responsible for the defect of *Gfra1/2* DKO ESCs. This signaling pathway operating via GFRA2 during cardiomyogenesis must activate effectors of an alternative and critical circuit for cardiomyocyte differentiation.

Gfra1/2 Are Required for Ventricular Compaction In Vivo

To exclude the possibility that the phenotype observed in *Gfra1/2* DKO ESCs is an in vitro phenomenon, we generated *Gfra1/2* DKO mouse embryos by the direct transduction of (sgRNAs) for *Gfra1/2* and Cas9 mRNA into zygotes (Figures 6A–6C and S7A–S7D) (Wang et al., 2013). At first, we assayed the embryos at E8.5 just after heart tube formation, since embryonic defects or lethality would preclude the analysis of later stage embryos. Embryos containing multiples of three base insertions/deletions inside the exon were discarded from the analysis because the presence of functional protein production could not be refuted (Figure 6A). We examined cardiomyocyte differentiation of *Gfra1/2* DKO embryos by WISH using a marker of differentiated cardiomyocytes, *Nppa* (also known as *ANF*) (Bruneau et al., 2001; Christoffels et al., 2000). As expected from our ESC experiments, *Gfra1/2* DKO embryos exhibited a significant reduction of *Nppa* (Figure 6B), suggesting the cardiomyocyte differentiation process was significantly affected. As expected from the data of *Gfra2* KO ESCs (Figure 5D), *Gfra2* single-KO embryos showed significant but transiently elevated *Gfra1* expression by E8.5 in the heart field (Figure S7A). However, despite the fact that cardiomyocyte differentiation was impaired, the macro- and micro-anatomical morphology of the formed heart tube appeared unaffected in any DKO embryos (Figures 6B and S7B). This suggests that the reduction of *Nppa* in *Gfra1/2* DKO embryos simply reflected a delay of cardiomyocyte differentiation. To clarify this, we analyzed the *Gfra1/2* DKO embryos at E17.5 (Figures 6C, S7C, and S7D). Surprisingly, DKO embryos had the capacity to develop up to this stage without edema, and developed hearts were also observed (Figure 6C). This indicates that sufficient cardiomyocyte differentiation occurred in DKO embryos to support the fetal circulation. Further histological examination

OPEN ACCESS
CellPress

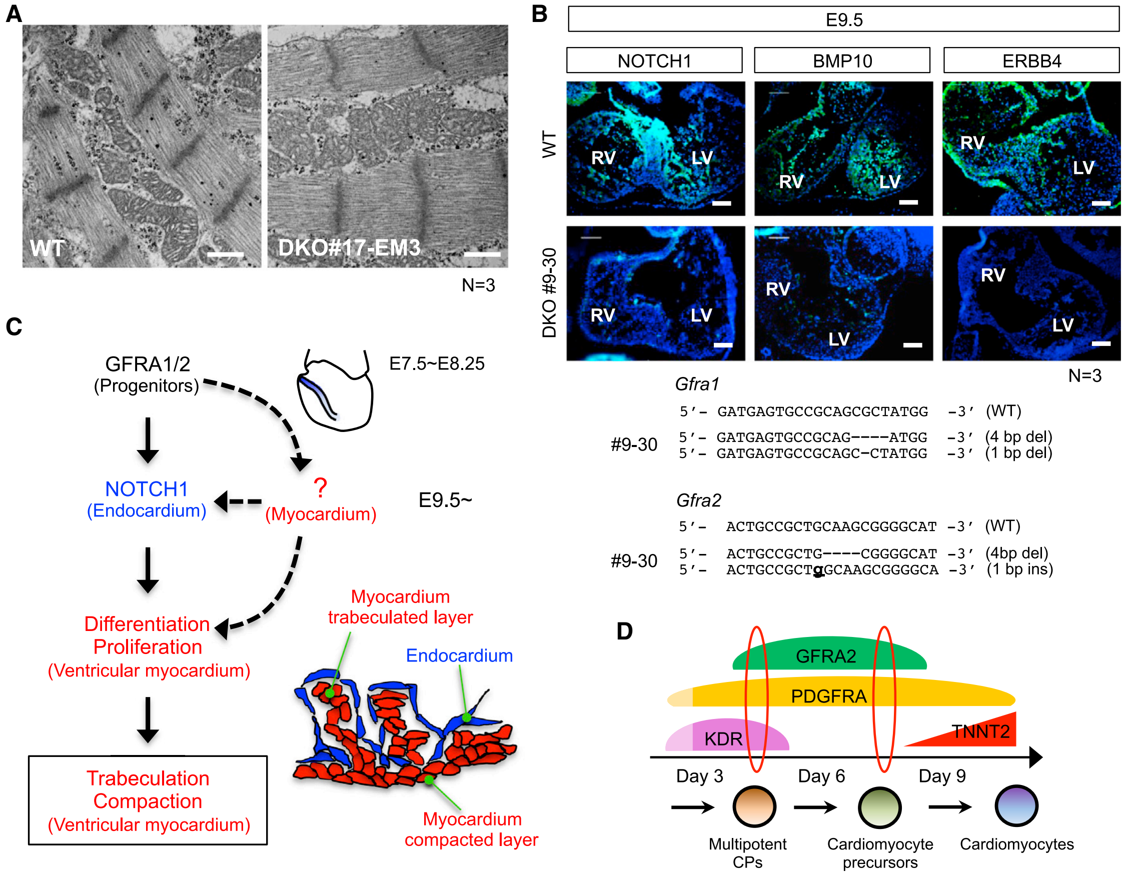

Figure 7. Impaired NOTCH Signaling in *Gfra1/2* DKO Embryos Is Responsible for Noncompaction Cardiomyopathy

(A) The unaltered structure of sarcomeres and mitochondria in the hearts of *Gfra1/2* DKO embryos. The data represent biological triplicates. Scale bar, 500 nm.

(B) Downregulation of NOTCH1, BMP10, and ERBB4 in E9.5 DKO hearts (green). The data represent biological triplicates. Blue, DAPI; LV, left ventricle; RV, right ventricle.

(C) Schematic model of the in vivo function of non-canonical *Gfra1/2* signal pathway.

(D) A model of the expression pattern of GFRA2 and other surface markers during differentiation of mouse and human pluripotent stem cells. At an earlier stage of differentiation, $GFRA2^{+}/KDR^{low+}/PDGFRA^{+}$ marks multipotent cardiovascular progenitors, whereas $GFRA2^{+}/KDR^{-}/PDGFRA^{+}$ marks cardiac precursors that are committed to cardiomyocytes fate at the later phase.

See also Figure S7.

unexpectedly revealed that *Gfra1/2* DKO hearts at E17.5 suffered from noncompaction cardiomyopathy (Figures 6C and S7D). Excessively prominent trabeculations and deep intra-trabecular recesses, which are characteristic features of noncompaction cardiomyopathy, were apparent, but no other congenital heart disease in DKO embryos was observed. However, the structure of sarcomeres and mitochondria were not altered (Figures 7A and S7C), suggesting that noncompaction was not caused by the abnormality of sarcomeres and mitochondria (Towbin et al., 2015). The absence of *Gfra1/2* and *Nrtn* at the sites of trabeculation and compaction suggests that RET-dependent and -independent GFRA1/2 signal pathways do not directly or locally regulate trabeculation and compaction (Figures S7A, S7E, and S7F). Importantly, we found that NOTCH1 in total protein and its downstream molecules BMP10 and ERBB4 were missing in DKO hearts at E9.5 (Figure 7B). NOTCH signaling is essential for proliferation and differentiation of ventricular cardiomyocytes through which proper trabeculated and compacted myocardial layers are formed, and mutants with NOTCH signaling defects exhibit a noncompaction phenotype (de la Pompa and Epstein, 2012; Grego-Bessa et al., 2007; Luxán et al., 2013; Zhang et al., 2013). Thus, the ventricular noncompaction observed in DKO embryos likely resulted from altered NOTCH signaling (Figures 7B and 7C).

Taken together, these results suggest that GFRA1/2 plays an important function in normal mammalian heart development, especially for ventricular wall compaction, but an unknown mechanism could compensate for cardiomyocyte differentiation due to the lack of GFRA1/2 in vivo. Thus, taken together, our data reveal a non-canonical signal cascade via GFRA1/2 is indispensable for heart development in vivo.

DISCUSSION

Here, we report a surface marker, GFRA2, that is specific for CPs in mouse and human. We show that the expression of

Gfra2 is initiated among both FHF and SHF CPs in vivo and in vitro just before the initiation of spontaneous beating of cardiomyocytes. *Gfra2* is downregulated after CPs terminally differentiate to cardiomyocytes. The use of an antibody specific for GFRA2 protein made it possible for us to harvest human and mouse CPs derived from pluripotent stem cells. Physiologically, *Gfra2* plays an important role in heart development in vitro as well as in vivo, but, in the absence of *Gfra2*, ectopic activation of *Gfra1* can functionally compensate for its loss. Of note, our data suggest that an alternative non-canonical signaling cascade transmits GFRA1/2 activation to CP function, and that this is distinct from the canonical signaling pathway dependent on RET.

In differentiating ESCs, it is known that $KDR^{low+}/PDGFRA^{+}$ or KDR^{low+}/KIT^{neg} cells constitute multipotent CPs which give rise to cardiomyocytes, smooth muscle cells, and endothelial cells, based on previously reported clonal tracing experiments (Bondue et al., 2011; Kattman et al., 2006, 2011; Yang et al., 2008). However, it remains an open question as to whether all of these cells or only a proportion of these cells are CPs. In this study, we found that almost all mouse $KDR^{low+}/PDGFRA^{+}$ express GFRA2 (Figure 3). However, the situation is likely somewhat different in human. The proportion of hGFRA2-expressing cells among $hKDR^{low+}/hPDGFRA^{+}$ population is much lower (Figure 4F). As expected, $hKDR^{low+}/hPDGFRA^{+}$ cells are negative for KIT (Yang et al., 2008). Of note, most GFRA2-negative $hKDR^{low+}/hPDGFRA^{+}$ cells failed to differentiate to cardiac cells (Figure S3E), which strongly supports the specificity of hGFRA2 for human CPs. Thus, in humans, the additional usage of hGFRA2 labeling is superior to the previously proposed protocols to isolate multipotent CPs with high purity. Furthermore, the use of GFRA2 labeling in addition to KDR and PDGFRA will enrich for more mature unipotent CPs which cannot be isolated with previous protocols dependent on KDR expression. It is interesting that these unipotent late-stage $GFRA2^{+}$ CPs represent not only the FHF, represented by HCN4/TBX5 and already known as unipotent, but also the SHF identified through expression of ISL1, because the SHF CPs are generally thought as multipotent (Figures 2G, 4E, S2D, and S2E) (Devine et al., 2014; Evans et al., 2010; Kelly and Evans, 2010; Kokkinopoulos et al., 2015; Lescroart et al., 2014; Später et al., 2013). This evidence indicates that the late-stage expression of GFRA2 excludes the multipotent SHF but includes the already committed but not fully differentiated SHF lineage. Thus, here we propose a strategy to isolate stage-specific human and mouse CPs with GFRA2, PDGFRA, and KDR (Figure 7D). $GFRA2^{+}/KDR^{low+}/PDGFRA^{+}$ triple-positive CPs would be multipotent cardiovascular progenitors. As CPs begin to commit but not yet terminally differentiate to cardiomyocytes, they lose KDR expression so that a $GFRA2^{+}/KDR^{neg}/PDGFRA^{+}$ double-positive population represents a cardiomyocyte precursor at a later stage.

We found that *Gfra1/2* DKO mouse ESCs showed a severe impairment of cardiomyocyte differentiation. Our data reveal that GFRA2 plays a pivotal role in cardiomyocyte differentiation in vitro that can be compensated by upregulation of GFRA1. Examples of an ectopic upregulation of an evolutionally duplicated paralogous gene to compensate for loss of a gene have been described (Barbaric et al., 2007). An evolutionally close relationship between *Gfra1* and *Gfra2* suggests that a preserved common enhancer drives *Gfra1* if *Gfra2* is not expressed in CPs, whereas a high level of *Gfra2* primarily suppresses *Gfra1* (Barbaric et al., 2007; Hätinen et al., 2007). However, the phenotype of KO of the known ligands for GFRA1 and 2, *Gdnf* and *Nrtn*, or KO of its co-receptor *Ret* did not show any defect of cardiomyocyte differentiation (Figures 5, S4, and S5). Interestingly, we observed a tendency of a slightly reduced yield of cardiomyocytes in single KO of *Gfra1*, *Gfra2*, and *Ret*, although not to a statistically significant degree (Figure 5B). Given the evidence that previously reported KO mice of each gene also did not show any heart defect (Enomoto et al., 1998; Heuckeroth et al., 1999; Rossi et al., 1999; Sánchez et al., 1996), the role of classic RET-dependent GFRA1/2 signals are unlikely to be vital for heart development. In addition, whereas an alternative GFRA1 signal pathway via NCAM1/FYN/FAK is known (Paratcha et al., 2003; Sjöstrand et al., 2007), our data showed that NCAM1, FYN, and FAK were not involved in the cardiac differentiation defect in the *Gfra1/2* DKO (Figure S6). Thus, the pathway by which GFRA2 modulates heart development is likely to be distinct from previously established pathways. To uncover the nature of this alternative non-canonical signaling pathway acting via GFRA1/2, further investigation is required, to identify molecules interacting directly with GFRA2 in the context of cardiac differentiation.

In contrast to the in vitro phenotype, the loss-of-function of *Gfra1/2* showed a different phenotype in vivo. In E8.5 DKO embryos, *Nppa* expression disappeared, which is likely consistent with the impaired in vitro cardiomyocyte differentiation of DKO ESCs. However, viable E17.5 DKO embryos possessing a developed heart were observed, indicating that cardiomyocyte differentiation itself occurred to form a functional fetal heart in vivo. Indeed, although missing *Nppa*, the heart tube of E8.5 DKO embryos seemed morphologically normal (Figures 6B and S7B). This evidence is inconsistent with the in vitro phenotype of ablated cardiomyocyte differentiation in DKO ESCs. Thus, the disappearance of *Nppa* in E8.5 DKO embryos might simply represent the delay of cardiomyocyte differentiation or anomalous sequential events inside the cardiomyocytes in DKO embryos. We propose that an unknown compensatory mechanism functions in vivo to circumvent the lack of GFRA1/2 to promote cardiomyocyte differentiation. Although the differentiation protocol for ESCs used in this study provides a strong drive toward cardiomyocytes with a defined set of growth factors (Kattman et al., 2011; Wamstad et al., 2012), these conditions are probably insufficient to perfectly mimic the in vivo environment for cardiomyocyte differentiation.

E17.5 DKO embryos showed noncompaction cardiomyopathy without other congenital heart diseases (Towbin et al., 2015; Zhang et al., 2013). We speculate that these DKO mice would not survive after birth because of the extremely thin compact layer of the ventricular wall. However, absence of edema in E17.5 DKO embryos suggests that the contractile apparatus in DKO cardiomyocytes at least developed sufficiently to support the fetal circulation. Unfortunately, germline deletion of *Gfra1* does not allow neonates to survive due to kidney agenesis (Figure 6C) (Enomoto et al., 1998; Sánchez et al., 1996). Thus, to confirm the perinatal prognosis, a conditional knockout will be required.

Importantly, we found that NOTCH1 and its downstream targets, BMP10 and ERBB4, were significantly downregulated in DKO embryos, and that this might be responsible for the noncompaction defect (Figures 7B and 7C) (Grego-Bessa et al., 2007; Luxán et al., 2013; Zhang et al., 2013). Given the lack of *Nppa* expression in NOTCH signal mutants (Luxán et al., 2013), it is concluded that the altered NOTCH signal in the endocardium promotes the downregulation of *Nppa* in DKO embryos. Surprisingly, *Gfra2* is expressed by CPs, but not at the sites of trabeculation or compaction (Figures 1, S1, S7E, and S7F). Thus, the defect within the endocardium is cell autonomously induced by the loss of *Gfra1/2* in multipotent CPs and is likely primarily required for ventricular noncompaction, although we cannot exclude a possibility that additional defects within the myocardium of DKO embryos may also contribute to this phenotype (Figure 7C).

Taken together, an alternative signaling pathway via GFRA1/2 is indispensable for proper heart development, although the mechanism underlying the signaling pathway of cardiac differentiation mediated by GFRA2 has yet to be elucidated. Future work, involving the clarification of the mechanistic details of this signaling pathway should provide deeper insight into cardiomyocyte differentiation, the biology of CPs, normal trabeculation, and compaction of ventricular myocytes.

EXPERIMENTAL PROCEDURES

The details are given in the Supplemental Experimental Procedures.

Animals

All animal procedures in this project were carried out under the project licenses 70/7254 and 80/2452 and 27-028-001 approved by the Home Office according to the Animals (Scientific Procedures) Act 1986 in the UK and Osaka University Animal Ethical Committee in Japan, respectively.

Cell Culture and Differentiation

Cardiomyocyte induction for mouse E14tg2a ESCs (Magin et al., 1992; Smith and Hooper, 1987) was performed according to standard protocols as previously described (Kattman et al., 2011). Cardiomyocyte induction from human HUES7 ESCs or iPSCs was undertaken as previously described (Burridge et al., 2011). Human ESCs were used under the license of the UK Steering Committee (reference number; SCSC13-25). Human iPSCs (iPS-HS1M) were originally established by D.M. using human dermal fibroblasts (HDFs) from a healthy donor under informed consent (Health Research Authority approval 13/LO/0224), for a study to be described elsewhere (D.M., T. McKay, L.D., and A.T., unpublished data).

CRISPR/Cas9-Mediated Genome Editing

The CRISPR/Cas9-mediated genome editing for mouse ESCs and embryos was performed as previously described (Hashimoto and Takemoto, 2015; Wang et al., 2013).

Flow Cytometry/FACS

Cells were sorted as previously described using FACS ARIA II or analyzed by LSR Fortessa II or FACSConto II (BD Biosciences) with FACSDiva 7.0 software (Kokkinopoulos et al., 2015).

SUPPLEMENTAL INFORMATION

Supplemental Information includes Supplemental Experimental Procedures, seven figures, and two movies and can be found with this article online at http://dx.doi.org/10.1016/j.celrep.2016.06.050.

AUTHOR CONTRIBUTIONS

K.Y. planned this project, and H.I. and K.Y. performed the majority of the experiments. K.Y. and H.I. wrote the manuscript. R.S., I.K., S.N., A-K.H., M.S., P.R., and K.O. contributed to mouse ESC experiments. A.P., D.M., S.H., L.D., A.T., and K.O. contributed to human ESCs and human iPSCs experiments. M.H. and H.S. contributed to CRISPR/Cas9-mediated direct genome editing. O.Y. and Y. Sakata contributed to electron microscopy observations. S.K., A-K.H., Y. Sawa, K.O., and K.S. contributed to the discussion of the results.

ACKNOWLEDGMENTS

This work was supported by the MRC New Investigator Research grant G0900105 and MRC Research grant MR/J007625/1 to K.Y., Japan Heart Foundation/Bayer Yakuhin Research Grant Abroad and JSPS (Japan Society for the Promotion of Science) Postdoctoral Fellowship for Research Abroad to H.I., and NIHR-funded Barts CVBRU. We thank Marika Charalambous and Kinya Otsu for helpful discussions; Hiroshi Hamada, Jinsuke Nishino, Denise Duboule, John Cobb, and Tristan McKay for sharing materials; and Timothy Mohun, Sophie Wood, and Sarah Johnson for help with microinjection of CRISPR/Cas9.

Received: February 11, 2016
Revised: May 22, 2016
Accepted: June 10, 2016
Published: July 7, 2016

REFERENCES

Airaksinen, M.S., and Saarma, M. (2002). The GDNF family: signalling, biological functions and therapeutic value. Nat. Rev. Neurosci. *3*, 383–394.

Baloh, R.H., Tansey, M.G., Johnson, E.M., Jr., and Milbrandt, J. (2000). Functional mapping of receptor specificity domains of glial cell line-derived neurotrophic factor (GDNF) family ligands and production of GFRalpha1 RET-specific agonists. J. Biol. Chem. *275*, 3412–3420.

Barbaric, I., Miller, G., and Dear, T.N. (2007). Appearances can be deceiving: phenotypes of knockout mice. Brief. Funct. Genomics Proteomics *6*, 91–103.

Bondue, A., Tännler, S., Chiapparo, G., Chabab, S., Ramialison, M., Paulissen, C., Beck, B., Harvey, R., and Blanpain, C. (2011). Defining the earliest step of cardiovascular progenitor specification during embryonic stem cell differentiation. J. Cell Biol. *192*, 751–765.

Brouilette, S., Kuersten, S., Mein, C., Bozek, M., Terry, A., Dias, K.R., Bhaw-Rosun, L., Shintani, Y., Coppen, S., Ikebe, C., et al. (2012). A simple and novel method for RNA-seq library preparation of single cell cDNA analysis by hyperactive Tn5 transposase. Dev. Dyn. *241*, 1584–1590.

Bruneau, B.G., Nemer, G., Schmitt, J.P., Charron, F., Robitaille, L., Caron, S., Conner, D.A., Gessler, M., Nemer, M., Seidman, C.E., and Seidman, J.G. (2001). A murine model of Holt-Oram syndrome defines roles of the T-box transcription factor Tbx5 in cardiogenesis and disease. Cell *106*, 709–721.

Burridge, P.W., Thompson, S., Millrod, M.A., Weinberg, S., Yuan, X., Peters, A., Mahairaki, V., Koliatsos, V.E., Tung, L., and Zambidis, E.T. (2011). A universal system for highly efficient cardiac differentiation of human induced pluripotent stem cells that eliminates interline variability. PLoS ONE *6*, e18293.

Cai, C.L., Liang, X., Shi, Y., Chu, P.H., Pfaff, S.L., Chen, J., and Evans, S. (2003). Isl1 identifies a cardiac progenitor population that proliferates prior to differentiation and contributes a majority of cells to the heart. Dev. Cell *5*, 877–889.

Christoffels, V.M., Habets, P.E., Franco, D., Campione, M., de Jong, F., Lamers, W.H., Bao, Z.Z., Palmer, S., Biben, C., Harvey, R.P., and Moorman, A.F. (2000). Chamber formation and morphogenesis in the developing mammalian heart. Dev. Biol. *223*, 266–278.

Cong, L., Ran, F.A., Cox, D., Lin, S., Barretto, R., Habib, N., Hsu, P.D., Wu, X., Jiang, W., Marraffini, L.A., and Zhang, F. (2013). Multiplex genome engineering using CRISPR/Cas systems. Science *339*, 819–823.

de la Pompa, J.L., and Epstein, J.A. (2012). Coordinating tissue interactions: Notch signaling in cardiac development and disease. Dev. Cell *22*, 244–254.

Devine, W.P., Wythe, J.D., George, M., Koshiba-Takeuchi, K., and Bruneau, B.G. (2014). Early patterning and specification of cardiac progenitors in gastrulating mesoderm. eLife *3*, 3.

Downs, K.M., and Davies, T. (1993). Staging of gastrulating mouse embryos by morphological landmarks in the dissecting microscope. Development *118*, 1255–1266.

Enomoto, H., Araki, T., Jackman, A., Heuckeroth, R.O., Snider, W.D., Johnson, E.M., Jr., and Milbrandt, J. (1998). GFR alpha1-deficient mice have deficits in the enteric nervous system and kidneys. Neuron *21*, 317–324.

Evans, S.M., Yelon, D., Conlon, F.L., and Kirby, M.L. (2010). Myocardial lineage development. Circ. Res. *107*, 1428–1444.

Fu, Y., Foden, J.A., Khayter, C., Maeder, M.L., Reyon, D., Joung, J.K., and Sander, J.D. (2013). High-frequency off-target mutagenesis induced by CRISPR-Cas nucleases in human cells. Nat. Biotechnol. *31*, 822–826.

Golden, J.P., DeMaro, J.A., Osborne, P.A., Milbrandt, J., and Johnson, E.M., Jr. (1999). Expression of neurturin, GDNF, and GDNF family-receptor mRNA in the developing and mature mouse. Exp. Neurol. *158*, 504–528.

Grego-Bessa, J., Luna-Zurita, L., del Monte, G., Bolós, V., Melgar, P., Arandilla, A., Garratt, A.N., Zang, H., Mukouyama, Y.S., Chen, H., et al. (2007). Notch signaling is essential for ventricular chamber development. Dev. Cell *12*, 415–429.

Hakuno, D., Takahashi, T., Lammerding, J., and Lee, R.T. (2005). Focal adhesion kinase signaling regulates cardiogenesis of embryonic stem cells. J. Biol. Chem. *280*, 39534–39544.

Hashimoto, M., and Takemoto, T. (2015). Electroporation enables the efficient mRNA delivery into the mouse zygotes and facilitates CRISPR/Cas9-based genome editing. Sci. Rep. *5*, 11315.

Hätinen, T., Holm, L., and Airaksinen, M.S. (2007). Loss of neurturin in frog–comparative genomics study of GDNF family ligand-receptor pairs. Mol. Cell. Neurosci. *34*, 155–167.

Heuckeroth, R.O., Enomoto, H., Grider, J.R., Golden, J.P., Hanke, J.A., Jackman, A., Molliver, D.C., Bardgett, M.E., Snider, W.D., Johnson, E.M., Jr., and Milbrandt, J. (1999). Gene targeting reveals a critical role for neurturin in the development and maintenance of enteric, sensory, and parasympathetic neurons. Neuron *22*, 253–263.

Hidaka, K., Shirai, M., Lee, J.K., Wakayama, T., Kodama, I., Schneider, M.D., and Morisaki, T. (2010). The cellular prion protein identifies bipotential cardiomyogenic progenitors. Circ. Res. *106*, 111–119.

Hiltunen, J.O., Laurikainen, A., Airaksinen, M.S., and Saarma, M. (2000). GDNF family receptors in the embryonic and postnatal rat heart and reduced cholinergic innervation in mice hearts lacking ret or GFRalpha2. Dev. Dyn. *219*, 28–39.

Kataoka, H., Takakura, N., Nishikawa, S., Tsuchida, K., Kodama, H., Kunisada, T., Risau, W., Kita, T., and Nishikawa, S.I. (1997). Expressions of PDGF receptor alpha, c-Kit and Flk1 genes clustering in mouse chromosome 5 define distinct subsets of nascent mesodermal cells. Dev. Growth Differ. *39*, 729–740.

Kathiriya, I.S., Nora, E.P., and Bruneau, B.G. (2015). Investigating the transcriptional control of cardiovascular development. Circ. Res. *116*, 700–714.

Kattman, S.J., Huber, T.L., and Keller, G.M. (2006). Multipotent flk-1+ cardiovascular progenitor cells give rise to the cardiomyocyte, endothelial, and vascular smooth muscle lineages. Dev. Cell *11*, 723–732.

Kattman, S.J., Witty, A.D., Gagliardi, M., Dubois, N.C., Niapour, M., Hotta, A., Ellis, J., and Keller, G. (2011). Stage-specific optimization of activin/nodal and BMP signaling promotes cardiac differentiation of mouse and human pluripotent stem cell lines. Cell Stem Cell *8*, 228–240.

Kelly, R.G., and Evans, S.M. (2010). The Second Heart Field, First Edition, *Volume 1* (Academic Press, Elsevier).

Kinder, S.J., Tsang, T.E., Quinlan, G.A., Hadjantonakis, A.K., Nagy, A., and Tam, P.P. (1999). The orderly allocation of mesodermal cells to the extraembryonic structures and the anteroposterior axis during gastrulation of the mouse embryo. Development *126*, 4691–4701.

Kokkinopoulos, I., Ishida, H., Saba, R., Ruchaya, P., Cabrera, C., Struebig, M., Barnes, M., Terry, A., Kaneko, M., Shintani, Y., et al. (2015). Single-cell expression profiling reveals a dynamic state of cardiac precursor cells in the early mouse embryo. PLoS ONE *10*, e0140831.

Lescroart, F., Chabab, S., Lin, X., Rulands, S., Paulissen, C., Rodolosse, A., Auer, H., Achouri, Y., Dubois, C., Bondue, A., et al. (2014). Early lineage restriction in temporally distinct populations of Mesp1 progenitors during mammalian heart development. Nat. Cell Biol. *16*, 829–840.

Liang, X., Wang, G., Lin, L., Lowe, J., Zhang, Q., Bu, L., Chen, Y., Chen, J., Sun, Y., and Evans, S.M. (2013). HCN4 dynamically marks the first heart field and conduction system precursors. Circ. Res. *113*, 399–407.

Luxán, G., Casanova, J.C., Martínez-Poveda, B., Prados, B., D'Amato, G., MacGrogan, D., Gonzalez-Rajal, A., Dobarro, D., Torroja, C., Martinez, F., et al. (2013). Mutations in the NOTCH pathway regulator MIB1 cause left ventricular noncompaction cardiomyopathy. Nat. Med. *19*, 193–201.

Magin, T.M., McWhir, J., and Melton, D.W. (1992). A new mouse embryonic stem cell line with good germ line contribution and gene targeting frequency. Nucleic Acids Res. *20*, 3795–3796.

McGrath, K.E., Koniski, A.D., Maltby, K.M., McGann, J.K., and Palis, J. (1999). Embryonic expression and function of the chemokine SDF-1 and its receptor, CXCR4. Dev. Biol. *213*, 442–456.

Nelson, T.J., Faustino, R.S., Chiriac, A., Crespo-Diaz, R., Behfar, A., and Terzic, A. (2008). CXCR4+/FLK-1+ biomarkers select a cardiopoietic lineage from embryonic stem cells. Stem Cells *26*, 1464–1473.

Paige, S.L., Plonowska, K., Xu, A., and Wu, S.M. (2015). Molecular regulation of cardiomyocyte differentiation. Circ. Res. *116*, 341–353.

Paratcha, G., and Ledda, F. (2008). GDNF and GFRalpha: a versatile molecular complex for developing neurons. Trends Neurosci. *31*, 384–391.

Paratcha, G., Ledda, F., and Ibáñez, C.F. (2003). The neural cell adhesion molecule NCAM is an alternative signaling receptor for GDNF family ligands. Cell *113*, 867–879.

Rana, M.S., Christoffels, V.M., and Moorman, A.F. (2013). A molecular and genetic outline of cardiac morphogenesis. Acta Physiol. (Oxf.) *207*, 588–615.

Rossi, J., Luukko, K., Poteryaev, D., Laurikainen, A., Sun, Y.F., Laakso, T., Eerikäinen, S., Tuominen, R., Lakso, M., Rauvala, H., et al. (1999). Retarded growth and deficits in the enteric and parasympathetic nervous system in mice lacking GFR alpha2, a functional neurturin receptor. Neuron *22*, 243–252.

Rossi, J., Herzig, K.H., Võikar, V., Hiltunen, P.H., Segerstråle, M., and Airaksinen, M.S. (2003). Alimentary tract innervation deficits and dysfunction in mice lacking GDNF family receptor alpha2. J. Clin. Invest. *112*, 707–716.

Sánchez, M.P., Silos-Santiago, I., Frisén, J., He, B., Lira, S.A., and Barbacid, M. (1996). Renal agenesis and the absence of enteric neurons in mice lacking GDNF. Nature *382*, 70–73.

Schuchardt, A., D'Agati, V., Larsson-Blomberg, L., Costantini, F., and Pachnis, V. (1994). Defects in the kidney and enteric nervous system of mice lacking the tyrosine kinase receptor Ret. Nature *367*, 380–383.

Scott, R.P., and Ibanez, C.F. (2001). Determinants of ligand binding specificity in the glial cell line-derived neurotrophic factor family receptor alpha S. J. Biol. Chem. *276*, 1450–1458.

Sjöstrand, D., Carlsson, J., Paratcha, G., Persson, B., and Ibáñez, C.F. (2007). Disruption of the GDNF binding site in NCAM dissociates ligand binding and homophilic cell adhesion. J. Biol. Chem. *282*, 12734–12740.

Smith, A.G., and Hooper, M.L. (1987). Buffalo rat liver cells produce a diffusible activity which inhibits the differentiation of murine embryonal carcinoma and embryonic stem cells. Dev. Biol. *121*, 1–9.

Später, D., Abramczuk, M.K., Buac, K., Zangi, L., Stachel, M.W., Clarke, J., Sahara, M., Ludwig, A., and Chien, K.R. (2013). A HCN4+ cardiomyogenic progenitor derived from the first heart field and human pluripotent stem cells. Nat. Cell Biol. *15*, 1098–1106.

Stanley, E.G., Biben, C., Elefanty, A., Barnett, L., Koentgen, F., Robb, L., and Harvey, R.P. (2002). Efficient Cre-mediated deletion in cardiac progenitor cells conferred by a 3'UTR-ires-Cre allele of the homeobox gene Nkx2-5. Int. J. Dev. Biol. *46*, 431–439.

Towbin, J.A., Lorts, A., and Jefferies, J.L. (2015). Left ventricular non-compaction cardiomyopathy. Lancet *386*, 813–825.

Wamstad, J.A., Alexander, J.M., Truty, R.M., Shrikumar, A., Li, F., Eilertson, K.E., Ding, H., Wylie, J.N., Pico, A.R., Capra, J.A., et al. (2012). Dynamic and coordinated epigenetic regulation of developmental transitions in the cardiac lineage. Cell *151*, 206–220.

Wang, H., Yang, H., Shivalila, C.S., Dawlaty, M.M., Cheng, A.W., Zhang, F., and Jaenisch, R. (2013). One-step generation of mice carrying mutations in multiple genes by CRISPR/Cas-mediated genome engineering. Cell *153*, 910–918.

Yang, L., Soonpaa, M.H., Adler, E.D., Roepke, T.K., Kattman, S.J., Kennedy, M., Henckaerts, E., Bonham, K., Abbott, G.W., Linden, R.M., et al. (2008). Human cardiovascular progenitor cells develop from a KDR+ embryonic-stem-cell-derived population. Nature *453*, 524–528.

Zhang, W., Chen, H., Qu, X., Chang, C.P., and Shou, W. (2013). Molecular mechanism of ventricular trabeculation/compaction and the pathogenesis of the left ventricular noncompaction cardiomyopathy (LVNC). Am. J. Med. Genet. C. Semin. Med. Genet. *163C*, 144–156.

Cell Reports, Volume *16*

Supplemental Information

GFRA2 Identifies Cardiac Progenitors

and Mediates Cardiomyocyte Differentiation

in a RET-Independent Signaling Pathway

Hidekazu Ishida, Rie Saba, Ioannis Kokkinopoulos, Masakazu Hashimoto, Osamu Yamaguchi, Sonja Nowotschin, Manabu Shiraishi, Prashant Ruchaya, Duncan Miller, Stephen Harmer, Ariel Poliandri, Shigetoyo Kogaki, Yasushi Sakata, Leo Dunkel, Andrew Tinker, Anna-Katerina Hadjantonakis, Yoshiki Sawa, Hiroshi Sasaki, Keiichi Ozono, Ken Suzuki, and Kenta Yashiro

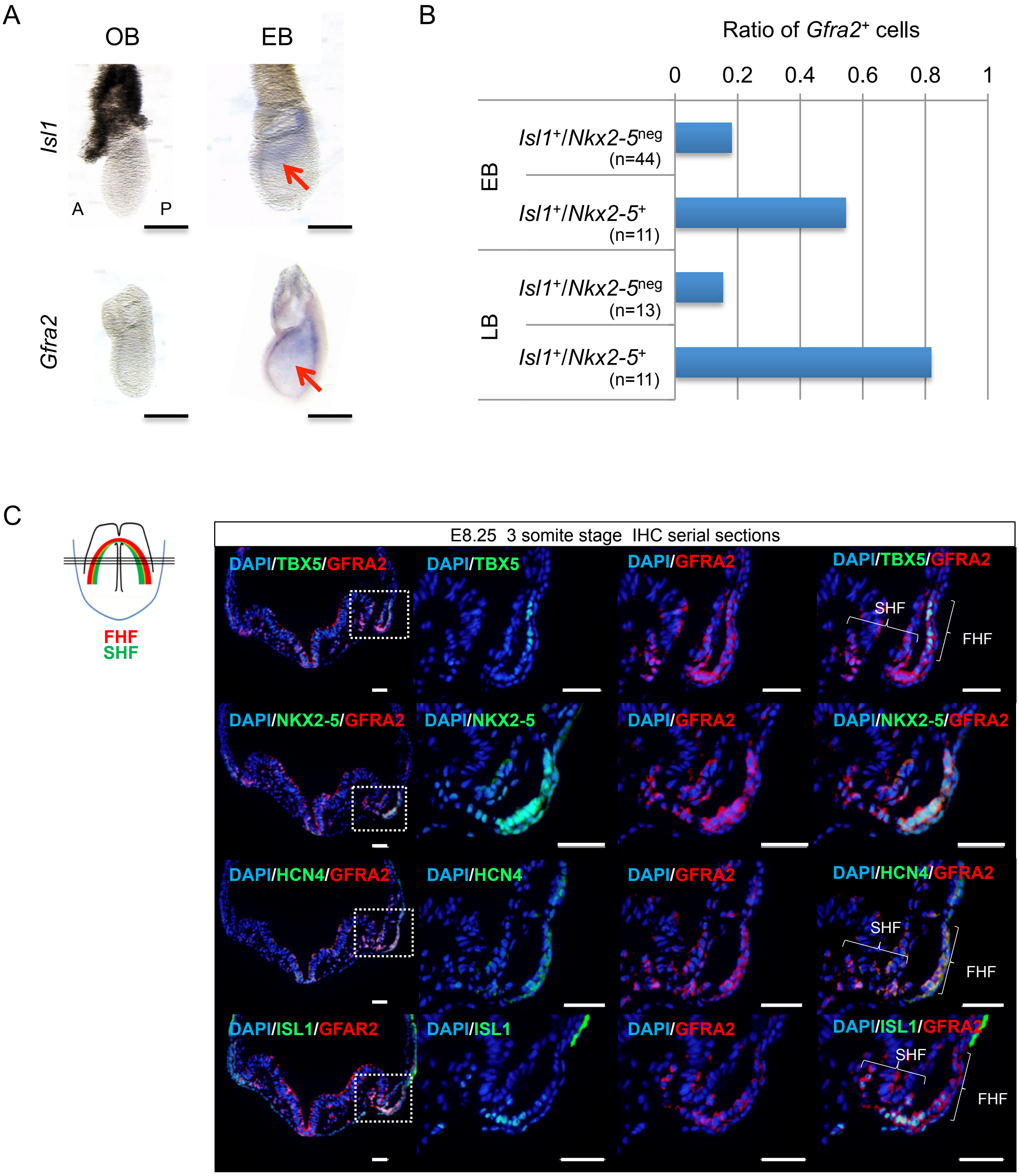

Figure S1. Related to Figure 1. **The distribution of GFRA2 expression in the mouse embryo.** (A) Whole mount in situ hybridization (WISH) for *Isl1* and *Gfra2* on No Bud (OB) stage and Early allantoic Bud (EB) stage embryos. The embryos are indicated as the left lateral view. The data are representative of biological duplicates and triplicates for OB stage and EB stage, respectively. For *Gfra2* at the EB stage, the indicated data is the same as in Fig. 1A. Note both genes were clearly visible at the EB stage but not at the OB stage. A; anterior, P; posterior, Scale bar; 250 µm. (B) The ratio of *Gfra2*$^{+}$ single cell cDNAs among those of *Isl1*$^{+}$/*Nkx2-5*^{neg} and of *Isl1*$^{+}$/*Nkx2-5*$^{+}$. LB; Late allantoic Bud stage. (C) Immunohistochemistry of GFRA2, TBX5, NKX2-5, ISL1, and HCN4 on serial sections of the three-somite stage embryo. GFRA2^{+} cells are NKX2-5^{+} CPs and the sum of TBX5^{+}/HCN4^{+} FHF and ISL1^{+} SHF CPs. FHF, first heart field; SHF, second heart field. Scale bar; 50µm. Micrograph represents biological triplicates.

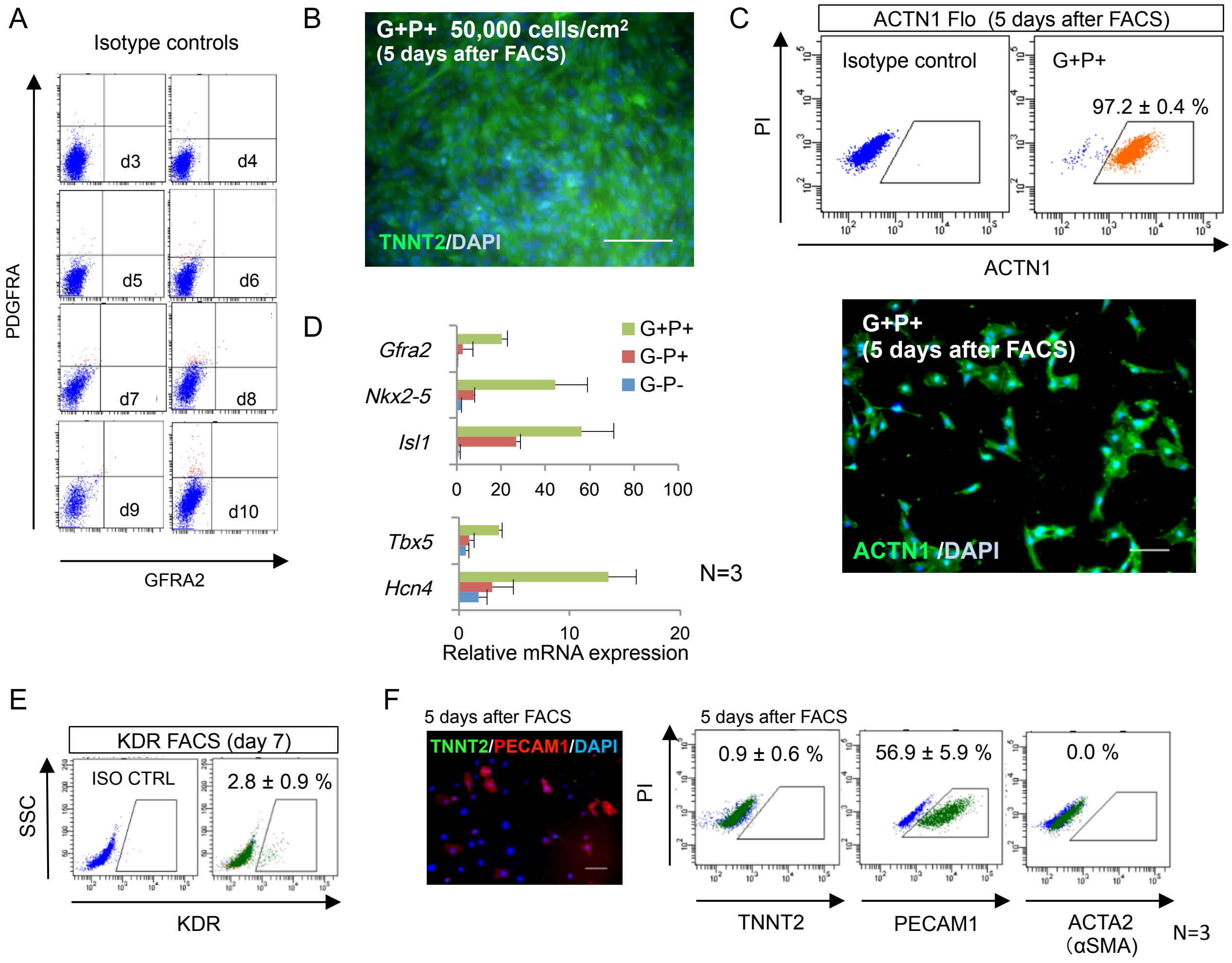

Figure S2. Related to Figure 2 and 3. **Biological features of GFRA2^{+} CPs from mouse ESCs.**
(A) Isotype controls of serial differentiation stages of mouse ESCs for Figure 2A. (B) Immunofluorescence of TNNT2 in FACS-isolated GFRA2^{+}/PDGFRA^{+} (G+P+) cells 5 days after FACS. Cells were seeded at higher density (50,000 cells/cm^{2}). Note almost all cells show TNNT2 positive and spontaneous synchronized beating (See Movie S1). Cells could expand more efficiently when seeded at higher density. Scale bar; 100 µm. (C) Immunocytochemistry and flow cytometry (Flo) for another cardiomyocyte marker ACTN1 (also known as ACTININ α1). GFRA2^{+}/PDGFRA^{+} cells were isolated by FACS at day 7 and differentiated for a further 5 days. Most of the cells (97.2 ± 0.4%) were ACTN1 positive cardiomyocytes. N=3. (D) qPCR analyses of *Gfra2*, *Nkx2-5*, *Isl1*, *Tbx5*, and *Hcn4* in FACS-isolated G+P+, G–P+, G–P– cells at day 7. Expression levels of all cardiac genes were significantly higher in the G+P+ population when compared to G–P+ and G–P– population (*$P < 0.05$, Student t-test). Data are representative of biological triplicates with technical duplicates as mean ± s.e.m.. (E) Flow cytometrical analysis of KDR in day 7 differentiating ESCs. (F) Differentiation fate of FACS-purified KDR^{+} cells. KDR^{+} cells were isolated at day 7 and kept in culture for 5 days. Immunocytochemical and flow cytometrical analyses show KDR^{+} cells at day 7 efficiently differentiated into endothelial cells. Scale bar; 100 µm.

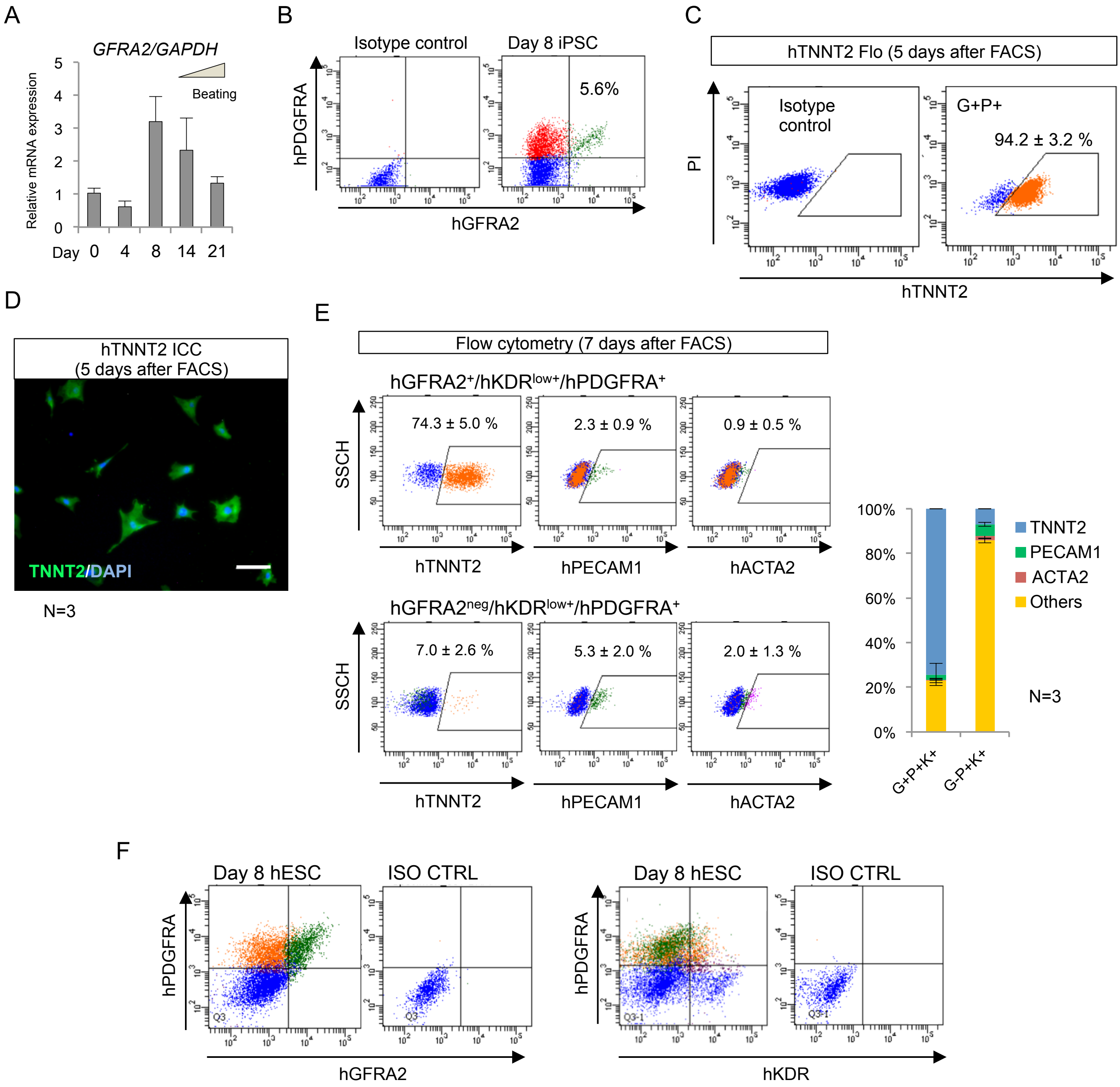

Figure S3. Related to Figure 4. **hGFRA2⁺ CPs derived from human iPSCs.**
(A) qPCR analysis for human *GFRA2* during cardiac differentiation of human iPSCs. A transient expression peak of human *GFRA2* was observed at day 8, similar to mouse and human ESCs. Bar graph represents biological triplicates with technical duplicates as mean ± s.e.m.. (B) Flow cytometrical analysis of hGFRA2 and hPDGFRA on differentiation day 8 human iPSCs. Similar to mouse and human ESCs, hGFRA2^{+}/hPDGFRA^{+} CP population could be clearly seen in cardiac differentiating iPSCs. (C, D) FACS-isolated hGFRA2^{+}/hPDGFRA^{+} CPs from human iPSCs efficiently differentiated into TNNT2^{+} cardiomyocytes. Scale bar; 100 µm. (E) Flow cytometrical analyses for hTNNT2 (cardiomyocytes), hPECAM1 (endothelial cells), and hACTA2 (smooth muscle cells; also known as αSMA) 7 days after the FACS-isolation of hGFRA2^{+}/hKDR^{low+}/hPDGFRA^{+}/hKIT^{neg} and hGFRA2^{neg}/hKDR^{low+}/hPDGFRA^{+}/hKIT^{neg} population. Note hGFRA2 negative population almost lost cardiac differentiation ability. Bar graph represents mean ± s.e.m. N=3. (F) Flow cytometry for hGFRA2, hPDGFRA, and hKDR at day 8 of human ESCs. hGFRA2^{+}/hPDGFRA^{+} CPs (shown as green dots) lost hKDR expression (right column) as compared with day 4 (Fig. 4f).

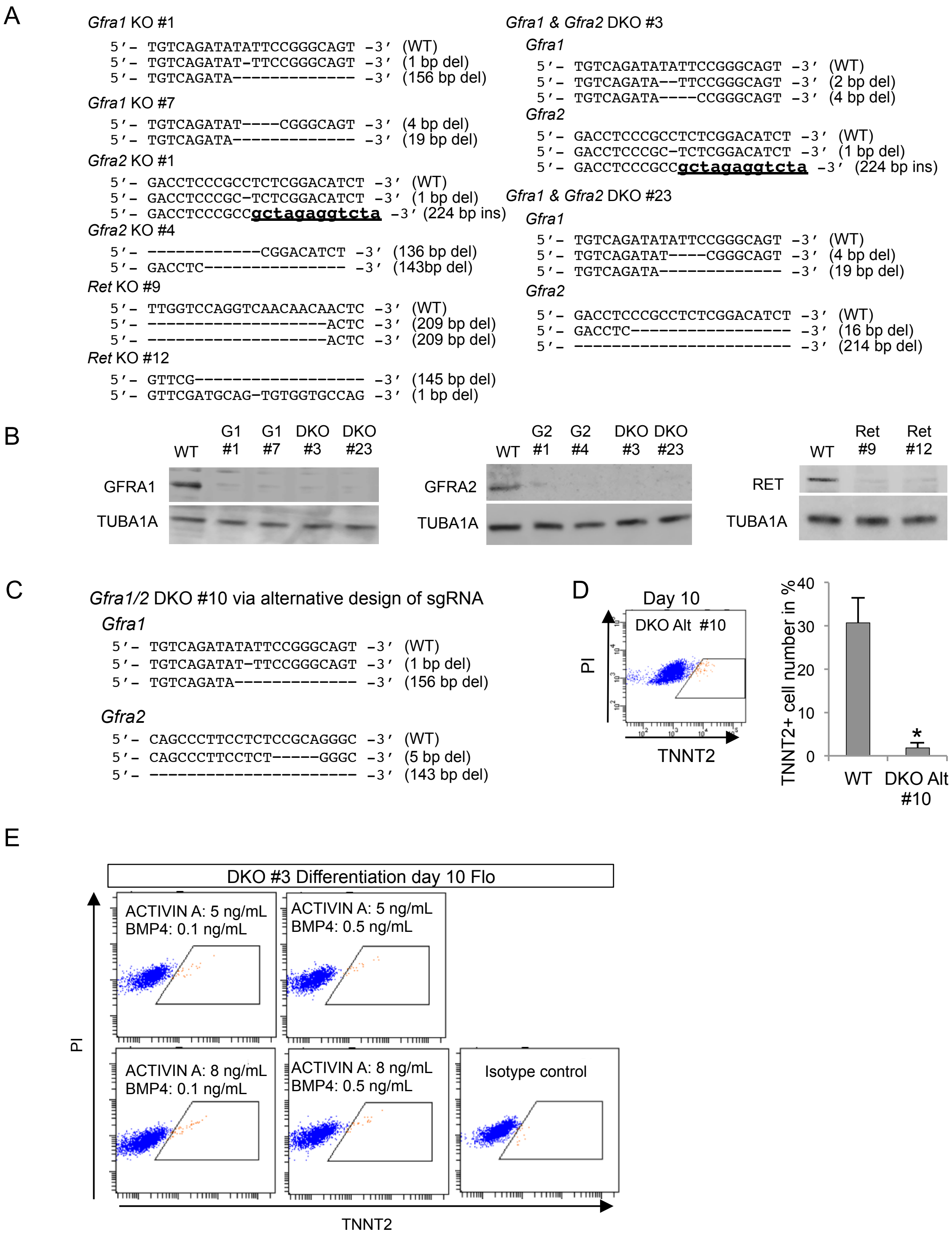

Figure S4. Related to Figure 5. ***Gfra1, Gfra2, Ret*****, and *****Gfra1/2* knockout mouse ES cell clones.**
(A) Genotyping results of each knockout (KO) ES cell line. In principle, all mutations cause premature termination of translation of proteins. (B) Immunoblotting images of each KO ES cell line revealing them to be protein nulls. (C) The genotype of DKO clone ID_#10 via another different design of sgRNA for *Gfra2.* Targeting was performed on *Gfra1* KO clone ID_#1. (D) An alternative DKO clone ID_#10 also exhibited a severe impairment in cardiomyocyte differentiation, suggesting that this cardiac defect is not caused by off-target mutations. (E) Flow cyometrical analyses for TNNT2 in differentiation day 10 indicates that the cardiac differentiation defect of DKO #3 ESCs was not improved by varied concentration of ACTIVIN A and BMP4.

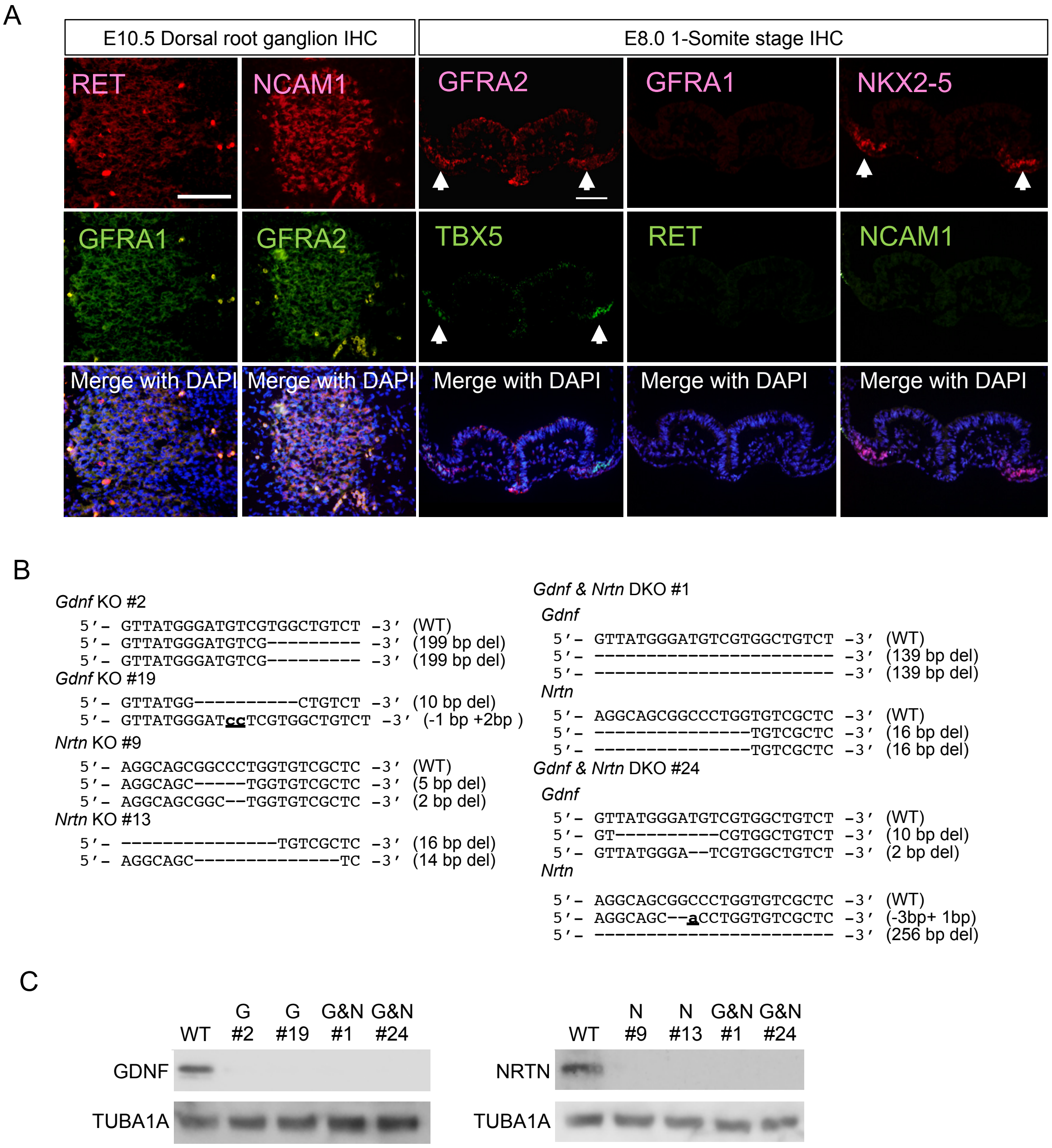

Figure S5. Related Figure 5. **Mouse ES cell clones of *Gdnf* KO, *Nrtn* KO, and *Gdnf/Nrtn* DKO.**
(A) Micrograph represents the reproduced immunofluorescence staining of GFRA1, GFRA2, RET, NKX2-5, TBX5, and NCAM1 from the 3-somite stage embryos (N=3). Note GFRA1, RET, and NCAM1 were not detected. White arrows indicate the heart field. The validity of used antibodies against GFRA1, RET, and NCAM1 is shown in the dorsal root ganglion of E10.5 mouse embryos. Scale bar; 100 µm. (B) Genotyping results for each *Gdnf*, *Nrtn,* and *Gdnf/Nrtn* KO ES cell line. All mutations caused premature termination of translation of proteins. (C) Immunoblotting images of each KO ES cell line confirmed them as protein nulls. G, *Gdnf*-KO. N, *Nrtn*-KO. G&N, *Gdnf/Nrtn*-DKO.

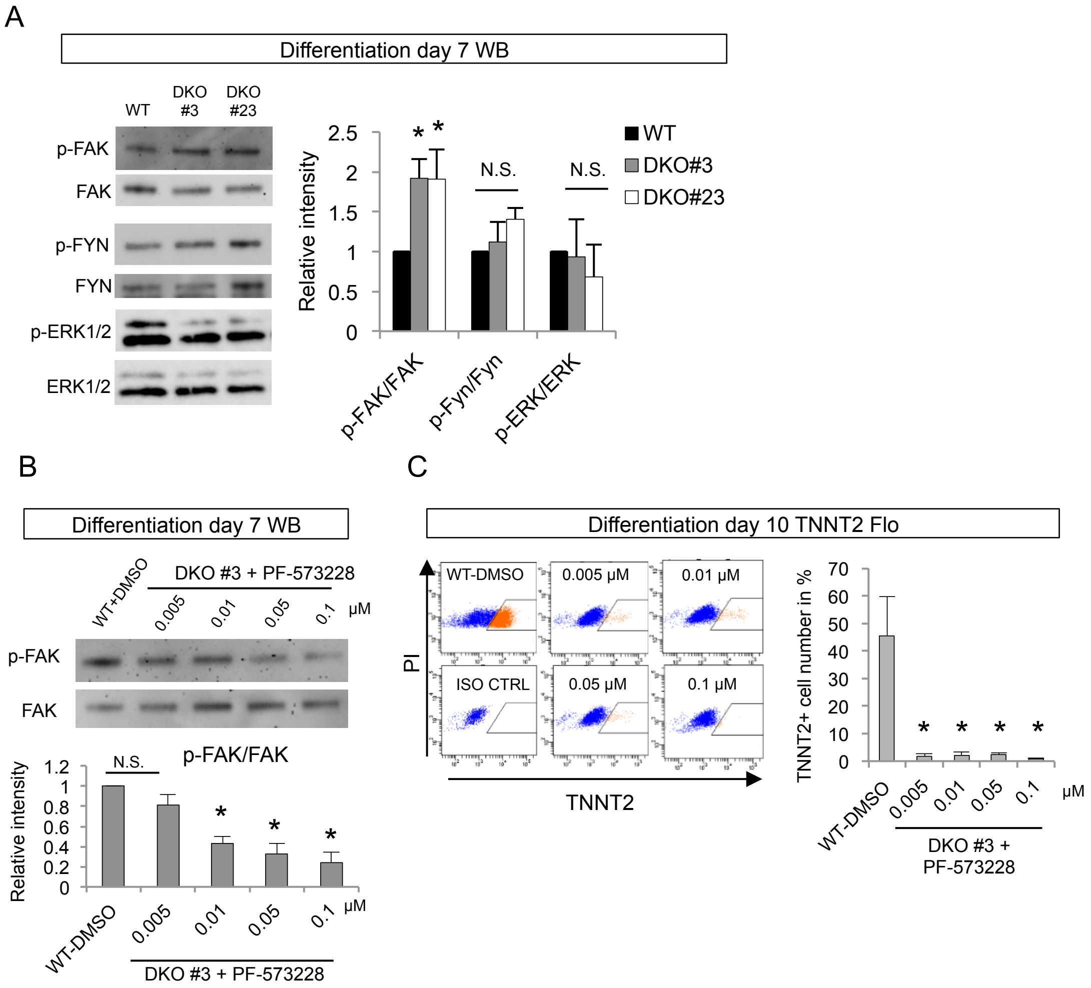

Figure S6. Related to Figure 5. **Signal pathway analyses in *Gfra1/2* DKO ESCs.**
(A) Western blotting (WB) analyses of phosphorylated FAK (p-FAK), FYN (p-FYN), and ERK1/2 (p-ERK1/2) at day 7 of differentiation in WT and DKO ESCs. p-FAK was significantly elevated in *Gfra1/2* DKO ES cell lines, whereas p-FYN and p-ERK1/2 were not significantly affected. **P* < 0.05 vs WT in *Student's* t-test. N.S., not significant. Bar graph represents mean ± s.e.m.. N=3. (B) Serial concentrations of FAK inhibitor PF-573228 were administered to differentiation media from day 5. Note that p-FAK in DKO ESCs was not significantly different from WT when 0.005 μM. **P* < 0.05 vs WT in *Student's* t-test. Bar graph represents mean ± s.e.m.. N=3. (C) Suppression of FAK phosphorylation failed to rescue cardiomyocyte differentiation defects in *Gfra1/2* DKO ESCs. **P* < 0.05 vs WT in *Student's* t-test. Bar graph represents mean ± s.e.m.. N=3.

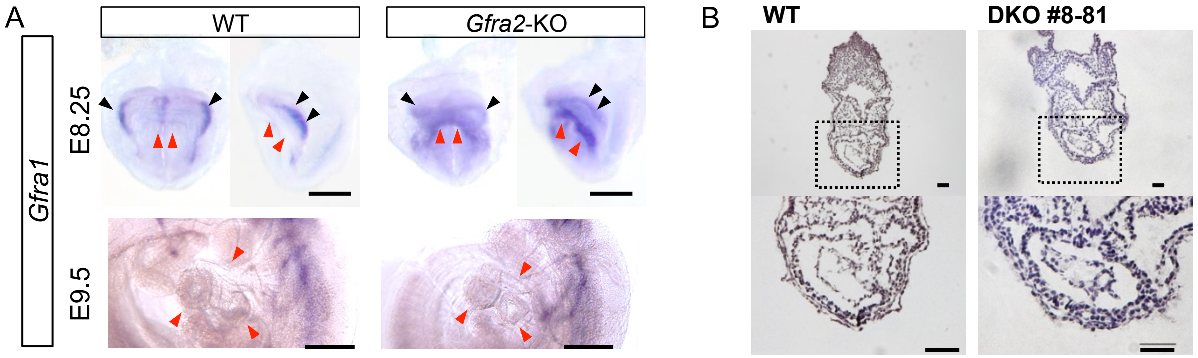

C

Gfra1

DKO ID	*Gfra1*	*Gfra2*
	5′- GATGAGTGCCGCAGCGCTATGG -3′ (WT)	5′- ACTGCCGCTGCAAGCGGGGCAT -3′ (WT)
#17-1	5′- -----------------TATGG -3′ (20 bp del)<br>5′- GATGAGTGCCGC-----TATGG -3′ (5 bp del)	5′- -----------------**gt**CAT -3′ (220 -> 2bp)<br>5′- -----------------**gt**CAT -3′ (220 -> 2bp)
#17-3	5′- GATGAGTGCCGC-----TATGG -3′ (5 bp del)<br>5′- GATGAGTGCCGC-----TATGG -3′ (5 bp del)	5′- ACTGCCGC--------GGGGCAT -3′ (7bp del)<br>5′- ACTGCCGCTGC**c**AAGCGGGGCAT -3′ (1 bp ins)
#17-4	5′- GATGAGTGCC--------TATGG -3′ (7 bp del)<br>5′- GATGAGTGCC--------TATGG -3′ (7 bp del)	5′- TCCTCTCCGCAGGGCTCTGAGC -3′ (WT)<br>5′- TCCTCTC----------TGAGC -3′ (10 bp del)<br>5′- TCCTCT-----------TGAGC -3′ (11 bp del)
#17-19 (Hetero)	5′- GATGAGTGCCGCAGCGCTATGG -3′ (WT)<br>5′- ----------------------- -3′ (176 bp del)	5′- ACTGCCGCTGCAAGCGGGGCAT -3′ (WT)<br>5′- ACTGCCGC--------GGGGCAT -3′ (7 bp del)
#17-EM3	5′- GATGAGTGCCGC-----TATGG -3′ (5 bp del)<br>5′- GATGAGTGCCGC-----TATGG -3′ (5 bp del)	5′- ACTGCCGC--------GGGGCAT -3′ (7bp del)<br>5′- ACTGCCGCTG----CGGGGCAT -3′ (4 bp del)

D

Genotype	Heart phenotype	Number
Gfra1 -/-	Normal	2*
	Noncompaction	0
Gfra2 -/-	Normal	2
	Noncompaction	0
Gfra1 +/-, *Gfra2* +/-	Normal	2
	Noncompaction	0
Gfra1 -/-, *Gfra2* +/-	Normal	4*
	Noncompaction	0
Gfra1 -/-, *Gfra2* -/-	Normal	0
	Noncompaction	3*

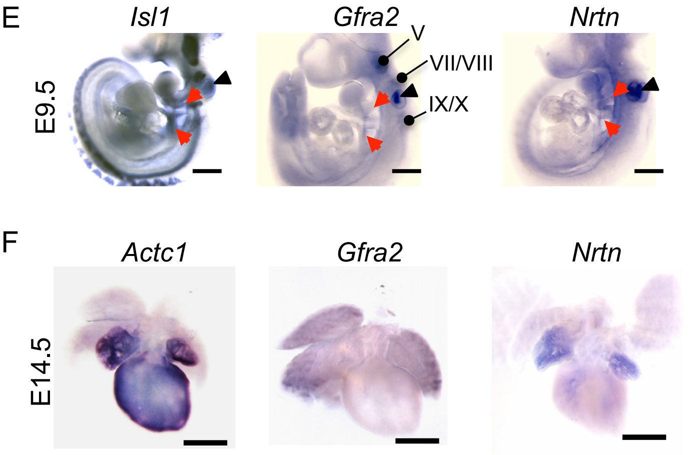

Figure S7. Related to Figure 6 and 7. **The phenotype of *Gfra1/2* DKO mouse embryos generated via CRISPR/Cas9 direct genome editing.**
(A) Transient ectopic *Gfra1* expression in *Gfra2* knockout embryos. *Gfra1* was expressed in the neural crest lineage (black arrowheads), but not in cardiac crescent (red arrowheads in the upper row) of WT embryo. By contrast, *Gfra1* was up-regulated in *Gfra2*-null embryos in the cardiac crescent at E8.25 (red arrowheads in the upper row of the right column) but not at the the heart region of E9.5 embryo (red arrowheads in the lower row). The heart of E9.5 is indicated as the left lateral view. Scale bar, 250 μm. (B) HE staining of the sections of E8.5 *Gfra1/2* DKO #81 mouse embryo and WT littermate. The morphological alteration of the heart tube was not noticed in DKO embryos, although *Nppa* disappeared (Fig. 6B). Scale bar; 50 μm. (C) Genotype of the E17.5 *Gfra1/2* DKO mouse embryos generated by CRISPR/Cas9 mediated direct genome editing. (D) Summary of the E17.5 mutants obtained via direct genome editing with CRISPR/Cas9. All the cases of *Gfra1* null showed kidney agenesis (asterisks). Note that only DKO embryos showed noncompaction. (E) WISH for *Isl1*, *Gfra2*, and *Nrtn* on WT mouse embryos at E9.5 when trabeculation of myocardium occurs. Note *Gfra2* and *Nrtn* were not expressed inside the heart, although we could not exclude that they were expressed in a part of SHF at the dorsal mesentery (red arrowheads) represented by *Isl1*. *Gfra2* and *Nrtn* was detected weakly at the pharyngeal pouch. The signal at otic vesicle is likely artifact (black arrowheads). The embryos are indicated as left lateral view. N=3. V; trigeminal nucleus, VII/VIII; facial/acoustic nucleus, IX/X; glossopharyngeal/vagus nucleus, Scale bar; 250 μm. (F) WISH for *Actc1* (a cardiomyocyte marker), *Gfra2*, and *Nrtn* on WT mouse embryonic hearts at E14.5 when myocardial compaction occurs. *Gfra2* was not expressed in the heart at E14.5 but was detected at alveoli in the lung. *Nrtn* was observed in the atrium and at the ventricular endocardium, but not in the ventricular myocardium layer. N=3. Scale bar; 750 μm.

Figure legends for Supplemental Movies

Movie S1. Related to Figure 2. **Spontaneously beating cardiomyocytes differentiated from GFRA2$^+$/PDGFRA$^+$ mouse cardiac progenitors after FACS isolation.**

After an additional 5 days culture in differentiation media, GFRA2$^+$/PDGFRA$^+$ cells showed synchronized spontaneous contraction, suggesting that they can effectively differentiate into functional cardiomyocytes.

Movie S2. Related to Figure 4. **Spontaneously beating cardiomyocytes derived from FACS-isolated hGFRA2$^+$/hPDGFRA$^+$ human cardiac progenitors.**

FACS-isolated hGFRA2$^+$/hPDGFRA$^+$ cells from human ESCs differentiated into spontaneously beating cardiomyocytes.

SUPPLEMENTAL EXPERIMENTAL PROCEDURES

Animals

All animal procedures in this project were carried out under the project licenses (70/7254 and 80/2452) and (27-028-001) approved by the Home Office according to the Animals (Scientific Procedures) Act 1986 in the UK and Osaka University Animal Ethical Committee in Japan, respectively.

Staging of mouse embryos

Developmental stages of mouse embryos were classified according to morphology as previously described (Downs and Davies, 1993). The morning of the day of vaginal plug detection was set as E0.5.

Single cell RNA-seq of embryonic cardiac progenitors

Deep sequencing and bioinformatics analyses on single-cell cDNAs were performed as previously described (Brouilette et al., 2012; Kokkinopoulos et al., 2015; Kurimoto et al., 2006). Sequences used to identify *Gfra2* have been submitted to NCBI Gene Expression Omnibus (GEO, http://www.ncbi.nlm.nih.gov/geo) under the accession numbers GSE38198 and GSE63796.

Whole mount in situ hybridization

All whole mount in situ hybridization procedures were performed as previously described (Uehara et al., 2009). A probe for *Gfra2* was kindly given by J. Cobb, D. Duboule and J. Nishino, for *Gfra1* by H. Hamada and J. Nishino, *Actc1* for R. Kelly and for *Nppa* by P. Riley. For *Nrtn*, cDNA clone of MGC:68236/IMAGE:5345262 was used for probe synthesis. Images were acquired with a Leica M205FA stereomicroscope using a DFC310 FX digital camera, or with Olympus SZX12 stereomicroscope using a DP70 digital camera.

Histology

Immunofluorescence on tissue sections were performed as previously described (Kokkinopoulos et al., 2015). Embryos were dissected and fixed with 4% paraformaldehyde (PFA) at 4 °C overnight. Then they were embedded in OCT compound and cryosections were prepared at 5 μm thickness. For immunohistochemistry, non-specific antibody binding sites were pre-blocked with 5% skimmed milk/PBS or 3% bovine serum albumin (BSA)/PBS for 30 min at room temperature. Primary antibodies used were; anti-ACTN1 (Sigma A7811, 1:200), anti-BMP10 (R&D MAB6038, 1:100), anti-ERBB4 (Santa Cruz, sc-284, 1:100), anti-GFRA2 (R&D AF429, 5 μg/mL), anti-HCN4 (Millipore AB5808, 1:100), anti-ISL1 (DSHB 39.4D5, 1:100), anti-NCAM1 (Abcam ab133345, 1:100), anti-NKX2-5 (Abcam ab35842, 1:100), anti-NOTCH1 (Cell Signaling Technology 4380, 1:100), and anti-TBX5 (SIGMA HPA8786, 1:100). Antigen retrieval was performed for the detection of NOTCH1 and BMP10 before primary antibody reaction, by incubation in 10 mM Citrate (pH 6.0) at a sub-boiling temperature for 10 min. To dilute a primary antibody, Can Get Signal Immunostain solution B (TOYOBO NKB-601) was used only for ERBB4. For secondary antibodies (all from Molecular Probes), donkey anti-goat Alexa 488 or 594 (1:400) and donkey anti-rabbit Alexa 488 (1:400) or donkey anti-mouse Alexa 488 (1:400) diluted with 5% skimmed milk/PBS or 3% BSA/PBS were applied for 90 min at room temperature. These secondary antibodies were also used for the immunocytochemistry. Nuclei were stained with 4',6-diamino-2-pheylindole (DAPI). The hearts of double knockout embryos were fixed with 4% PFA overnight and immersed in 30% Sucrose/PBS overnight. The sections were prepared at 10 μm thickness and stained with haematoxylin and eosin. Images were acquired with KEYENCE BZ8000 fluorescence or Zeiss LSM510 laser confocal microscopes.

Cell culture and differentiation

E14tg2a mouse ESCs (Magin et al., 1992; Smith and Hooper, 1987) were maintained under feeder-free conditions in ESGRO complete plus medium (Millipore) supplemented with mouse leukemia inhibitory factor (ESGRO, Millipore). Cardiomyocyte induction for mouse ESCs was performed according to standard protocols as previously described with some modifications (Kattman et al., 2011). Briefly, cells were plated onto non-coated petri dishes in 7,500 cells/mL in SFD medium (IMDM:Ham's F12, 3:1; N2 supplement; B27 supplement; 0.05% BSA; Pen/Strep). Two days later (day 2), floating embryoid bodies (EBs) were collected, trypsinised, and plated onto petri dishes in 7,500 cells/mL in SFD medium supplemented with human recombinant VEGF (R&D, 5 ng/mL), Activin A (R&D, 5 ng/mL), and BMP4 (R&D, 0.25 ng/mL). At day 4, floating EBs were collected, trypsinised, and plated on 0.1% gelatin-coated 96 well dishes in 150,000 cells/well in StemPro34

medium supplemented with human recombinant VEGF (R&D, 5 ng/mL), bFGF (R&D, 10 ng /mL), and FGF10 (R&D, 5 ng/mL). Spontaneously beating cardiomyocytes were typically seen at day 8. Human ES cell line HUES7 was maintained on Matrigel-coated tissue culture dishes with mTeSR1 medium (STEMCELL Technologies,) according to the manufacturer's instructions. Cardiomyocyte induction from human ESCs or iPSCs was undertaken using a modified version of a previously reported protocol (Burridge et al., 2011). Briefly, cells were dissociated with Accutase (Invitrogen) and plated on Matrigel-coated 25cm^2 flasks at 2,000,000 cells/flask with mTesR1 medium containing 5 μM ROCK inhibitor Y-27632 (day -1). The following day (day 0), cells were dissociated with Accutase and suspended with RIP medium (RPMI contained with 4mg/mL polyvinyl alcohol, chemically defined lipid, Insulin-Transferrin-Selenium (ITS), 400 μM 1-Thioglycerol (MTG), 20 ng/mL BMP4, 6 ng/mL bFGF, 1 μM Y-27632 ROCK inhibitor) and plated into V-bottom 96-well dishes in 8,000 cell/well, then centrifuged at 950g for 5 min. Two days later (day 2), media were carefully aspirated and replaced with RD medium (RPMI with 20% FBS). Following two days (day 4), EBs were carefully transferred into U-bottom 96 well dishes with RI medium (RPMI containing chemically defined lipid, ITS, and MTG). Beating EBs were typically seen at day 9 to 10. Differentiated cardiomyocytes were assayed at differentiation day 10, because usually spontaneous beating areas of differentiated cardiomyocytes plateaued at day 10. If CPs were isolated via cell sorting, the cardiomyocytes were assayed on day 12, because spontaneous beating areas of differentiated cardiomyocytes plateaued at day 11 or 12 in many cases. This delay of differentiation might be caused by the stress on CPs associated with cell dissociation, FACS, and/or the re-plating procedure. Human ESCs were used under the license of the UK Steering Committee (reference number; SCSC13-25). Human iPSCs (iPS-HS1M) were originally established by D. Miller using human dermal fibroblasts (HDFs) from a healthy donor under informed consent (Health Research Authority approval 13/LO/0224), for a study to be described elsewhere (Miller et al., manuscript in preparation). HDFs were reprogrammed using the single polycistronic lentivector hSTEMCCA (a kind gift from T. McKay), with reprogrammed colonies isolated, clonally expanded, and one resulting iPS cell line iPS-HS1M characterised as described elsewhere (Miller et al., manuscript in preparation) (Somers et al., 2010).

Flow cytometry/FACS

Cells were treated with 3mM EDTA for 3 min at 37 °C to dissociate into single cells. Then they were washed once with FACS buffer (HBSS with 3% FBS and 0.03mM EDTA) and centrifuged for 3 min at 1,000 rpm. Cells were suspended with the primary antibody solution containing anti-human/mouse GFRA2 (R&D, 15 μg/mL), anti-mouse PDGFRA-PE (Abcam, 1:100) or anti-human PDGFRA-PE (Abcam, 1:10), anti-mouse KDR-Alexa647 (BioLegend, 1:100) or anti-human KDR-Alexa647 (BioLegend, 1:30), or anti-human KIT antibody (BD Bioscience, 1:10) for 45 min at room temperature. After washing with FACS buffer, cells were suspended with the secondary antibody solutions containing donkey anti-goat IgG Alexa 488 conjugated (Invitrogen, 1:500) and incubated at room temperature for 30 min. Dead cells were excluded by the gating of DAPI staining. Cells were sorted using FACS ARIA II or analysed by LSR Fortessa II or FACS Canto II (BD Biosciences) with FACSDiva 7.0 software. Sorted cells were plated on 0.1% gelatin-coated culture dishes with differentiation media based on StemPro34 (mouse ESCs), RPMI (human ESCs/iPSCs) with the growth factors shown above, or DMEM/10% FBS in the cases of mouse endothelial differentiation. For staining with anti-TNNT2 antibody (Hytest, 1:200), anti-mouse PECAM1 (BD Biosciences, 1:30), anti-human PECAM1 (Affymetrix, 1:30), anti-ACTA2 (Abcam, 1:100), cells were fixed and permeabilized by using DAKO Intra stain kit (DAKO) according to the manufacturer's instructions. For staining the mouse differentiating ES cells with anti-HCN4 (Millipore, 1:100), anti-TBX5 (SIGMA, 1:30), anti-ISL1 (DSHB 39.4D5, 1:100), anti-NKX2-5 (Santa Cruz, 1:50), firstly the cells were harvested by 3mM EDTA and treated with anti-GFRA2 antibody as described above. Then the cells were fixed and permeabilized by using DAKO Intra stain kit. The cells were incubated with those first antibodies at 4 °C overnight, then incubated with Alexa Fluor secondary antibodies. FACS Canto II was used for the analyses.

CRISPR/Cas9 mediated genome editing

The single guide RNA (sgRNA) sequences were designed with CRISPR Design Tool and sgRNA Designer as previously described (http://www.genome-engineering.org/crispr/) (Hsu et al., 2013), (http://www.broadinstitute.org/rnai/public/analysis-tools/sgrna-design) (Doench et al., 2014). To minimize off-target mutations, we selected the 20 bp genome target where at least 5 bases of the 5' end and 3 bases of the 3' end were distinct from any other sequences in the mouse genome based on BLAST searches (Fu et al., 2013). The synthesised oligonucleotides were cloned into the pX330 vector (Addgene) and were then co-transfected with pIRES-PURO (Clontech) into ESCs using FuGENE HD

(Roche) (Cong et al., 2013; Wang et al., 2013). Puromycin resistant clones were usually isolated 7 days after the initiation of puromycin selection and mutations in each clone were confirmed by Sanger sequencing of PCR products for the genomic region around the target sequences. Among the homozygously mutated clones, deletions/insertions of multiples of 3 bases were discarded because the possibility of in-frame translation of functional proteins could not be excluded. We confirmed the early termination of the translation into proteins by frame-shift mutations and selected at least 2 different clones for each gene. To exclude the possibility that any off-target effects might be responsible for the observed phenotypes, we designed two independent sgRNAs for each target gene. For direct genome editing in mouse embryos, we microinjected or electroporated sgRNAs and *Cas9* mRNAs into the fertilized eggs as previously described (Hashimoto and Takemoto, 2015; Wang et al., 2013). In brief, we harvested the fertilized eggs of F1 (C57BL/6 xCBA/ca) mice, and *in vitro* synthesized 100 ng/μL of sgRNA and 50 ng/μL of Cas9 mRNA were injected into one-cell stage embryos simultaneously (Wang et al., 2013). Alternatively, to introduce sgRNA and Cas9 mRNA, electroporation was performed as previously described (Hashimoto and Takemoto, 2015). One-cell stage F1 (C57BL/6 xC3H) embryos were placed in the 1 mm gap of the electrode (LF510-PT1-5, BEX) filled with 2.5μl of 1200 ng/μl *Cas9* mRNA and 200 ng/μl sgRNAs in Opti-MEM I medium (Gibco). The electric condition applied was two sets of three pulses (Voltage, 25 V; duration, 3 msec; interval, 50 msec; polarity change between the sets) generated by CUY EDIT II electroporator (BEX). Embryos were harvested at E8.5 and E17.5 after oviduct transfer of injected embryos. Yolk sac or tail tissues were subjected for the genome preparation to determine the genotype. PCR primers, sequencing primers and sgRNA design are listed in the table below. WT littermates determined by Sanger sequencing were used as controls in E8.5, and *mCherry* sgRNA plus *Cas9* mRNA transduced embryos were used as controls in E17.5.

Oligonucleotides for CRISPR/Cas9 mediated gene targeting

*Gfra1*_ES_1	F<br>R	CACCGCAGGCTGTCAGATATATTC<br>AAACGAATATATCTGACAGCCTGC
*Gfra2*_ES_1	F<br>R	CACCGCCTGTGACCTCCCGCCTCT<br>AAACAGAGGCGGGAGGTCACAGGC
Ret	F<br>R	CACCGCAGGTGTTCGATGCAGATG<br>AAACCATCTGCATCGAACACCTGC
GDNF	F<br>R	CACCGATGAAGTTATGGGATGTCG<br>AAACCGACATCCCATAACTTCATC
Neurturin	F<br>R	CACCGCGCTGGAAGGCAGCGGCCC<br>AAACGGGCCGCTGCCTTCCAGCGC
*Gfra2*_ES_2	F<br>R	CACCGCTCCAGGTACCGCACCCTT<br>AAACAAGGGTGCGGTACCTGGAGC
Cas9 mRNA synthesis	F<br>R	TTAATACGACTCACTATAGGGAGAATGGACTATAAGGACCACGAC<br>GCGAGCTCTAGGAATTCTTAC
Gfra1 sgRNA_1	F<br>R	TTAATACGACTCACTATAGGCAGGCTGTCAGATATATTC<br>AAAAGCACCGACTCGGTGCC
Gfra2 sgRNA_1	F<br>R	TTAATACGACTCACTATAGGCCTGTGACCTCCCGCCTCT<br>AAAAGCACCGACTCGGTGCC
Gfra1 sgRNA_2	F<br>R	TTAATACGACTCACTATAGTCAAGGCCTCCATAGCGCTG<br>AAAAGCACCGACTCGGTGCC
Gfra2 sgRNA_2	F<br>R	TTAATACGACTCACTATAGTCTTCATGCCCCGCTTGCAG<br>AAAAGCACCGACTCGGTGCC
Gfra2 sgRNA_3	F<br>R	TTAATACGACTCACTATAGGCCAGCCCTTCCTCTCCGCA<br>AAAAGCACCGACTCGGTGCC

PCR/Sequencing primers for genotyping

Gfra1<br>PCR_1	F<br>R	CTAACAGAACTAGGGCCGCT<br>GCCACCTACAGACAGCATTG
Gfra1<br>seq_1	F<br>R	TTCCGGATGGGCAGTGAAAC<br>ACCTCCCTCCAGGCACTTCC
Gfra2<br>PCR_1	F<br>R	AGTTTAGTCCTGGGTCTGCC<br>AACCGAGGATCCAGAGAAGC
Gfra2<br>seq_1	F<br>R	TGCCACTAGGCACACCTGCT<br>CCCCAGGGGTGAGAGGAAAC
Ret<br>PCR	F<br>R	CCCATGCTGCTTTGACCAG<br>GTTTACTTACTGGTGGGCGG
Ret<br>seq	F<br>R	CCTGCGCTGACTGGCACTCT<br>GGGCCTGGAATGGGTGTGCT
Gdnf<br>PCR	F<br>R	AAGTTGGAGGCTGGAGGAC<br>GGGTTTTAGCAAGGCACAGT
Gdnf<br>seq	F<br>R	TCTGAGCCTAACTTGCCTGG<br>GGGAGGGAACGGTTCTTACA
Neurturin<br>PCR	F<br>R	GGGTAAGATAGGAGGTGGCC<br>GTTCAGGAGGCAGGAGGATT
Neurturin<br>seq	F<br>R	CACTGTCCTCCCTCCATACC<br>CCCCAGGCACCTTTTCCA
Gfra2<br>PCR_2	F<br>R	AGGCCTACATTGGGGTCAGAGAC<br>TGGCCAGATCCATCCACCCC
Gfra2<br>seq_2	F<br>R	AGGCCTACATTGGGGTCAGAGAC<br>GGCCAGATCCATCCACCCC
Gfra1<br>PCR_2	F<br>R	CTGGAATCAGGGTACCCACTAGTCTGC<br>GACTGAGGTTGCCTCTGGCCTTC
Gfra1<br>seq_2	F<br>R	ATGTCGGCCGAGGTGAGT<br>CAGCGGCAGTTGTAGAGAGA
Gfra2<br>PCR_3	F<br>R	AGGTTGGGTCTACGTTGACCTTGGTC<br>AAGTTCTTCCTTCCTTCTGGATCCCTG
Gfra2<br>seq_3	F<br>R	TAGGCCTACATTGGGGTCAG<br>CCCAGATGGATGCTCCAAT

Western blot analysis

Cells were harvested with RIPA buffer with protease inhibitors then provided for sonication. After centrifugation at 14,000 rpm for 15 min, the supernatants were mixed with sample buffer (BioRad) with β-mercaptoethanol and boiled at 95 °C for 5 min. Protein concentrations were determined by the Lowry method and 5 μg of the samples were used for blotting. NuPAGE Bis-Tris 4-12% gradient gel (Thermo Scientific) and PVDF membrane (GE Healthcare) were used for electrophoresis and wet transfer. After blocking with 5% skimmed milk/TBS for 60 min, the primary antibodies, anti-GFRA1, anti-GFRA2, anti-RET, anti-GDNF, anti-Neurturin (all were purchased from Abcam), anti-phospho-FAK, anti-FAK, anti-phospho-ERK1/2, anti-ERK1/2 (Cell Signaling Technologies), anti-phospho-Fyn, anti-Fyn (Abcam), anti-TUBA1A (αTubulin; Abcam) were suspended with Can Get Signal solution 1 (TOYOBO), and membranes were incubated with antibodies at 4 °C overnight. Next day, membranes were incubated for 2 hours at room temperature in 5% skimmed milk containing horseradish peroxidase conjugated secondary antibodies (Thermo Scientific). Blots were visualized using Super Signal West Dura (Thermo Scientific) and scanned with an Alpha Imager HP Imaging System (Alpha Innotech) or ChemiDoc Touch (BioRad).

Culture of clonally FACS-isolated cells

Single cell sorting of human CPs was performed by FACS ARIA II according to manufacturer's instructions. Sorted cells were plated on mitomycin C treated mouse embryonic fibroblasts with 20% FBS/RPMI in 96-well plates. 5 μM of Y-27632 ROCK inhibitor was added overnight after sorting, then cells were cultured for a further 2 weeks with 20% FBS/RPMI for expansion and differentiation.

Reverse transcription and quantitative real-time PCR analysis

The total RNAs were extracted using an RNeasy mini kit (Qiagen) from each stage of differentiating ESCs or FACS-purified cells. 500 ng of total RNA for each specimen was used for reverse

transcription using SuperScript® II (Life Technologies) and oligo dT primers. Quantitative PCR was conducted in total volumes of 10 μL (0.25 μL of cDNA solutions) using a SYBR® Green PCR kit (Qiagen) and Rotor Gene 6000. PCR cycles were 95 °C for 10 s, 60 °C for 10 s, and 72 °C for 10 s according to the manufacturer's protocol. For conventional RT-PCR analyses, 50 ng of total RNAs were used for reverse transcription with High-Capacity cDNA reverse transcription kit (Thermo), and 0.5 μL of 20 μL cDNA solutions were provided for 35 to 45 cycles PCR with HotStar Taq (Qiagen). Sequences of the primers are provided in the Supplemental Table. Relative expression levels were calculated using the comparative CT method. *GAPDH* was used as a constitutively expressed internal control.

Mouse qPCR and RT-PCR primers

Gapdh	F<br>R	aactttggcattgtggaagg<br>acacattgggggtaggaaca
Gfra2	F<br>R	tttaacatgatcttggcaaacg<br>agcggagggtttcgtctaa
Tbx5	F<br>R	gagaatggtgtctctggccc<br>ttataggggtgctccgtgct
Nkx2-5	F<br>R	cggtgaccctgacccagccaa<br>gtagacctgcgcctgcgagaa
Mesp1	F<br>R	cagtccctcatctccgctct<br>gttgcattgtcccctccact
T	F<br>R	cagcccacctactggctcta<br>gagcctggggtgatggta
Myl2	F<br>R	ggacacatttgctgccta<br>atcgtgaggaacacggtga
Gfra1	F<br>R	ttcccacacacgttttacca<br>gcccgatacattggatttca
Hcn4	F<br>R	ccatcaatggcatggtga<br>ccttgaagagggcgtagga
Isl1	F<br>R	ccctctcagtcccttgcat<br>gtcttctcgggctgtttgtg

Human qPCR and RT-PCR primers

GAPDH	F<br>R	agccacatcgctcagacac<br>gcccaatacgaccaaatcc
GFRA2	F<br>R	gcttggggaccagtgtcat<br>ctttggagttgttggccttc
TBX5	F<br>R	cgattcgaaacccgagag<br>gaaacactttgattccctcca
NKX2-5	F<br>R	gccttctatccacgtgccta<br>cctctgtcttctccagctcc
MYL2	F<br>R	gcaggcggagaggttttc<br>agttgccagtcacgtcagg
ISL1	F<br>R	aaggacaagaagcgaagcat<br>ttcctgtcatcccctggata
HCN4	F<br>R	ggtgtccatcaacaacatgg<br>gccttgaagagcgcgtag
TNNT2	F<br>R	gtcggcagctgctgttct<br>tcctctctccagtcctcctct
PECAM1	F<br>R	gcaacacagtccagatagtcgt<br>gacctcaaactgggcatcat
MYH11	F<br>R	aactcgtgtccaacctggaa<br>ttctcctcggctaacaactga

Transmission Electron Micrographs

Dissected 1 mm^3 tissue blocks from the hearts of E17.5 embryo were fixed in half Karnovsky fixative (1% of glutaraldehyde/1% of PFA/0.1 M of sodium phosphate, pH 7.3) for 2 h at 4 °C and post-fixed

in 2% of osmium tetroxide//0.1 M of sodium phosphate (pH 7.3) for 2h at 4°C. After dehydration with ethanol, the specimens were incubated in propylene oxide, and embedded Quetol 812 (Nisshin EM). Ultra-thin sections were stained with uranyl acetate and lead citrate, and observed with transmission electron microscope (Hitachi, H-7650) at 80 kV.

Statistical Analysis

Quantitative data are presented as means ± SEM (unless indicated otherwise) and were compared by using a Student's t test or one-way ANOVA. *P* values of <0.05 were considered as statistically significant.

SUPPLEMENTAL REFERENCES

Kurimoto, K., Yabuta, Y., Ohinata, Y., Ono, Y., Uno, K.D., Yamada, R.G., Ueda, H.R., and Saitou, M. (2006). An improved single-cell cDNA amplification method for efficient high-density oligonucleotide microarray analysis. Nucleic Acids Res *34*, e42.

Somers, A., Jean, J.C., Sommer, C.A., Omari, A., Ford, C.C., Mills, J.A., Ying, L., Sommer, A.G., Jean, J.M., Smith, B.W.*, et al.* (2010). Generation of transgene-free lung disease-specific human induced pluripotent stem cells using a single excisable lentiviral stem cell cassette. Stem Cells *28*, 1728-1740.

Uehara, M., Yashiro, K., Takaoka, K., Yamamoto, M., and Hamada, H. (2009). Removal of maternal retinoic acid by embryonic CYP26 is required for correct Nodal expression during early embryonic patterning. Genes Dev *23*, 1689-1698.
